# Effectiveness of computer-based interventions for community-dwelling people with cognitive decline: a systematic review with meta-analyses

**DOI:** 10.1186/s12877-023-03941-y

**Published:** 2023-04-12

**Authors:** Julia Zuschnegg, Daniela Schoberer, Alfred Häussl, Sereina A. Herzog, Silvia Russegger, Karin Ploder, Maria Fellner, Maria M. Hofmarcher-Holzhacker, Regina Roller-Wirnsberger, Lucas Paletta, Marisa Koini, Sandra Schüssler

**Affiliations:** 1 Institute of Social Medicine and Epidemiology, Medical University of Graz, Neue Stiftingtalstraße 6, Graz, 8010 Austria; 2 Institute of Nursing Science, Medical University of Graz, Neue Stiftingtalstraße 6, 8010 Graz, Austria; 3 Department of Psychiatry and Psychotherapeutic Medicine, Medical University of Graz, Auenbruggerplatz 31, 8036 Graz, Austria; 4 Institute for Medical Informatics, Statistics and Documentation, Medical University of Graz, Auenbruggerplatz 2, 8010 Graz, Austria; 5 DIGITAL – Institute for Information and Communication Technologies, JOANNEUM RESEARCH Forschungsgesellschaft mbH, Steyrergasse 17, 8010 Graz, Austria; 6 Austrian Red Cross Organization, Styrian Branch, Merangasse 26, 8010 Graz, Austria; 7 digitAAL Life GmbH, Schuberststraße 6a, 8010 Graz, Austria; 8 HS&I Health System Intelligence, Josefstädter Straße 14/60, 1080 Vienna, Austria; 9 Department of Internal Medicine, Research Unit Aging and Old Age Medicine, Medical University of Graz, Auenbruggerplatz 15, 8036 Graz, Austria; 10 Department of Neurology, Division of Neurogeriatrics, Medical University of Graz, Auenbruggerplatz 22, 8036 Graz, Austria

**Keywords:** Subjective cognitive decline, Mild cognitive impairment, Dementia, Prevention, Non-pharmacological treatment, Cognition, Computerized cognitive training, Computer-based cognitive training, Virtual reality, meta-analysis

## Abstract

**Background:**

Cognitive deficits arise with age and can increase the risk for subjective cognitive decline (SCD) and mild cognitive impairment (MCI), which may result in dementia, leading to health problems, care dependency and institutionalization. Computer-based cognitive interventions (CCIs) have the potential to act as important counteraction functions in preserving or improving cognition concomitant to available pharmacological treatment. The aim was to assess the effectiveness of CCIs performed individually with a personal or tablet computer, game console, virtual, augmented, or mixed reality application on cognition in community-dwelling people with SCD, MCI and dementia.

**Methods:**

A systematic review with meta-analyses of randomized controlled trials (RCTs) was performed. The systematic literature search was conducted in MEDLINE, CINAHL, Embase, Cochrane CENTRAL, IEEE Xplore Digital Library, Web of Science, Scopus and PsycINFO. In addition, a search for gray literature and backward citation searching were carried out. To judge on the evidence, two reviewers independently used the Cochrane Risk of Bias Tool. The standardized mean difference (SDM) for pooling comparable studies using the random-effects model was applied.

**Results:**

Twenty-four RCTs were identified, of which 1 RCT examined CCIs in individuals with SCD, 18 RCTs with MCI, and 6 RCTs with dementia. Most interventions were conducted with personal computers. Meta-analyses with 12 RCTs showed significant effects of computer-based cognitive interventions for people with MCI in the domains memory, working memory, attention/concentration/processing speed and executive functioning, but no significant improvements in global cognition and language. Regarding dementia a meta-analysis pooled with 4 RCTs demonstrated a tendency towards, but no significant increase of memory functions (SMD 0.33, CI 95% [-0.10, 0.77]). One RCT regarding SCD reported significant improvements in memory functions for participants conducting a cognitive training on a personal computer.

**Conclusions:**

The results demonstrated that CCIs have beneficial effects on domain-specific cognition in people with MCI but no significant effects on people with dementia. In terms of SCD, one study showed significant improvements in memory functions. It seems that the beneficial effect for cognitive preservation or improvement due to CCIs occurs at the earliest intervention state. However, more research on SCD is needed.

**Trial registration:**

PROSPERO International Prospective Register of Systematic Reviews CDR42020184069.

**Supplementary Information:**

The online version contains supplementary material available at 10.1186/s12877-023-03941-y.

## Introduction

Aging is associated with cognitive decline [1]. However, when cognitive capacities deteriorate beyond an ageing-associated normal level, cognitive decline can range from subjective cognitive decline (SCD) to mild cognitive impairment (MCI) and finally to dementia [2]. Cognition is responsible for all activities and processes concerned with the acquisition, storage, retrieval and processing of information [3, 4]. It includes different cognitive processes or domains (e.g. memory, attention) [4]. The progressive loss of cognitive capacity leads to various health problems, care dependency and institutionalization over time, particularly in dementia [2].

Dementia is a progressive disease and one of the world's leading causes of disability, associated with high financial, emotional and societal burdens [2, 5]. About 50 million people worldwide live with dementia and this figure is likely to rise to about 152 million people by 2050 [6]. Moreover, the parallel increasing number of people living with SCD and MCI face a higher risk of developing dementia, adding further to the challenges to be faced in the future, as treatment, care dependency and financial costs all rise [2, 7–9]. It is estimated that with a prevalence of between 23.8% and 25.6%, one in four people (above 60 years and older) are affected by SCD, self-experiencing a cognitive decline without an objective cognitive impairment [10]. A meta-analysis indicated a future decline of SCD into MCI of 27% and a 14% decline into dementia [8, 9]. People with MCI already showing impaired cognitive abilities and the prevalence of those aged 60 years and older is estimated between 15% and 20% with an annual rate of between 8% and 15% at which MCI progresses to dementia [7].

Faced with these conditions of cognitive decline, pharmacological treatments currently have a limited effect on the progression of the underlying disease, and this is the reason why non-pharmacological interventions such as cognitive interventions, have moved into the foreground [2, 11, 12]. Cognitive interventions have the aim of preserving or improving cognitive processes or address the impact of impairment in cognitive processes on associated functional abilities in activities of daily living (ADL) (e.g. dressing, personal hygiene) and instrumental ADL (IADL) (e.g. meal preparation, managing medication) [2, 13]. Such interventions usually follow a specific cognitive approach, for which literature definitions often overlap due to their underlying theoretical assumptions and core elements, as well as the context or population for which they were developed [13]. Nevertheless, key defining features exist for the most common approaches, which are cognitive training (CT), cognitive rehabilitation (CR) and cognitive stimulation (CS) [14]. Besides the common goals to preserve or improve (specific) cognitive abilities and processes, there are some differences [14]. CT involves repeated guided practices with standardized, structured tasks, which are usually based on theoretically motivated strategies with a range of (adaptive) difficulties [13, 15–17]. CR typically focusing on a person’s need with individualized goals for which patients work together with healthcare professionals and family, following a more compensatory approach to perform individually relevant everyday tasks [13, 15–17]. CS includes a wide range of activities to stimulate thinking and multiple cognitive domains with the involvement of, for example, reality orientation (e.g. relating to time and place) or reminiscence therapy (e.g. telling others about one’s past experiences) [13, 15–17].

Cognitive interventions can be delivered as individual or group sessions, with family members or experts as support persons (e.g. nursing scientists, therapists) [14]. They are available in paper form, but also as computer-based cognitive interventions (CCIs) [14]. CCIs have increasingly replaced original paper-and-pencil formats, as they have several advantages over those traditional techniques [18]. For instance, training tasks can be directed to specific cognitive domains (e.g. memory); they can be personalized and adjusted to the performances of an individual; they can be designed in a highly immersive and enjoyable form; and they can incorporate immediate quantitative feedback [18]. Standard devices, such as personal computers (PCs), tablet computers (hereafter called ‘tablets’) and gaming consoles are already used as technologies for CCIs [17]. More recently, emerging technologies such as virtual reality (VR), which are characterized by novelty, growth and potential socio-economic impact, are on the rise [19, 20].

Systematic reviews with meta-analyses [17, 19, 21, 22] already demonstrated that such CCIs have the potential to improve global cognition and selected cognitive domains in older persons with cognitive decline. However, there were at least three points, which were not sufficient considered in those reviews. First, they only included studies either with standard devices [17, 21, 22] or emerging technologies [19]. For that reason, we decided to include a comprehensive range of technologies used for cognitive purposes in our systematic review, covering both already existing technologies (i.e. PCs, tablets/smartphones, gaming consoles), as well as emerging technologies (i.e. virtual, augmented, and mixed reality). Second, the aforementioned systematic reviews on CCIs [17, 19, 21, 22] did not differentiate between participants living at home or in institutional care settings. Since it is not only the priority of healthcare systems to strengthen home care, but also to maintain independence for living at home as long as possible and to delay institutional care of individuals most affected, it is important to consider closed evidence related to this setting [2, 23]. We thus restricted the setting to people living at home and, in this regard, defined the training format of CCIs to single sessions. Third, the condition of SCD was not considered in those systematic reviews [17, 19, 21, 22], nor could a review focusing on this target group be identified. Consequently, we decided to include this relevant early stage of cognitive decline in our systematic review.

To the best of our knowledge, there has been no systematic review until now, which exclusively considers community-dwelling people with SCD, MCI and dementia in all three cognitive approaches (CT, CR, CS), performed on an individual basis using PCs, tablet/smartphones, gaming consoles, virtual, augmented or mixed reality. Therefore, this systematic review addressed the following research question: How effective are individually performed CCIs for community-dwelling people with SCD, MCI and dementia on cognition?

## Methods

### Design

This systematic review and meta-analyses, as part of a comprehensive realist review, was reported according to the Preferred Reporting Items for Systematic Reviews and Meta-Analyses (PRISMA) Statement [24]. The protocol was registered at the International Prospective Register of Systematic Reviews (PROSPERO, CDR42020184069).

### Eligibility criteria

The PICO-framework [25] (i.e. Participants, Interventions, Comparison, Outcome) was used to determine inclusion and exclusion criteria for this systematic review. Randomized controlled trials (RCTs) (including conference articles) with the following criteria were included:

#### Population

We included community-dwelling adults (i.e. people living at home and not in healthcare institutions) over the age of 18 years with SCD, MCI (any type), or dementia (any type or stage). The conditions were defined as follows:

- SCD is a self-perceived decline in any cognitive domain over time, which is unrelated to an acute event or disease, with a normal age-, gender-, and education-adjusted performance on standardized cognitive tests [26].
- MCI manifest through cognitive decline or impairment, with an objective evidence of impairment in cognitive domains, with the absence of dementia and essentially normal functional activities [27].
- Dementia is typically caused by age-related pathophysiological processes related to cognitive functions, which affects a person’s ability to perform (I)ADL [2]. Different causes of dementia (e.g. Alzheimer’s disease, cerebrovascular disease) are diagnosed by physicians [2].

The studies at least had to describe that the relevant condition was diagnosed and/or had to describe the diagnostic procedure in association with the diagnostic criteria and/or give reference to established clinical or research diagnostic criteria. Data from studies including different groups presenting with cognitive decline, had to be presented in a way to enable data extraction for the group(s) of interest.

#### Intervention

All interventions that met our defined cognitive approaches of CT, CR, or CS [13, 15–17] exclusively or in combination with physical activity, which were conducted with standard (i.e. PCs, tablets/smartphones, game consoles) and emerging technologies (i.e. virtual, augmented or mixed reality) were included. Robots also constitutes a promising emerging technology [20] and are already tested as CCI [28]. However, the goal of assistance by robots is to create a close and effective interaction with a human user through conversations, emotions, and gestures, which the other chosen emerging technologies do not cover [29]. For reasons of heterogeneity [30], robots were therefore not considered in our review.

Due to our focus on people living at home, only individual sessions of computer-based cognitive interventions were eligible. In this regard, we also consider interventions which were conducted in a lab setting (e.g. adult daycare center, outpatient clinic). No restrictions were made regarding intervention dose, including the overall duration of the intervention or number of intervention sessions. In terms of studies combining computer-based interventions with other kind of cognitive interventions (e.g. paper and pencil forms), the results had to be reported in a way that enabled extracting the data for the intervention(s) of interest. No restriction was set on standard pharmacological treatment.

#### Control

We included studies with no specific intervention or another kind of (computer-based) cognitive training as control intervention.

#### Outcome

For this systematic review continuous data of objective outcome measurements on global and domain-specific cognition (i.e. memory, working memory, attention, concentration, processing speed, executive functioning, language, visuospatial, and constructional abilities) was considered.

### Information sources and search strategy

In the following databases a systematic literature search was performed by the first author (JZ) to April 2020: MEDLINE via PubMed, CINAHL via Ovid, Embase via Ovid, Cochrane CENTRAL via Ovid, IEEE Xplore Digital Library, Web of Science, Scopus and PsycINFO. Gray literature and additional publications were screened in google scholar and on the social media platform Research Gate (Additional file 1). Finally, the search was completed by checking citations of included studies and identified reviews.

A search strategy, with a combination of keywords and controlled vocabulary terms like MeSH headings using Boolean operators was developed. Following the recommendations of Lefebvre [31], no timeframe, language or document format restriction was set during the databases search to ensure that the search captured as many studies as possible that meet the eligibility criteria. However, only studies written in English or German were finally included.

### Study selection

The search hits of each database were inserted into the bibliographic management program EndNote X8 and duplicates were removed. Title-abstract, as well as a full text screening process was based on the inclusion criteria and was conducted independently by JZ, SD, AH at each stage, with JZ assessing all the articles, and the other two authors assessing one half of the articles each. In unclear cases, inclusion was discussed and agreed upon within the research team.

### Data extraction

A standardized data extraction form was used to extract general study information (e.g. authors, publication date) and relevant data of the participants' characteristics, interventions and outcomes (see Additional file 2). The process of data extraction was performed by JZ and was checked independently by AH for accuracy. Any disagreements between the authors during this process were solved by discussion and consensus. In case of uncertainty, the authors DS and SS were consulted.

### Study risk of bias assessment

The methodological quality of all included studies was assessed independently by JZ and AH using the Cochrane Risk of Bias tool for RCTs [32]. Bias for each study were rated with a high, low, or unclear risk for the following domains: random sequence generation, allocation concealment, blinding of participants and personnel, blinding of the outcome assessment, incomplete outcome and other source of bias. JZ and AH compared and discussed their critical appraisal assessments and disagreements were resolved by consensus or by consulting DS or SS.

### Data synthesis

Data synthesis was carried out following the Cochrane Handbook [33] and Borenstein et al. [34] and was discussed within the research team.

Meta-analyses were performed with the statistical software R (version 4.2.2) [35] and meta package (version 6.1–0) [36], using an inverse variance random-effects model with Hartung-Knapp adjustment [37, 38]. The random-effects model was chosen as it is more in line with the actual sample distribution and allows the conclusions to be generalized to a wider array of situations since this gives a better reflection of the ‘real world’ [39].

Standardized mean differences (SMD) with 95% confidence interval (CI) were applied to pool post-intervention values [30] from studies with similar outcome measures, populations, and technologies. The definition of SMD used in the analysis is Hedges’ (adjusted) g, which is similar to Cohens’ d, but includes an adjustment for small sample bias [40]. Values of 0.15, 0.40, and 0.75 for Hedges’ g are considered of small, medium, and large effect sizes [41], constituting important indicators for clinical significance of statistically significant results, as it reflects the magnitude of the difference in outcomes between groups [42, 43].

Data from the studies included were classified by MK and JZ respectively into global cognition or into the following cognitive domains: memory, working memory, attention/concentration/processing speed, executive functioning, language, and visuospatial/constructional abilities (Additional file 3). If a study reported multiple measures of the same outcome, a simple composite score (i.e. mean of standardized scores) for the measures was created [44] and used for the meta-analysis.

Comparisons between CCIs and control to outcomes immediate post interventions as well as to follow-up (3 to 12 months) were made.

Tests for heterogeneity were performed and assessed by Chi^2^-statistics and the associated I^2^ statistics, for which an I^2^ from 0% to 40% might not be important, 30% to 60% might represent moderate heterogeneity, 50% to 90% might represent substantial heterogeneity and 75% to 100% represented a considerable heterogeneity [30].

If statistical heterogeneity was present, subgroup analyses or sensitivity analyses were performed [30].

When the reported data from the included studies did not allow pooling, their results were synthesized narratively.

## Results

### Study selection

**Fig. 1:**
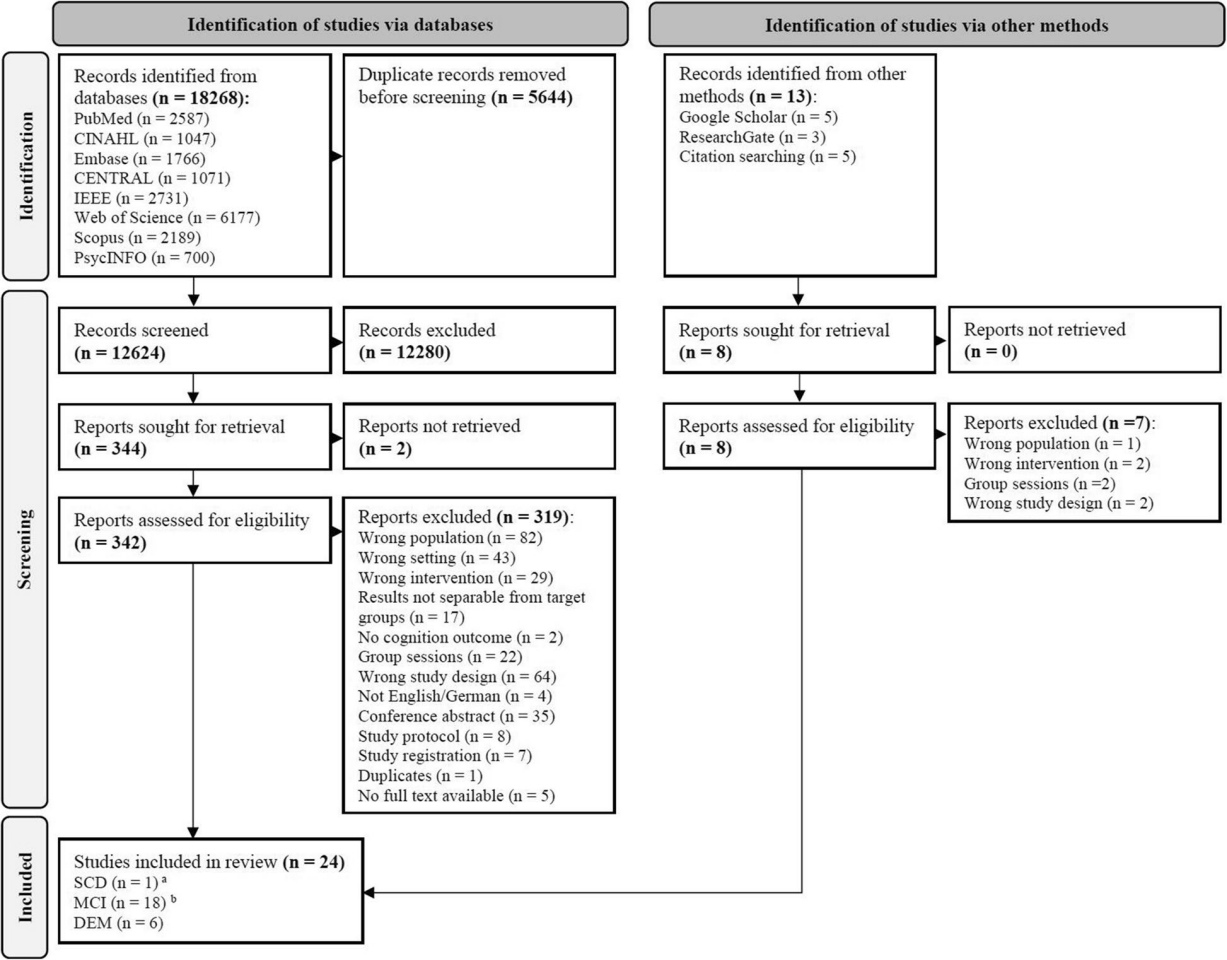
PRISMA flow chart of study selection of this systematic review [24]. **a **One study [45] examined people with subjective cognitive decline (SCD), as well as people with dementia (DEM). **b **MCI: Mild cognitive impairment

The literature search retrieved 18,281 records. After removing duplicates, 12,632 records were screened by title and abstract for their relevance. In total, 350 studies were then subjected to a full-text screening, from which 24 studies were finally included in this systematic review. Figure 1 shows the study selection process with the reasons for exclusion of studies at the full-text screening stage.

### Study characteristics

All the studies were published in English during the period from 1994 to 2020 (Table 1, Additional files 2 and 3). Studies were predominantly conducted in Europe (*n* = 10) and Asia (*n* = 9), followed by North America (*n* = 4) and Oceania (*n* = 1). Most studies investigated interventions according to the concept of CT, while 4 studies [46–49] could be assigned to CR. No study was identified on the concept of CS.

**Table 1:** Characteristics of the included RCTs (*n* = 24)

Authors; Country	Type of cognitive decline	Sample size (IG^a^/CG^b^)	Intervention	Control	Duration in weeks	Sessions/week, (minutes/session)	Setting
Cinar et al. 2020; [45] Turkey	SCD^c^	60 (30/30)	PC^d^	No intervention	12 (or at least 1200 min of training)	about 7 (15–20 min)	Home
Amjad et al. 2019; [50] Pakistan	MCI^e^ - not specified	38 (18/20)	VR^f^	Physical exercises (motion, stretching)	6	5 (20–30 min)	Lab
Barnes et al. 2009; [51] USA	MCI - all types	47 (22/25)	PC	Alternative computer-based activities	6	5 (100 min)	Home
Damirchi et al. 2018; [52] Iran	MCI - not specified	44 (11/11/13/9)	IG1: PC	Waiting list group	8	3 (30 min in weeks 1–6; 60 min in 7^th^ and 8^th^ week)	Lab
			IG2: Physical activity group				
			IG3: IG1 combined with physical group activities				
Dimitriadis et al. 2016; [53] Greece	MCI - not specified	158 (53/50/55)	AR^g^	CG1: Alternative computer-based activity	10	4 (90 min)	Home
				CG2: Waiting list group			
Finn et al. 2011; [54] Australia	MCI - amnestic multiple domain	16 (8/8)	PC	Waiting list group	average of 11.4^ h^	4–5 (not reported)	Home
Flak et al. 2019; [55] Norway	MCI - all types	69 (35/34)^i^	PC	Same intervention as in IG but in contrast with fixed low level of difficulty	5	5 (30–40 min)	Home
Hagovska et al. 2017; [46] Slovakia	MCI - not specified	60 (30/30)	PC	Group cognitive training program	10	2 (30 min)	Lab
Han et al. 2017; [56] South Korea	MCI - all types	43 (43/42)^j^	Tablet	Usual treatment	4	2 (30 min)	Lab
Herrera et al. 2012; [57] France	MCI - amnestic multiple domain	22 (11/11)	PC	Cognitive training	12	2 (60 min)	Lab
Hyer et al. 2016; [58] USA	MCI - amnestic and non-amnestic	68 (34/34)	PC	Same intervention as in IG but in contrast with fixed low level of difficulty	5–7	about 5 (40 min)	Lab or Home
Li et al. 2019; [59] China	MCI - amnestic (due to AD^k^)	141 (78/63)	PC	No intervention	24	3–4 (about 40 min)	Home
Lin et al. 2016; [60] USA	MCI - amnestic multiple domain (due to AD)	21 (10/11)	PC	Cognitive training	6	4 (60 min)	Home
Nousia et al. 2019; [48] Greece	MCI - all types	46 (25/21)	PC	Usual treatment	15	2 (60 min)	Lab
Park et al. 2019; [49] South Korea	MCI - single and multiple domain	21 (10/11)	Mixed Reality	Computer-based cognitive training	6	3 (30 min)	Lab
Park et al. 2020; [61] South Korea	MCI - amnestic	21 (10/11)	VR	Waiting list group	12	2 (30 min)	Lab
Rosen et al. 2011; [62] USA	MCI - amnestic	12 (6/6)	PC	Alternative computer-based activities	Average of 8^ l^	5 (100 min)	Home
Savulich et al. 2017; [63] United Kingdom	MCI - amnestic (due to AD)	42 (21/21)	Tablet	No intervention	4	not reported (60 min)	Lab
Thapa et al. 2020; [64] South Korea	MCI - not specified	68 (34/34)^m^	VR^n^	Educational program on general health care	8	3 (100 min)	Lab
Cinar et al. 2020; [45] Turkey	Dementia - AD	60 (30/30)	PC	No intervention	12 (or at least 1200 min of training)	about 7 (15–20 min)	Home
Galante et al. 2007; [65] Italy	Dementia - AD	12 (7/5)^o^	PC	Interviews on current affairs and participants’ lives	4	3 (60 min)	Lab
Heiss et al. 1994; [66] Germany	Dementia - AD	70 (18/17/18/17)	IG1: PC	Social support	24	2 (60 min)	Lab
			IG2^p^: IG1 combined with medication				
			IG3^p^: IG1 combined with medication				
Karssemeijer et al. 2019; [67, 68] The Netherlands	Dementia - all types	115 (38/38/39)	IG1: VR (combined with cycling on a stationary bike)	Physical exercises (relaxation, flexibility)	12	3 (30–40 min)	Lab
			IG2: Cycling on a stationary bike				
Lee et al. 2013; [47] China	Dementia - AD	19 (7/6/6)	IG1: Tablet	Waiting-list control group with cognitive activities	6	2 (12–30 min)	Lab
			IG2: Cognitive training without Tablet				
Yu et al. 2015; [69] China	Dementia - not specified	32 (16/16)	Tablet	Cognitive training	4–8	1–2 (30 min)	Lab

^a^IG: intervention group

^b^CG: control group

^c^SCD: subjective cognitive decline

^d^PC: personal computer

^e^MCI: mild cognitive impairment

^f^VR: virtual reality

^g^AR: augmented reality

^h^The authors anticipated 6–10 weeks. Participants completed at least 80% of the sessions

^i^68 (34/34) participants were included in the final analysis of the respectively study

^j^Cross-over randomized controlled trial

^k^AD: Alzheimer’s disease

^l^Participants had to use the program until either achievement of asymptotic performance levels over a several day period or completion of 80% of the training material in a given exercise

^m^66 (33/33) participants were included in the final analysis of the respectively study

^n^The intervention consists of two parts 1) cognitive training 2) educational program on general health care (as in CG)

^o^11 (7/4) participants were included in the final analysis of the respectively study

^p^These intervention groups were not considered for analysis, because the medication used consisted of non-commercial substances for the treatment of dementia, as well as non-registered substances of the Austrian Register of Pharmaceutical Specialties

In the context of participant cognitive conditions in the included studies, one study investigated SCD, 18 studies MCI and 6 studies dementia.

The SCD investigation study [45] had a sample size of 60 participants, with a mean age of 67.4 years. The web-based intervention contained not only CT on a PC but also physical exercises (Table 1, Additional files 2 and 3).

The sample size in the 18 MCI-studies ranged from 12–158 participants, with a total number of 924 participants. The mean age ranged from 66.0–76.6 years. One study [50] did not report any participant characteristics and one [52] recruited only women. CCIs were conducted predominantly with PCs (*n* = 11) [46, 48, 51, 52, 54, 55, 57–60, 62], followed by tablets (*n* = 2) [56, 63], VR (*n* = 3) [50, 61, 64], augmented reality (AR) (*n* = 1) [53] and one study [49] with mixed reality (MR) (*n* = 1), which was a combined intervention of VR and AR with a tablet as device. Two studies [61, 64] used immersive VR-technology, while the third study [50] was non-immersive and based on a gaming console. Most studies had no specific control intervention, a usual or non-cognitive alternative treatment, whereas 3 studies [46, 57, 60] had non-computer-based CT and 3 studies (slightly) different CCIs [49, 55, 58] as control comparator. The longest intervention duration was 24 weeks [59] and the shortest 4 [56, 63] weeks.

The 6 studies focusing on people with dementia encompassed a total of 273 participants with a sample size ranged from 11–115 subjects. The mean age of participants ranged from 66.3–83.0 years and, in 5 studies, global cognition at baseline ranged from 16.6–23.0 points of the Mini Mental State Examination (MMSE) and was 20.0 points in one study [45], utilizing the Montreal Cognitive Assessment (MoCA). Most studies were conducted with a PC [45, 65, 66] or tablet [47, 69], while one study [67, 68] had a non-immersive VR-technology as intervention, consisting of a home trainer which related to a video screen showing a virtual bike tour including cognitive tasks. Only one study [45] reported no alternative treatment for participants in the control group. Duration of intervention ranged from 4–24 weeks.

### Risk of bias

**Fig. 2:**
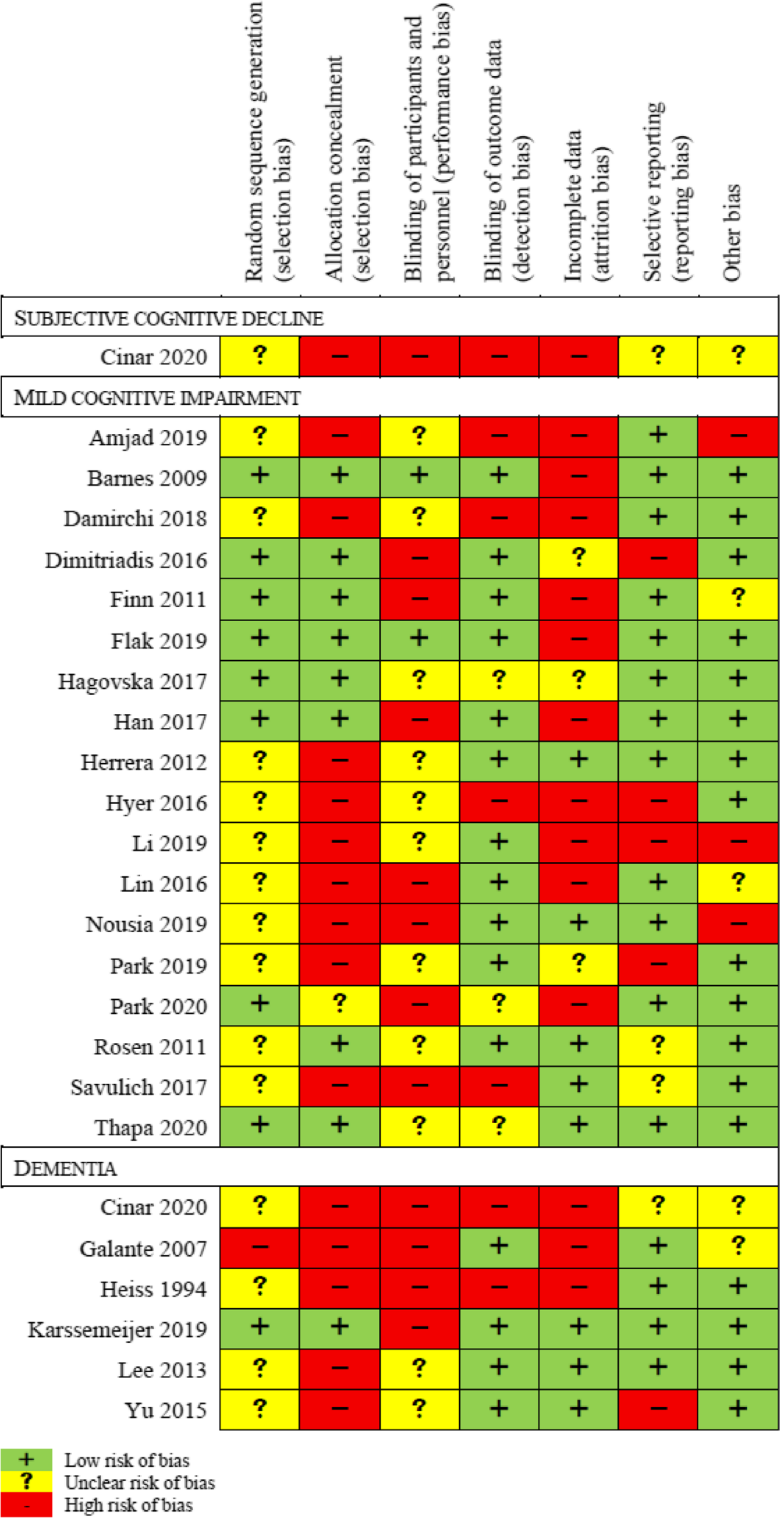
Risk of bias for included studies per target group

Figure 2 provides an overview of the risk of bias for the included studies. Risk of selection bias occurred most frequently in the studies because the method of random sequence generation was not described and was therefore unclear [45, 48–50, 52, 57–60, 62, 63]. Furthermore, the majority of studies [45, 47–50, 52, 57–60, 63, 65, 66, 69] did not mention the procedure of allocation concealment, which was rated with a high risk for selection bias. Overall, only two studies [51, 55], involving people with MCI, were assessed as being at low risk for performance bias. Most of the studies [47–49, 51, 53–57, 59, 60, 62, 65, 67, 69] had a low risk for detection bias by means of blinding the people who measured the outcome data. In contrast the majority of included studies [45, 50–52, 54–56, 58–61, 65, 66] showed a high risk of attrition bias due to insufficient description of the handling of dropouts, as well as missing descriptions of the reasons, inappropriate statistical measures (e.g. last observation carried forward) to compensate missing data, unequal or unclear number of participants between groups, and high drop-out rates. Two-thirds of all studies showed low risk in reporting bias [46–48, 50–52, 54–57, 60, 61, 64–67] and other bias [46, 47, 49, 51–53, 55–58, 61–64, 66, 67, 69], respectively. A high risk of other bias mainly concerned significant differences in one [48] or more [59] relevant baseline characteristics in cognition between the groups, or also due to a significant lack of reporting [50]. Some studies reported cognition baseline data, but were assessed with an unclear risk of bias, due to one [65] or more [54, 60] questionable differences that were not statistically analyzed. One study [45] did not report either statistical information or baseline data regarding a cognitive measurement battery.

### Effects of CCIs on cognition of people with SCD

#### Global Cognition (immediately after Intervention)

Cinar et al. [45] investigated global cognition with the MoCA in people with SCD, which demonstrated a tendency for improvement in the intervention compared to the control group, but with non-significant group differences.

#### Domain-specific cognition (immediately after Intervention)

For people in the intervention group, memory functioning measured with the Cambridge Cognition CANTAB assessment, revealed significant improvements compared to the control group (delayed matching sample (DMS), percent correct, *p* = 0.012; DMS, percent correct, all delays, *p* = 0.019; paired associated learning (PAL), total errors (adjusted), *p* = 0.005; PAL, total errors, 6 shapes adjusted, *p* = 0.02). The pattern recognition memory (PRM), spatial-working memory (SWM) and reaction time (RT) of the CANTAB assessment showed no significant change between the groups [45].

### Effects of CCIs on cognition of people with MCI

#### Global cognition (immediately after Intervention)

**Fig. 3:**
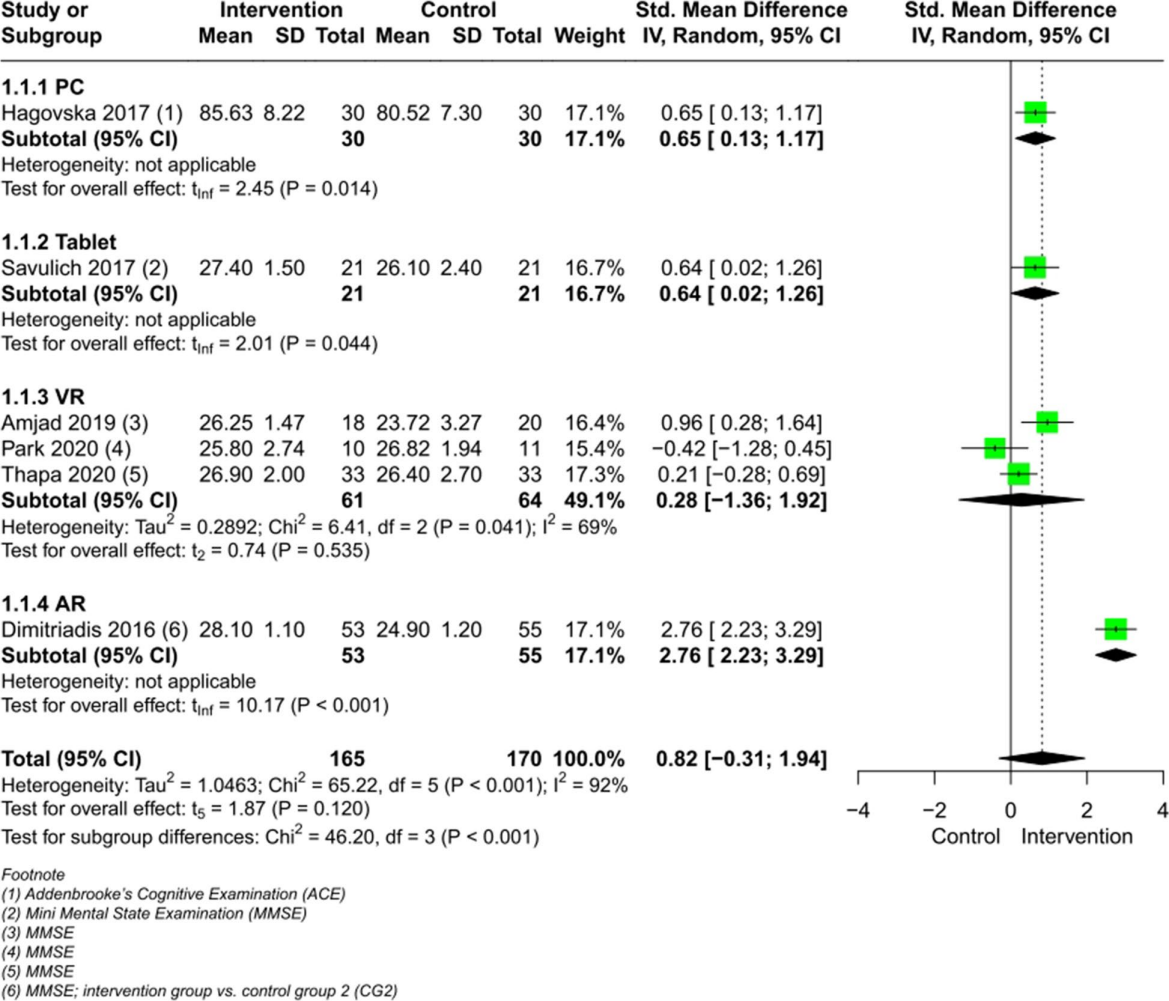
Meta-analysis of CCIs for people with MCI vs. control immediately post intervention on global cognition

The result of the meta-analysis on 6 RCTs (Fig. 3) comparing CCIs vs. a control group post intervention showed a tendency for improvement but had no significant effect on people with MCI regarding global cognition (SMD 0.82, CI 95% [-0.31, 1.94], I^2^ = 92%). Excluding the one study with the AR-intervention [53] with a large SMD, let the heterogeneity drop to I^2^ = 49%, but with an non-significant effect (SMD 0.45, CI 95% [-0.13, 1.03]) (Figure 1 in Additional file 4).

The VR subgroup, including 3 studies, showed a heterogeneity of I^2^ = 69%, which resulted in a trivial heterogeneity (I^2^ = 34%) after excluding the non-immersive VR-technology study [50], but with a maintained non-significant effect (Figure 2 in Additional file 4).

Three studies [51, 56, 59] provided data on the outcome global cognition in such a way, that pooling was not possible. Two of these studies [51, 56] confirmed the non-significant effect. Li et al. [59] investigated global cognition with two instruments, while one was non-significant and the second showed a significant improvement for the intervention group (MMSE, *p* = 0.002).

#### Domain-specific cognition (immediately after Intervention)

**Fig. 4:**
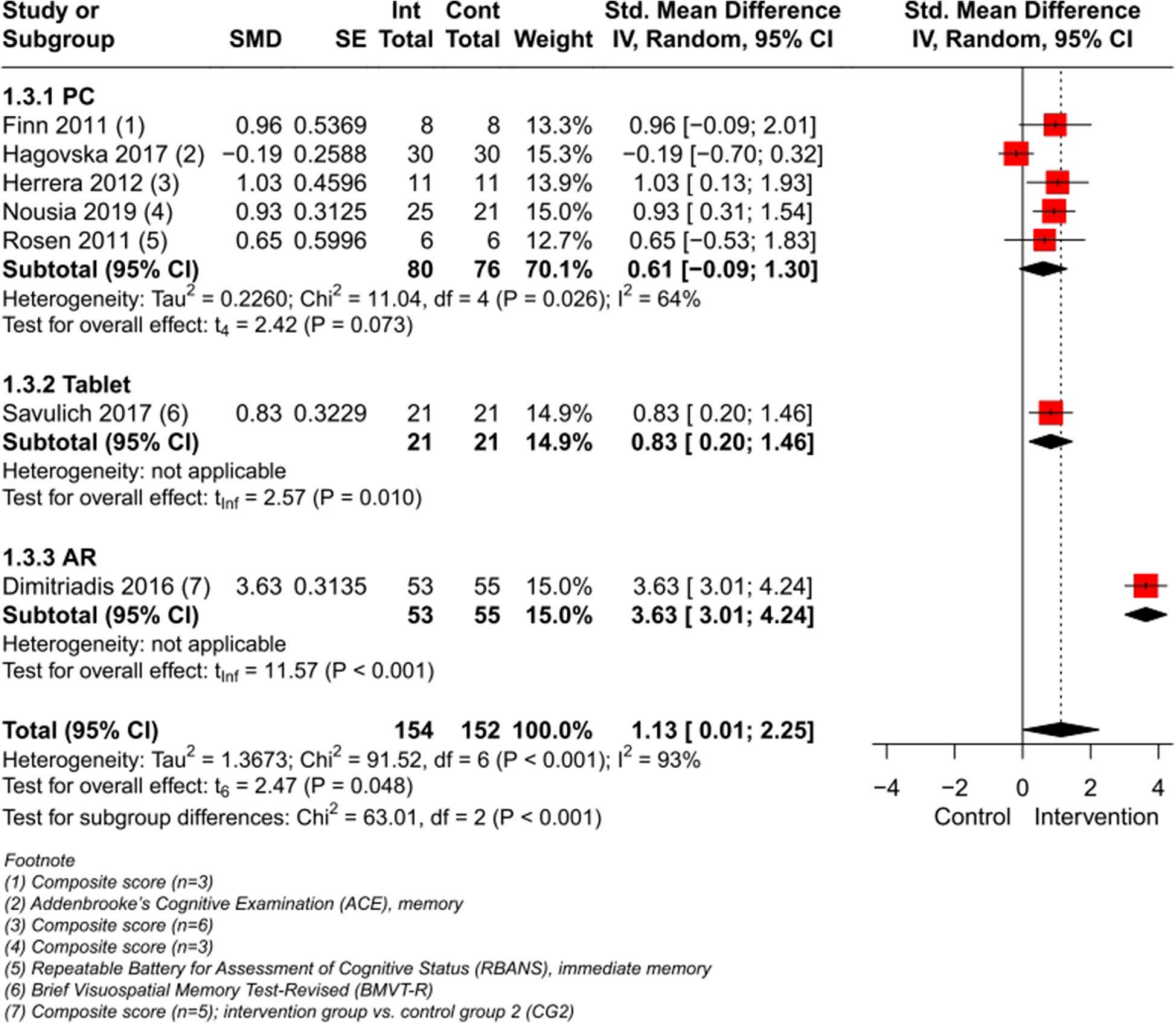
Meta-analysis of CCIs for people with MCI vs. control immediately post intervention on memory

**Fig. 5:**
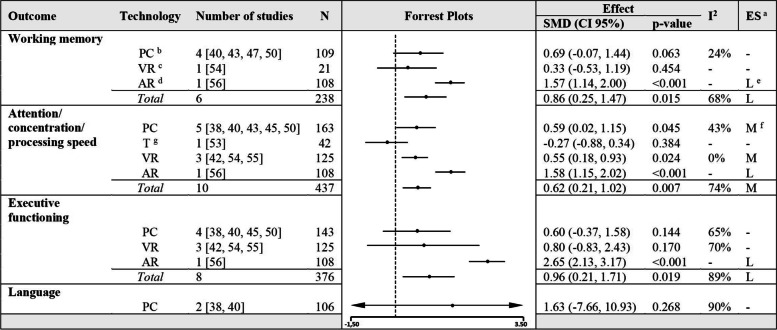
Meta-analyses with subgroups of CCIs on people with MCI vs. control immediately post intervention. **a **ES: effect size; definition of SMD is Hedges’ g, which is categorized in 0.15, 0.40 and 0.75 for small, medium, and large effect sizes [40, 41]. **b **PC: personal computer. **c **VR: virtual reality. **d **AR: augmented reality. **e **L: large effect size. **f **M: medium effect size. **g **T: tablet

**Fig. 6:**
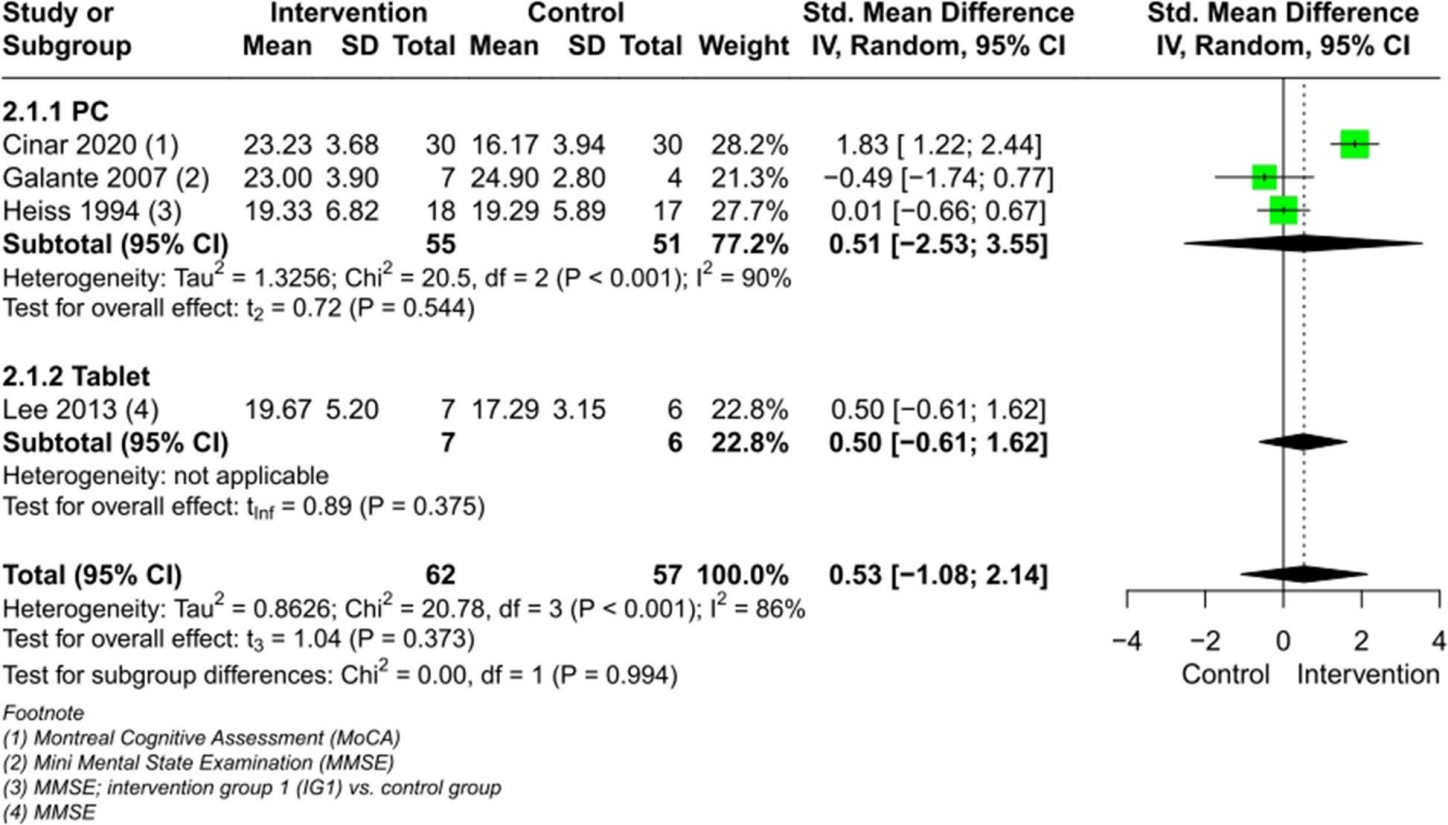
Meta-analysis of CCIs for people with dementia vs. control immediately post intervention on global cognition

**Fig. 7:**
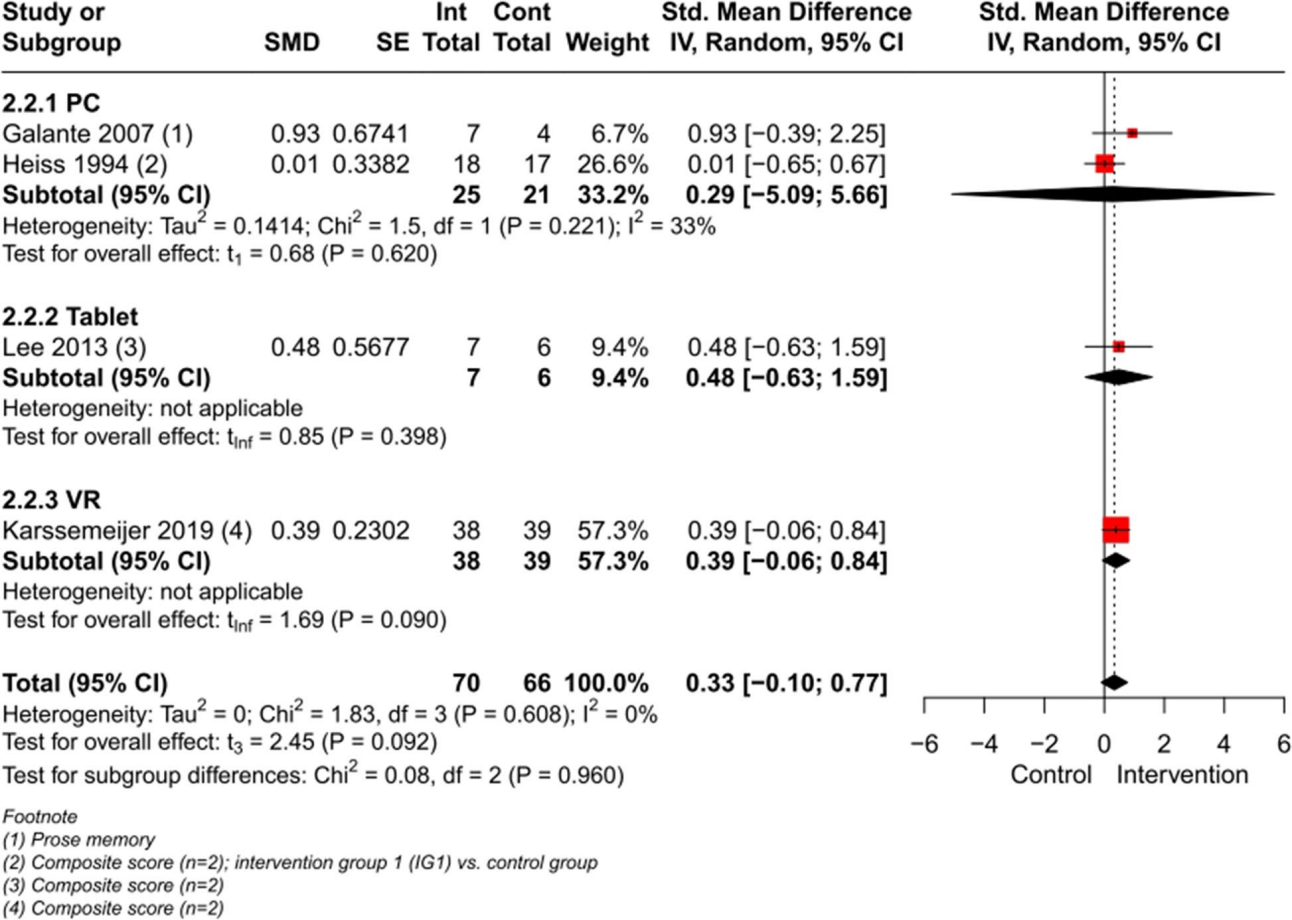
Meta-analysis of CCIs for people with dementia vs. control immediately post intervention on memory

A meta-analysis (Fig. 4) with a total of 7 studies involving 306 participants was conducted on the outcome memory function, showing a significant effect of CCIs vs. control immediately post intervention (SMD 1.13, large effect size, CI 95% [0.01, 2.25], I^2^ = 93%) (for composite scores computation see Figures 3–6 in Additional file 4). When excluding the AR-study [53] with a large SMD, the heterogeneity drops to I^2^ = 59% (SMD 0.64, medium effect size, CI 95% [0.11, 1.18]) (Figure 7 in Additional file 4).

**Fig. 8:**
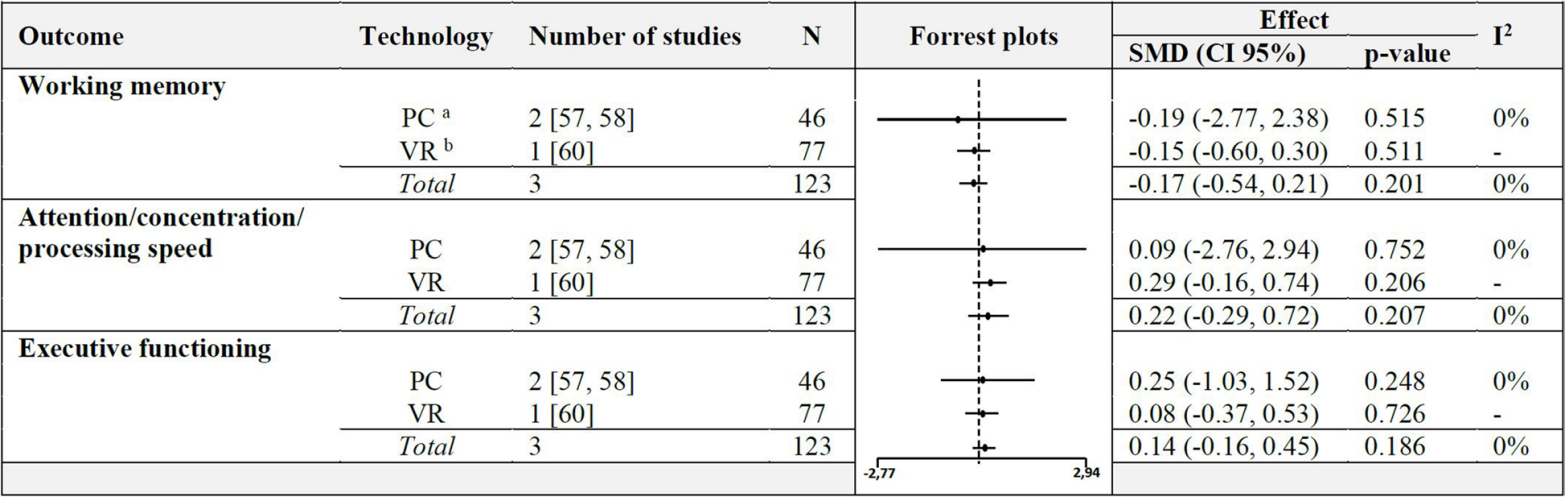
Meta-analyses with subgroups of CCIs on people with dementia vs. control immediately post intervention. ^a^ PC: personal computer. ^b^ VR: virtual reality

Most studies have been pooled for the subgroup PC (*n* = 5) showing a non-significant effect on memory function with a heterogeneity of I^2^ = 64%. Excluding the two studies [46, 57], with a non-computer-based CT as a control group, let the heterogeneity drop to I^2^ = 0%, resulting in a significant effect on memory function (SMD 0.89, large effect size, CI 95% [0.56, 1.21]) (Figure 8 in Additional file 4). A meta-analysis with those two excluded studies [46, 57] demonstrated a non-significant effect for the intervention group (SMD 0.36, CI 95% [-7.35, 8.08], I^2^ = 81%), but indicated an improvement for both, the CCIs and the non-computer-based CT (Figure 9 in Additional file 4). In one of those two studies [46], the intervention and control group had CT activities focusing on the memory domain, leading to increased memory function in both groups with a non-significant group difference. In Herrera et al. [57] the CCI was exclusively targeting on recognition, in contrast to the control intervention, resulting in beneficial significantly group differences for participants’ memory in the intervention group.

Excluding the AR-study [53] and both above-mentioned studies [46, 57] with the nearly same intervention in both, the intervention and control group, from the main meta-analysis, the significant effect remains (SMD 0.87, large effect size, CI 95% [0.70, 1.03]), but with an heterogeneity of I^2^ = 0% (Figure 10 in Additional file 4).

Meta-analyses on the other domain-specific cognitive outcomes such as working memory, attention/concentration/processing speed and executive functioning showed significant effects for people with MCI and applied CCIs vs. control immediately post intervention. The meta-analysis on the outcome language showed no beneficial effects for participants performing CCIs with a PC compared to control groups (Fig. 5, Figures 11–23 in Additional file 4).

No pooling of studies was possible for the outcome visuospatial/constructional abilities.

Three studies could not be included in any meta-analysis, because of either non-reported, or inappropriate data [51, 56, 59]. In one of these studies [51] working memory increased by participants in the PC-based intervention group (Wechsler Memory Scale 3^rd^ edition, spatial span, *p* = 0.003), but measurement for memory, executive functions, attention/concentration/processing speed, language and visuospatial/constructional abilities revealed no significant differences between the intervention and control group. In Li et al. [59], 2 out of 6 measurements on memory showed significant effects for participants using a PC-based CT (Addenbrooke’s Cognitive Examination Revised (ACER), memory *p* < 0.05; Auditory Verbal Learning Test, 5-min recall, *p* < 0.01). Furthermore one out of 3 measurements in executive functions (ACER, fluency, *p* < 0.01), one out of 5 on attention/concentration/processing speed (ACER, attention, *p* < 0.05) and one out of 2 for visuospatial/constructional abilities (Rey-Osterreith Complex Figure, copy, *p* < 0.05) were significant, while one measurement on language showed no significant effects. In the third study [56], one of three measurements for memory function showed significant improvements for participants with a CT on tablets as intervention (Word List Recall Test, *p* = 0.031).

Two studies [55, 58] that had the same CCIs in the intervention and control groups, with the only difference being the adjustability of difficulty levels in the intervention group, were pooled for meta-analyses on memory, working memory and executive functioning immediately after the intervention, but showed no significant benefits (Figures 24–29 in Additional file 4).

#### Domain-specific cognition (Months after Intervention)

A follow-up was conducted in two studies, in one of which Li et al. [59] found no significant group differences 12 months after the intervention, in contrast to the post-intervention evaluation, while the second study [57] found continuing significant differences for the intervention group after 6 months in memory (Doors recognition subtest, Set A, *p* < 0.05; BEM144, 12-word-list-recall test, total score, *p* < 0.05) and working memory (Digit Span, forward, *p* < 0.05).

Pooling the follow-up data of Hyer et al. [58] (3 months after the intervention) and Flak et al. [55] (4 months after the intervention), which had the same CCIs in the intervention and control group, only differing in the adjustability of difficulty levels for the intervention group, showed no significant effects (Figures 30–35 in Additional file 4).

### Effects of CCIs on cognition of people with dementia

#### Global Cognition (immediately after Intervention)

Four studies were pooled for a meta-analysis (Fig. 6) of CCIs vs. control immediately post intervention on global function in people with dementia, which demonstrated a non-significant effect (SMD 0.53, CI 95% [-1.08, 2.14]), with a heterogeneity of I^2^ = 86%. Excluding Cinar et al. [45], with a large SMD, let the heterogeneity drop to I^2^ = 0%, remaining in a non-significant effect (SMD 0.03, CI 95% [-0.91, 0.97]) (Figure 36 in Additional file 4).

One Study [69] could not be included in the meta-analysis, because of inappropriate data for pooling, but confirmed the non-significant effect, measured by the MMSE and the MoCA.

#### Global cognition (Months after Intervention)

Two studies using a PC [65] and tablet [47] for their intervention examined global cognition after a 3-month follow-up and were pooled for a meta-analysis, with a non-significant result (SMD -0.06, CI 95% [-4.40, 4.28], I^2^ = 0%) (Figures 37–38 in Additional file 4).

#### Domain-specific cognition (immediately after Intervention)

A meta-analysis with 4 studies (Fig. 7) demonstrated a non-significant effect, but a tendency of CCIs to increase memory functions in people with dementia (SMD 0.33, CI 95% [-0.10, 0.77], I^2^ = 0%) (for composite scores computation see Figures 39–41 in Additional file 4). Further meta-analyses (Fig. 8) on the outcomes working memory, attention/concentration/processing speed and executive functioning showed that participants, performing CCIs whether using a PC nor VR-technology had no beneficial effects compared to control groups (Figures 42–49 in Additional file 4).

Two studies could not be included in meta-analyses because of inappropriate reported [45, 69] data. Yu [69], confirmed the pooled results with non-significant group differences in working memory and executive functioning. In contrast, the other study of Cinar et al. [45] revealed significant improvements with the Cambridge Cognition CANTAB assessment for the intervention group in memory (DMS, percent correct, *p* = 0.001; DMS, percent correct, all delays, *p* = 0.01; PAL, total errors (adjusted), *p* = 0.001; PAL, total errors, 6 shapes adjusted, *p* = 0.02). The authors [45] also reported significant results on PRM and sub outcomes on DMS and SWM, but without clear descriptions of the specific outcome measures (e.g. latency). The RT of the CANTAB assessment showed a non-significant change between the groups.

#### Domain-specific cognition (Months after Intervention)

Meta-analyses with studies which conducted a follow-up after 3 months, showed no beneficial effects for memory, working memory, attention/concentration/processing speed, executive function and memory (Figures 50–59 in Additional file 4).

## Discussion

This systematic review and meta-analyses investigated whether individually performed CCIs have an impact on global and domain-specific cognition in community-dwelling people with SCD, MCI and dementia. CCIs were especially beneficial for people with MCI, revealing significant effects in memory, working memory, attention/concentration/processing speed and executive functioning, but no significant improvements in global cognition and language. Most of the overall outcomes showed a large effect size, but also a substantial or considerable heterogeneity, which is why the confidence in these results is limited. Pooled results of studies on people with dementia demonstrated no significant effects on cognition, but a tendency towards an increased memory function (SMD 0.33, CI 95% [-0.10, 0.77], I^2^ = 0%) was observed. While statistically not significant, with a current small effect size, this finding may be clinically significant, but more studies with larger samples are needed to investigate a possible statistical significance. Only one RCT [45] was identified investigating a web-based CT on a PC in people with SCD, which reported significant results on memory function for participants in the intervention group. Follow-up evaluations examining the long-term effect of such interventions were only conducted by a few studies [47, 55, 57–59, 65, 67], where pooled estimates showed no significant effects for people with MCI or dementia. Of the studies that could not have been pooled, only one [57] showed continuing significant improvements for MCI-patients in the intervention group at 6 months after intervention in memory and working memory functions.

No meta-analyses on the condition of SCD could be conducted in our systematic review, as only one study [45] met our eligible criteria. Two systematic reviews and meta-analysis [70, 71] on SCD demonstrated a growing research interest and indicated beneficial impacts of cognitive exercises on cognition of people with SCD. One [70] of those reviews included the RCT of Pereira-Morales et al. [72] investigating a web-based CT on cognition. Primary findings of this study [72] showed at least a significant improvement for the CCI on an memory outcome, as it was also measured in Cinar et al. [45], the study included in our review. However, diagnostic criteria of SCD for participants in Pereira-Morales et al. [72] were not clearly described and hence it was not considered for the inclusion in our review. Therefore, it is demonstrated that more high-quality research on CCIs’ effectiveness, applying standard and emerging technologies with standardized SCD criteria, is needed. This is important for demonstrating whether CCIs at this early stage present a promising option for dementia prevention. Furthermore, the necessity for rising awareness about SCD in general must be also considered earlier, as the Behavioral Risk Factor Surveillance System survey, which asked people for self-perceived memory loss, found that in 11% of affected persons only 46% of these consulted health care professionals [2, 8, 73].

According to our findings, people with MCI benefit from CCIs the most. Zhang et al. [21] and Hill et al. [22] evaluated computer-based CT on people with MCI in their systematic reviews and corroborate our results, as most of their pooled study results showed significant improvements in different cognitive domains (e.g. memory, working memory) for participants in the intervention group. In contrast to our findings, meta-analyses on the global cognition revealed significant effects in both reviews [21, 22]. The reason for the differing findings could be that Zhang et al. [21] and Hill et al. [22] had defined other eligible criteria, as they had not considered the training format (individual or group trainings) or the setting (e.g. nursing homes) nor included emerging technologies like VR [21] and AR [21, 22]. Although we included emerging technologies in our review, PCs were the most common technology used in MCI-studies. Pooled VR-studies for people with MCI (*n* = 3), however, already showed a significant effect on attention/concentration/processing speed. In this regard a significant effect on executive functioning was identified in the systematic review of Wu et al. [19], who evaluated VR-based cognitive interventions in people with MCI. In contrast to our review, Wu et al. [19] identified another auspicious finding, namely the effectiveness of such interventions in global functions demonstrated by a meta-analysis with 13 RCTs. Wu et al. [19] included studies utilizing VR along with traditional rehabilitative treatment, limiting the interpretation of pooled effects, which was not the case in our review.

The aforementioned systematic review of Hill et al. [22] did not conduct meta-analyses on people with MCI only, but also separately on people with dementia. In contrast to the non-effective findings in our review the authors [22] reported beneficial evidence with pooled studies on overall cognitive outcomes and visuospatial skills in people with dementia performing computer-based CT. A further meta-analysis from Garcia-Carsal et al. [17] demonstrated a significant effect of CCIs on global cognition of people with dementia and additionally revealed that CCIs seemed to be more beneficial compared to non-computer-based CT. However, Garcia-Carsal et al. [17] included not only RCTs but also heterogeneous study designs such as case control studies in their meta-analyses, which have a lower level of evidence compared to RCTs [74].

Only one study investigated a CCI with an emerging technology, namely non-immersive VR by people with dementia [67], although such technologies seem to be very promising in terms of their cognitive approaches to CR and CS. In the context of CR, technologies like AR, VR and MR could be used for carrying out individual (I)ADL-trainings (e.g. making tea) or even be integrated in everyday live to independently stay at home as long as possible [75]. However, an increased cognition did not lead concurrently to an improvement in (I)ADL, which the results of Hill et al. [22] and Garcia et al. [17] justified with significant effects regarding cognition but not for the outcome of (I)ADL. In this regard, especially increased executive functions are associated with an improvement in (I)ADL performance [76, 77], which raises the need for more research on CR and emerging technologies that focus on this cognitive domain.

Furthermore, CS, which is not represented in the present review, could be well applied, for example by practicing reminiscence therapy by integrating scenarios from individuals’ biography [75]. Reminiscence therapy on persons with dementia using a tablet was already investigated by a recent RCT [78] (out of our search time frame), showing no significant results on cognition. With emerging technologies, new possibilities open up for people with dementia to immerse themselves in the past, stimulating their cognition with the help of a virtual environment [75]. However, in terms of such technologies and different cognitive approaches, RCTs are needed to verify their effectiveness. It was also observed that more studies investigating emerging technologies for MCI than on dementia were included in our review. This may be due to the greater resources required for conducting studies on persons with dementia (e.g. supervision, time for assessments), because of disease-related symptoms [79].

We identified two additional recent RCTs that were published after our literature search and therefore were not included in our analysis. Duff et al. [80] investigated CCIs in people with MCI. The authors [80] compared an intervention group utilizing selected exercises from a known computerized cognitive training program on a PC, which already showed beneficial effects on cognition in previous literature, with a control group using computerized games from the same program, without clear beneficial findings. The primary outcome, a composite score named auditory memory/attention significantly increased for participants in the active control group. Despite the similarity of the compared interventions, the composite score does not match our domain classification. Furthermore, global cognition did not increase significantly in the intervention group [80], corresponding with our findings. The second identified RCT [81] evaluated a CCI also on a PC compared to a control group which received only educational material during the pretest on people with dementia. Results on objective cognition revealed no significant impact corresponding with the meta-analysis in our review, whereas subjective cognition evaluated by participants’ relatives showed significant effects for participants in the intervention group [81]. However, proxy-measurements on subjective cognition were not considered in our systematic review.

Overall, most interventions were conducted in a lab setting under optimal conditions (e.g. constant technical support), as it is important to investigate the effectiveness more realistically at the participants’ home, giving them the opportunity to practice any time [23]. There is a particular need for research for persons with dementia, as only one [45] of six studies was conducted at the home of a participant. Since people with dementia are usually limited in (I)ADLs, the need for a transport to visit the training lab can be challenging and could cause additional burden on their caregivers [2].

For the application of CCIs at home, the acceptance and usability of the interventions are particularly necessary to enable an easy use and regular training performances [82, 83], as the training intensity appears to be important for effectiveness [13]. Usability research for CCIs, specifically on the older population, is still lacking [82, 83].

Furthermore, the implementation of emerging technologies in the home setting may be hindered by the current high cost of the needed products (e.g. head-mounted display for immersive virtual reality). However, research on the use of smartphones is already underway that may open the option of creating a virtual environment for computer-based cognitive interventions at home [19].

### Strengths and limitations

A strength of this systematic review was the comprehensive literature search and the well-structured selection process to identify relevant studies and to minimize a publication bias. Despite the effort to avoid a publication bias, it cannot be excluded, as a screening of study registries had not taken place [31]. It might be possible that technology companies did not publish studies because of non-significant results.

The authors had defined clear eligible criteria for this systematic review to show effects for specific subgroups, however, it was recognized that some studies did not describe their eligibility criteria, such as the setting or diagnostic criteria of participants in a manner that was sufficient to fit our definitions and for further inclusion in this review. While the authors were contacted for further information, insufficient reporting may have resulted in missed inclusion of potential studies.

Furthermore, our review focused on a broad outcome containing global and domain-specific cognition, for three different target groups measured immediately after post-intervention and at follow-up, which resulted in more than 120 different outcome (sub)measurements. In some cases, measurements were reported with minor differences in naming or with insufficient detail of which instrument was used. Measurements of this kind were excluded in cases of serious doubts. Most studies used multiple measures for different kind of cognitive domains, which constituted a challenge in classifying those in one of our pre-defined cognitive domains and furthermore made a calculation of composite scores [44] necessary for most pooled studies. For that reason, different (un)established instruments were summarized for calculating such a composite score, which could not always result in an optimal assessment for a given cognitive domain.

Finally, for the conduction of our meta-analyses we applied the random-effects model, because of the variability in the participants, interventions and outcomes, as it cannot be assumed that the true effect sizes are all the same or rather fix [30, 39, 84]. Although, in practice, the random-effects model predominates [84], it is not entirely controversial, especially for smaller studies, as this model may have a poor precision with a small number of studies in a meta-analysis [39]. However, the Hartung-Knapp adjustment addresses the issue of small number of studies [30].

## Conclusions

The findings of this systematic-review and meta-analyses demonstrated that individually performed CCIs had beneficial effects on domain-specific cognition in community-dwelling people with MCI, but no significant effects on people with dementia. However, for people with dementia, a tendency towards an increased memory function could be observed. In particular, for people with MCI, most meta-analyses revealed a substantial or considerable heterogeneity, which is why the confidence in these results is limited. In terms of SCD, only one study was identified that demonstrated significant results on memory functions for participants in the intervention group using a web-based CT on a PC. In general, most CCIs were conducted with PCs, followed by tablets, VR, AR, and MR.

When considering CCIs, the maxim “the earlier, the better” summarizes our results best, as the findings suggested that CCIs are already a valuable intervention for people with MCI to preserve/improve cognition, but more research on SCD is needed. CCIs therefore have the potential to complement standard (non-) pharmacological treatment as they open a low threshold offering in a stigmatized area. Apart from the underlying condition, the decision to provide such trainings should additionally be made with consideration for the personal values, preferences, and available resources of the people concerned. In this context, it would be particularly important to investigate CCIs not only in well-prepared laboratory settings, as was the case in most of the included studies, but more realistically in people's homes to provide easy access and the opportunity to conduct the training at any time, since a higher training intensity appears to increase the chance for effectiveness. However, a prerequisite for regular practice is the user-friendliness of CCIs, which must be evaluated and considered in the context of the needs people have, in respect to technologies and the home setting. Furthermore, future studies should focus more on emerging technologies (e.g. VR) where people could interact with its environment, as these technologies are predicted as important game changers in the field of dementia prevention and treatment.

Finally, the development of a set of essential cognitive outcomes and instruments for consistent use in RCTs is recommended, as well as to report such findings comprehensively and transparently, making the pooling of evidence easier and more precise for future decisions.

## Supplementary Information

**Additional file 1.** Search strategies of all databases.

**Additional file 2.** Characteristics of the included RCTs.

**Additional file 3.** Outcomes of the systematic review and the respective assigned instruments identified in the studies.

**Additional file 4.** Meta-analyses and composite scores.

## Data Availability

The data supporting the findings of this systematic review are included within the article and its additional files.

## Abbreviations

ADL: Activities of daily living
ACER: Addenbrooke’s Cognitive Examination Revised
AR: Augmented reality
CCIs: Computer-based cognitive interventions
CG: Control group
CR: Cognitive rehabilitation
CS: Cognitive stimulation
CT: Cognitive training
DMS: Delayed matching sample
IADL: Instrumental activities of daily living
IG: Intervention group
MCI: Mild cognitive impairment
MMSE: Mini Mental State Examination
MoCA: Montreal Cognitive Assessment
MR: Mixed reality
PAL: Paired associated learning
PRM: Pattern recognition memory
RCT: Randomized controlled trial
RT: Reaction time
SCD: Subjective cognitive decline
SWM: Spatial-working memory
SMD: Standardized mean difference
VR: Virtual reality
