# Supplementary material for: Effectiveness of computer-based interventions for community-dwelling people with cognitive decline: a systematic review with meta-analyses
Source: BMC Geriatr. 2023 Apr 12;23:229. doi: 10.1186/s12877-023-03941-y (PMC10091663; doi:10.1186/s12877-023-03941-y)
Supplement: Supplementary file 1 — Additional file 1. Search strategies of all databases. [file 12877_2023_3941_MOESM1_ESM.docx]

**Additional file 1**

**Table 1: Search strategies of all databases**

| **Queries** | **Hits** |
| --- | --- |
| **Medline via Pubmed** | |
| ((dementia [MH] OR alzheimer disease [MH] OR dementia, vascular [MH] OR cognitive impairment OR MCI OR cognition disorders [MH] OR neurocognitive disorders [MH] OR cognitive dysfunction [MH])) AND (computerized cognitive training OR computer-based cognitive training OR computer-based memory training OR touchscreen technology OR tablet* OR serious game* OR brain training game* OR virtual reality [MH] OR augmented reality [MH] OR game console* OR Wii OR video gam* OR computer gam* OR web-based training OR internet-based training OR computer training OR telerehabilitation [MH]) Sort by: Best Match Filters: English; German | 2.587 |
| **CINAHL via Ovid** | |
| ( (MH “dementia+”) OR (MH "Alzheimer's Disease") OR (MH "Dementia, Vascular") OR cognitive impairment OR MCI OR (MH "Cognition Disorders") OR neurocognitive disorders OR cognitive dysfunction ) AND ( computerized cognitive training OR computer-based cognitive training OR computer-based memory training OR web-based training OR internet-based training OR computer training OR touchscreen technology OR tablet* OR serious game* OR brain training game* OR (MH "Virtual Reality") OR (MH "Augmented Reality") OR game console* OR Wii OR video gam* OR computer gam* OR (MH "Telerehabilitation") ) Limiters - Language: English, German | 1.047 |
| **Embase via Ovid** | |
| ((dementia or alzheimer disease or dementia, vascular or cognitive impairment or MCI or cognition disorders or neurocognitive disorders or cognitive dysfunction) and (computerized cognitive training or computer-based cognitive training or computer-based memory training or web-based training or internet-based training or computer training or touchscreen technology or tablet* or serious game* or brain training game* or virtual reality or augmented reality or game console* or Wii or video gam* or computer gam* or telerehabilitation)).af  limit 2 to (english or german) | 1.766 |
| **Cochrane RCT via Ovid** | |
| ((dementia or alzheimer disease or dementia, vascular or cognitive impairment or MCI or cognition disorders or neurocognitive disorders or cognitive dysfunction) and (computerized cognitive training or computer-based cognitive training or computer-based memory training or web-based training or internet-based training or computer training or touchscreen technology or tablet* or serious game* or brain training game* or virtual reality or augmented reality or game console* or Wii or video gam* or computer gam* or telerehabilitation)).af. | 1.071 |
| **IEEE** | |
| (("All Metadata":dementia OR alzheimer disease OR vascular dementia OR cognitive impairment OR MCI OR cognition disorder OR neurocognitive disorders) AND "All Metadata":"cognitive training" OR "computerized cognitive training" OR "computer-based cognitive training" OR "memory training" OR "serious game*" OR "brain training") | 2.731 |
| **Web of Science** | |
| TS=(dementia  OR alzheimer disease  OR dementia, vascular  OR cognitive impairment  OR MCI  OR cognition disorders  OR neurocognitive disorders  OR cognitive dysfunction)  AND TS=(computerized cognitive training  OR computer-based cognitive training  OR computer-based memory training  OR web-based training  OR internet-based training  OR computer training  OR touchscreen technology  OR tablet*  OR serious game*  OR brain training game*  OR virtual reality  OR augmented reality  OR game console*  OR Wii  OR video gam*  OR computer gam*  OR telerehabilitation)  Refined by: LANGUAGES: ( ENGLISH OR GERMAN )  Timespan: All years. Databases:  WOS, KJD, MEDLINE, RSCI, SCIELO.  Search language=Auto | 6.177 |
| **Scopus** | |
| ( TITLE-ABS-KEY ( *"dementia"* )  OR  TITLE-ABS-KEY ( *"alzheimer disease"* )  OR  TITLE-ABS-KEY ( *"dementia, vascular"* )  OR  TITLE-ABS-KEY ( *"cognitive impairment"* )  OR  TITLE-ABS-KEY ( *"MCI"* )  OR  TITLE-ABS-KEY ( *"cognition disorders"* )  OR  TITLE-ABS-KEY ( *"neurocognitive disorders"* )  OR  TITLE-ABS-KEY ( *"cognitive dysfunction"* ) )  AND  ( TITLE-ABS-KEY ( *"computerized cognitive training"* )  OR  TITLE-ABS-KEY ( *"computer-based cognitive training"* )  OR  TITLE-ABS-KEY ( *"computer-based memory training"* )  OR  TITLE-ABS-KEY ( *"web-based training"* )  OR  TITLE-ABS-KEY ( *"internet-based training"* )  OR  TITLE-ABS-KEY ( *"computer training"* )  OR  TITLE-ABS-KEY ( *"touchscreen technology"* )  OR  TITLE-ABS-KEY ( *"tablet*"* )  OR  TITLE-ABS-KEY ( *"serious game*"* )  OR  TITLE-ABS-KEY ( *"brain training game*"* )  OR  TITLE-ABS-KEY ( *"virtual reality"* )  OR  TITLE-ABS-KEY ( *"augmented reality"* )  OR  TITLE-ABS-KEY ( *"game console*"* )  OR  TITLE-ABS-KEY ( *"Wii"* )  OR  TITLE-ABS-KEY ( *"video gam*"* )  OR  TITLE-ABS-KEY ( *"computer gam*"* )  OR  TITLE-ABS-KEY ( *"telerehabilitation"* ) )  AND  ( LIMIT-TO ( LANGUAGE ,  *"English"* )  OR  LIMIT-TO ( LANGUAGE ,  *"German"* ) ) | 2.189 |
| **PsycInfo via Ovid** | |
| ((dementia or alzheimer's disease or vascular dementia or cognitive impairment or MCI or cognition disorders or neurocognitive disorders or cognitive dysfunction) and (computerized cognitive training or computer-based cognitive training or computer-based memory training or web-based training or internet-based training or computer training or touchscreen technology or tablet* or serious game* or brain training game* or virtual reality or augmented reality or game console* or Wii or video gam* or computer gam* or telerehabilitation)).mp. [mp=title, abstract, heading word, table of contents, key concepts, original title, tests & measures, mesh] | 700 |
| **Google Scholar (the first 100 hits)** | |
| (dementia OR alzheimer's disease OR vascular dementia OR cognitive impairment OR MCI OR cognition disorder OR neurocognitive disorder) AND (computerized cognitive training OR computer-based cognitive training OR serious game OR memory training) |  |
| **Research Gate (the first 100 hits)** | |
| dementia and computerized cognitive training or computer-based cognitive training |  |
