# Supplementary material for: Effectiveness of computer-based interventions for community-dwelling people with cognitive decline: a systematic review with meta-analyses
Source: BMC Geriatr. 2023 Apr 12;23:229. doi: 10.1186/s12877-023-03941-y (PMC10091663; doi:10.1186/s12877-023-03941-y)
Supplement: Supplementary file 2 — Additional file 2. Characteristics of the included RCTs. [file 12877_2023_3941_MOESM2_ESM.docx]

**Additional file 2**

**Table 1: Characteristics of the included RCTs**

| **Authors; Country** | **Sample** | | | | | | **Intervention** | | | | | | | **Follow-up (months)** |
| --- | --- | --- | --- | --- | --- | --- | --- | --- | --- | --- | --- | --- | --- | --- |
|  | **Type of cognitive decline** | **Sample size (IG ^a^/CG ^b^)** | **Age x̅** | **Female %** | **Global cognition x̅** | **Years of education x̅** | **TECH ^c^** | **Description** | **Cognitive approach** | **Duration in weeks** | **Sessions/week, (minutes/session)** | **Total number of sessions** | **Setting** |  |
| Cinar et al. 2020; [45] Turkey | SCD ^d^ | 60 (30/30) | 67.4 ^e^ | 61.7 | 23.8 MoCA ^f^ | 12.4 ^e^ | PC ^g^ | IG: BEYNEX, web-based program included 3 different games and physical exercises (per video)  *Addressed cognitive domains:* nr | CT ^h^ | 12 (or at least 1200 min of training) | about 7 (15-20 minutes) | 60-80 | Home | - |
|  |  |  |  |  |  |  |  | CG: No intervention |  |  |  |  |  |  |
| Amjad et al. 2019; [50] Pakistan | MCI ^i^ not specified | 38 (18/20) | nr ^j^ | nr | nr | nr | VR ^k^ | IG: VR-based Xbox 360 Kinect platform with commercially available games (‘‘Body and Brain Exercises’’ by Dr. Kawashima)  *Addressed cognitive domains:* logic, physical, memory, reflexes, math | CT | 6 | 5 (20-30 minutes) | 30 | Lab | - |
|  |  |  |  |  |  |  |  | CG: Motion and stretching exercises of upper and lower limbs |  |  |  |  |  |  |
| Barnes et al. 2009; [51] USA | MCI all types | 47 (22/25) | 74.0 | 40.0 | 86.5 RBANS ^l^ | 17.0 | PC | IG: Computer-based, cognitive training program developed by Post Science Corporation (San Francisco, CA)  *Addressed cognitive domains:* processing speed, accuracy in the auditory cortex | CT | 6 | 5 (100 minutes) | 30 | Home | - |
|  |  |  |  |  |  |  |  | CG: Three types of computer-based activities (audio books, online newspapers, visuospatial-oriented computer game) |  |  |  |  |  |  |
| Damirchi et al.  2018; [52] Iran | MCI not specified | 44 (11/11/13/9) | 68.4 ^e^ | 100 | 23.4 MMSE ^m^ | 3.2 ^e^ | PC | IG1: Program titled “Modified My Better Mind” with 4 different games  *Addressed cognitive domains:* visual attention, visual working memory (visuospatial and verbal), processing speed, anectodal knowledge, verbal memory, reasoning, spatial executive processing, visual-spatial skills | CT | 8 | 3 (30 minutes in weeks 1-6;  60 minutes in 7^th^ and 8^th^ week) | 24 | Lab | - |
|  |  |  |  |  |  |  |  | IG2: Physical activity group |  |  |  |  |  |  |
|  |  |  |  |  |  |  |  | IG3: Program “Modified My Better Mind” combined with physical group activities |  |  |  |  |  |  |
|  |  |  |  |  |  |  |  | CG1: Waiting list group |  |  |  |  |  |  |
| Dimitriadis et al. [53] 2016; Greece | MCI not specified | 158 (53/50/55) | 69.1 ^e^ | 73.7 | 24.9 MMSE ^e^ | 7.3 ^e^ | AR ^n^ | IG: Novel Serious game (Mnemonic Strategy training) with a hide-and-seek exercise and a dual task condition using the hands and arms  *Addressed cognitive domains:* working and spatial memory, executive function (eg, volition, self-awareness, planning, inhabitation of dominant response and external distraction during response control and dual-task coordination) | CT | 10 | 4-5 (90 minutes) | 40-50 | Home | - |
|  |  |  |  |  |  |  |  | CG1: Watching YouTube documentaries |  |  |  |  |  |  |
|  |  |  |  |  |  |  |  | CG2: Waiting list group |  |  |  |  |  |  |
| Finn et al. 2011; [54] Australia | MCI amnestic multiple domain | 16 (8/8) | 72.7 ^e^ | 50.0 | 28.0 MMSE ^e^ | 12.6 ^e^ | PC | IG: Training software by Lumosity Inc, which consisted of 30 training sessions each containing four or five cognitive exercises  *Addressed cognitive domains:* nominally attention, processing speed, visual memory, cognitive control | CT | average of 11.4^o^ | 4-5 (nr) | 30 | Home | - |
|  |  |  |  |  |  |  |  | CG: Waiting list group |  |  |  |  |  |  |
| Flak et al. 2019; [55] Norway | MCI all types | 69 (35/34) ^p^ | 66.0 ^e^ | 33.3 | nr | 13.2 ^e^ | PC | IG: Cogmed ^®3^, adaptive (ie. available difficulty levels) computerized training program  *Addressed cognitive domains:* visuospatial working memory, a combination of verbal and visual working memory | CT | 5 | 5 (30-40 minutes) | 20-25 | Home | 4 |
|  |  |  |  |  |  |  |  | CG: Cogmed ^®3^, non-adaptive (fixed low level of difficulty) computerized training program |  |  |  |  |  |  |
| Hagovska et al. 2017; [46] Slovakia | MCI not specified | 60 (30/30) | 68.0 ^e^ | 51.7 | 25.3 MMSE ^e^ | Education level (%):  Secondary 85.0  University 15.0 | PC | IG: CogniPlus program that involved activities that are similar to everyday activities  *Addressed cognitive domains:* attention, working memory, long-term memory, planning of everyday activities, visual-motor abilities | CR ^q^ | 10 | 2 (30 minutes) | 20 | Lab | - |
|  |  |  |  |  |  |  |  | CG: Group cognitive training program |  |  |  |  |  |  |
| Han et al. 2017; [56] South Korea | MCI all types | 43 (43/42) ^r^ | 74.0 | 46.5 | 25.1 MMSE | 13.2 | Tablet | IG: Ubiquitous Spaced Retrieval-based Memory Advancement and Rehabilitation Training (USMART)  *Addressed cognitive domains:* memory | CT | 4 | 2 (30 minutes) | 8 | Lab | - |
|  |  |  |  |  |  |  |  | CG: Usual treatment |  |  |  |  |  |  |
| Herrera et al. 2012; [57] France | MCI amnestic multiple domain | 22 (11/11) | 76.6 ^e^ | 50.0 | 27.3 MMSE ^e^ | Education level (%) ^e^:  Primary school 45.5  Secondary school 40.5  more than secondary school 14.0 | PC | IG: Cognitive training includes memory and attentional tasks.  *Addressed cognitive domains:* memory, attention (visual focused attention, visuospatial focused attention, divided attention) | CT | 12 | 2 (60 minutes) | 24 | Lab | 6 |
|  |  |  |  |  |  |  |  | CG: Cognitive activities (eg. finding names of countries read a text and then answer questions) |  |  |  |  |  |  |
| Hyer et al. 2016; [58] USA | MCI amnestic and non-amnestic | 68 (34/34) | 75.2 ^e^ | 52.9 | 84.5 RBANS ^e^ | Education level (%):  High school or more 68.0 | PC | IG: Cogmed ^QM©^, adaptive (ie, available difficulty levels) computerized training program  *Addressed cognitive domains:* working memory (temporary storage and manipulation of sequential visuospatial and/or verbal information) | CT | 5-7 | about 5 (40 minutes) | 25 | Lab or Home | 3 |
|  |  |  |  |  |  |  |  | CG: Cogmed, non-adaptive (fixed low level of difficulty) computerized training program (ie. Sham). |  |  |  |  |  |  |
| Li et al. 2019; [59] China | MCI amnestic (due to AD ^s^) | 141 (78/63) | 70.4 | nr | 28.0 MMSE ^e^ | 13.7 ^e^ | PC | IG: Training comprised 8 tasks *Addressed cognitive domains:* visual working memory, 30-second memory, episodic memory, seed of calculation, visual search, alertness, mental rotation, images re-arrangement task | CT | 24 | 3-4 (about 40 minutes) | 72-69 | Home | 12 |
|  |  |  |  |  |  |  |  | CG: No intervention |  |  |  |  |  |  |
| Lin et al. 2016; [60] USA | MCI amnestic multiple domain  (due to AD) | 21 (10/11) | 72.0 ^e^ | 47.6 | 25.0 MoCA ^e^ | Education level (%):  High school or lower 28.6 | PC | IG: Vision-based speed of processing (VSOP) training used the INSIGHT online program (Posit Science) which included five training tasks.  *Addressed cognitive domains:* visual processing speed, attention | CT | 6 | 4 (60 minutes) | 24 | Home | - |
|  |  |  |  |  |  |  |  | CG: Mental leisure activities like online crossword, Sudoku, and solitaire games |  |  |  |  |  |  |
| Nousia et al. 2019; [48] Greece | MCI all types | 46 (25/21) | 71.6 ^e^ | 76.1 | 21.8 MoCA ^e^ | 8.7 ^e^ | PC | IG: RehaCom Cognitive Therapy Software  *Addressed cognitive domains:* episodic and delayed memory, verbal memory, attention, processing speed, executive function. | CR | 15 | 2 (60 minutes) | 30 | Lab | - |
|  |  |  |  |  |  |  |  | CG: Usual treatment |  |  |  |  |  |  |
| Park et al. 2019; [49] South Korea | MCI single and multiple domain | 21 (10/11) | 71.9 ^e^ | 81.0 | 26.7 MMSE ^e^ | 7.1 ^e^ | Mixed Reality | IG: MR-based cognitive training system (Mixed Reality System for Health), which combines augmented and virtual reality.  *Addressed cognitive domains:* selective attention, visual and verbal working memory, executive function, (including sequencing, planning, and problem solving), calculation. | CR | 6 | 3 (30 minutes) | 18 | Lab | - |
|  |  |  |  |  |  |  |  | CG: Computer-assisted cognitive training system COMOG (Maxmedica), which provides 10 training activities  *Addressed cognitive domains:* visual and auditory processing, selective attention, working memory, emotional attention |  |  |  |  |  |  |
| Park et al. 2020; [61] South Korea | MCI amnestic | 21 (10/11) | 70.6 ^e^ | 66.7 | 25.7 MMSE ^e^ | 7.6 ^e^ | VR | IG: Culture-based VR-training program stimulating cognitive function with 6 games  *Addressed cognitive domains:* attention, processing speed, executive function, memory | CT | 12 | 2 (30 minutes) | 24 | Lab | - |
|  |  |  |  |  |  |  |  | CG: Waiting list group |  |  |  |  |  |  |
| Rosen et al. 2011; [62] USA | MCI amnestic | 12 (6/6) | 74.3 ^e^ | nr | 28.6 MMSE ^e^ | 17.5 ^e^ | PC | IG: Computer-based, cognitive training program developed by Post Science Corporation (San Francisco, CA), with 7 exercises  *Addressed cognitive domain:* processing speed | CT | Average of 8 ^t^ | 5 (100 minutes) | 24 | Home | - |
|  |  |  |  |  |  |  |  | CG: Three types of computer-based activities (audio books, online newspapers, visuospatial-oriented computer game) |  |  |  |  |  |  |
| Savulich et al. 2017; [63] United Kingdom | MCI amnestic (due to AD) | 42 (21/21) | 76.1 ^e^ | 40.5 | 26.7 MMSE ^e^ | Age left education, x̅ ^e^: 15.95 | Tablet PC | IG: Memory game “Game Show”, with the intention to motivate  *Addressed cognitive domain:* episodic memory | CT | 4 | nr (60 minutes) | 8 | Lab | - |
|  |  |  |  |  |  |  |  | CG: No intervention |  |  |  |  |  |  |
| Thapa et al. 2020; [64] South Korea | MCI not specified | 68 (34/34) ^u^ | 72.5 ^e^ | 76.5 | 26.2 MMSE ^e^ | 8.9 ^e^ | VR | IG: 1) Training consists of 4 games developed by SY Innotech Inc.  *Addressed cognitive domain:* attention, memory, processing speed (Information on the right refers to Part 1 only)  2) Educational program on general health care | CT | 8 | 3 (100 minutes) | 24 | Lab | - |
|  |  |  |  |  |  |  |  | CG: Educational program on general health care |  |  |  |  |  |  |
| Cinar et al. 2020; [45] Turkey | Dementia AD | 60 (30/30) | 72.6 ^e^ | 50 | 20.0 MoCA ^e^ | 9.7 ^e^ | PC | IG: BEYNEX, web-based program included 3 different games and physical exercises (per video)  *Addressed cognitive domains:* nr | CT | 12 (or at least 1200 min of training) | about 7  (15-20 minutes) | 60-80 | Home | - |
|  |  |  |  |  |  |  |  | CG: No intervention |  |  |  |  |  |  |
| Galante et al. 2007; [65] Italy | Dementia AD | 12 (7/5) ^v^ | 76.0 | nr | 23.0 MMSE ^e^ | 6.3 | PC | IG: Selected tasks from Software Training NeuroPsicologico (TNP)  *Addressed cognitive domains:* memory, language, perception, attention, spatial cognition | CT | 4 | 3 (60 minutes) | 12 | Lab | 3;  9 |
|  |  |  |  |  |  |  |  | CG: Semi-structured interviews on current affairs and events relevant to participants’ lives |  |  |  |  |  |  |
| Heiss et al. 1994; [66] Germany | Dementia AD | 70 (18/17/18/17) | 66.3 | 45.7 | 20.8 MMSE ^e^ | nr | PC | IG1: Computer-based cognitive training program  *Addressed cognitive domains:* memory, perceptual or motor tasks | CT | 24 | 2 (60 minutes) | 48 | Lab | - |
|  |  |  |  |  |  |  |  | IG2 ^w^: Computer-based cognitive training program in combination with oral pyritinol 600 mg twice a day |  |  |  |  |  |  |
|  |  |  |  |  |  |  |  | IG3 ^w^: Computer-based cognitive training program in combination with oral phosphatidylserine 200 mg twice a day |  |  |  |  |  |  |
|  |  |  |  |  |  |  |  | CG: Social support through conversations about participants’ personal problems and how they managed daily life |  |  |  |  |  |  |
| Karssemeijer et al. 2019; [67, 68] The Netherlands | Dementia all types | 115 (38/38/39) | 79.9 | 46.1 | 22.4 MMSE | Education level (%):  Primary school education or lower 16.5  Secondary education or vocational training 58.3  Higher education 25.2 | VR | IG1: Training consisted of a combined cognitive– aerobic bicycle training developed by Bike Labyrinth ([www.bikelabyrinth.com](http://www.bikelabyrinth.com)), by which a stationary bike was connected to a video screen  *Addressed cognitive domains:* response inhibition, task switching, processing speed | CT | 12 | 3 (30-40 minutes) | 36 | Lab | 3 |
|  |  |  |  |  |  |  |  | IG2: Single aerobic exercise group consisted of cycling on a stationary bike without a video screen |  |  |  |  |  |  |
|  |  |  |  |  |  |  |  | CG: Active control group performed relaxation and flexibility exercises |  |  |  |  |  |  |
| Lee et al. 2013; [47] China | Dementia AD | 19 (7/6/6) | 77.7 | 68.4 | 16.6 MMSE ^e^ | Education level (%):  0 years 31.6  <2 years 15.8  3-6 years 26.3  Secondary21.0  University 0.05 | Tablet | IG1: Computer-assisted errorless learning program (CELP)  *Addressed cognitive domains:* sensory memory (visual and auditory), working memory, prospective memory, memory strategies for using mnemonics (eg, chunking, organization, and categorization), learning principles, name/face association, advanced memory training on application of strategies to train task in activities of daily living | CR | 6 | 2 (12-30 minutes) | 12 | Lab | 3 |
|  |  |  |  |  |  |  |  | IG2: Therapist-led training program (TELP) (not computer-based)  The content and structure of both programs CELP and TELP were similar, except for the mode of delivery. |  |  |  |  |  |  |
|  |  |  |  |  |  |  |  | CG: Waiting-list control group, which received general cognitively challenging activities to perform, such as card sorting |  |  |  |  |  |  |
| Yu et al. 2015; [69] China | Dementia not specified | 32 (16/16) | 83.0 | 69.0 | 16.6 MMSE | nr | Tablet | IG: Computer-assisted video game training, with 4 games  *Addressed cognitive domains:* working memory, attention | CT | 4-8 | 1-2 (30 minutes) | 8 | Lab | - |
|  |  |  |  |  |  |  |  | CG: Conventional cognitive training activities in which the training elements were matched with those in the computer-assisted videogame training |  |  |  |  |  |  |
| ^a^ IG: intervention group ^b^ CG: control group ^c^ TECH: Technology ^d^ SCD: subjective cognitive decline ^e^ marginal mean ^f^ MoCA: Montreal Cognitive Assessment (score 0-30, the more the better) ^g^ PC: personal computer  ^h^ CT: cognitive training ^i^ MCI: mild cognitive impairment ^j^ nr: not reported ^k^ VR: virtual reality ^l^ RBANS: Repeatable Battery for the Assessment of Neuropsychological Status (score 0-100, the more the better) ^m^ MMSE: Mini Mental State Examination (score 0-30, the more the better) ^n^ AR: augmented reality ^o^ The authors anticipated 6–10 weeks. Participants completed at least 80% of the sessions. ^p^ 68 (34/34) participants were included in the final analysis of the respectively study ^q^ Cross-over randomized controlled trial  ^r^ CR: cognitive rehabilitation ^s^ AD: Alzheimers’ disease  ^t^ Participants had to use the program until either achievement of asymptotic performance levels over a several day period or completion of 80% of the training material in a given exercise ^u^ 66 (33/33) participants were included in the final analysis of the respectively study ^v^ 11 (7/4) participants were included in the final analysis of the respectively study ^w^ These intervention groups were not considered for analysis, as medication used were non-commercial substances for the treatment of dementia, as well as non-registered substances of the Austrian Register of Pharmaceutical Specialties | | | | | | | | | | | | | | |
