# Supplementary material for: Effectiveness of computer-based interventions for community-dwelling people with cognitive decline: a systematic review with meta-analyses
Source: BMC Geriatr. 2023 Apr 12;23:229. doi: 10.1186/s12877-023-03941-y (PMC10091663; doi:10.1186/s12877-023-03941-y)
Supplement: Supplementary file 4 — Additional file 4. Meta-analyses and composite scores. [file 12877_2023_3941_MOESM4_ESM.pdf]

Additional file 4

Meta-analyses and composite scores

Table of Contents

Figure 1: Sensitivity meta-analysis; Computer-based cognitive interventions without Dimitriadis 2016 (large SMD) (MCI) vs. control immediately post intervention; Outcome: **Global cognition** 4

Figure 2: Sensitivity meta-analysis; Computer-based cognitive interventions without Amjad 2019 (non-immersive VR technology) (MCI) vs. control immediately post intervention; Outcome: **Global cognition** 5

Figure 3: Composite score; Memory_Finn 2011 (see Figure 4 in Manuscript) 6

Figure 4: Composite score; Memory_Herrera 2012 (see Figure 4 in Manuscript) 7

Figure 5: Composite score; Memory_Nousia 2019 (see Figure 4 in Manuscript) 8

Figure 6: Composite score; Memory_Dimitriadis 2016 (see Figure 4 in Manuscript) 9

Figure 7: Sensitivity meta-analysis; Computer-based cognitive interventions without Dimitriadis 2016(large SMD) (MCI) vs. control immediately post intervention; Outcome: **Memory** 10

Figure 8: Sensitivity meta-analysis; Computer-based cognitive interventions without Hagovska 2017 and Herrera 2012 (non-computer-based cognitive training as control group intervention) (MCI) vs. control immediately post intervention; Outcome: **Memory** 11

Figure 9: Meta-analysis; Computer-based cognitive interventions (MCI) vs. non-computer-based cognitive intervention as control immediately post intervention, Outcome: **Memory** (Composite score Herrera 2012 see Figure 3) 12

Figure 10: Sensitivity meta-analysis; Computer-based cognitive interventions without Hagovska 2017, Herrera 2012 (non-computer-based cognitive training as control group intervention) and Dimitriadis 2016 (large SMD) (MCI) vs. control immediately post intervention; Outcome: **Memory** 13

Figure 11: Meta-analysis; Computer-based cognitive interventions (MCI) vs. control immediately post intervention; Outcome: **Working memory** (see Figure 5 in Manuscript). 14

Figure 12: Composite score; Working memory_Park 2020 (see Figure 11) 15

Figure 13: Composite score; Working memory_Dimitriadis 2016 (see Figure 11) 16

Figure 14: Meta-analysis; Computer-based cognitive interventions (MCI) vs. control immediately post intervention, Outcome: **Attention/concentration/processing speed** (see Figure 5 in Manuscript) 17

Figure 15: Composite score; Attention/concentration/processing speed_Hagovska 2017 (see Figure 14) 18

Figure 16: Composite score; Attention/concentration/processing speed_Savulich 2017 (see Figure 14) 19

Figure 17: Composite score; Attention/concentration/processing speed_Park 2020 (see Figure 14) 20

Figure 18: Meta-analysis; Computer-based cognitive interventions (MCI) vs. control immediately post intervention, Outcome: **Executive functioning** (see Figure 5 in Manuscript) 21

Figure 19: Composite score; Executive functioning_Finn 2011 (see Figure 18) 22

Figure 20: Composite score; Executive functioning_Lin 2016 (see Figure 18) 23

Figure 21: Composite score; Executive functioning_Nousia 2019 (see Figure 18) 24

Figure 22: Composite score; Executive functioning_Park 2020 (see Figure 18) 25

Figure 23: Meta-analysis; Computer-based cognitive interventions (MCI) vs. control immediately post intervention, Outcome: **Language** (see Figure 5 in Manuscript) 26

Figure 24: Meta-analysis; Computer-based cognitive interventions (MCI) vs. computer-based cognitive interventions as control immediately post intervention, Outcome: **Memory** 27

Figure 25: Composite score; Memory_Flak 2019 (see Figure 24) 28

Figure 26: Meta-analysis; Computer-based cognitive interventions (MCI) vs. computer-based cognitive interventions as control immediately post intervention, Outcome: **Working memory** 29
Figure 27: Composite score; Working memory_Flak 2019 (see Figure 26) 30
Figure 28: Meta-analysis; Computer-based cognitive interventions (MCI) vs. computer-based cognitive interventions as control immediately post intervention, Outcome: **Executive functioning** 31
Figure 29: Composite score; Executive functioning_Flak 2019 (see Figure 28) 32
Figure 30: Meta-analysis; Computer-based cognitive interventions (MCI) vs. computer-based cognitive interventions follow-up, Outcome: **Memory** 33
Figure 31: Composite score; Memory_Flak 2019 (see Figure 30) 34
Figure 32: Meta-analysis; Computer-based cognitive interventions (MCI) vs. computer-based cognitive interventions follow-up, Outcome: **Working memory** 35
Figure 33: Composite score; Working memory_Flak 2019 (see Figure 32) 36
Figure 34: Meta-analysis; Computer-based cognitive interventions (MCI) vs. computer-based cognitive interventions follow-up, Outcome: **Executive functioning** 37
Figure 35: Composite score; Executive functioning_Flak 2019 (see Figure 34) 38
Figure 36: Sensitivity meta-analysis; Computer-based cognitive interventions without Cinar 2020 (large SMD) (DEMENTIA) vs. control immediately post intervention, Outcome: **Global cognition** 39
Figure 37: Meta-analysis; Computer-based cognitive interventions (DEMENTIA) vs. control follow-up (3 months), Outcome: **Global cognition** 40
Figure 38: Composite score; Global cognition_Galante 2007 (see Figure 37) 41
Figure 39: Composite score; Memory_Heiss 1994 (see Figure 7 in Manuscript) 42
Figure 40: Composite score; Memory_Lee 2013 (see Figure 7 in Manuscript) 43
Figure 41: Composite score; Memory_Karssemeijer 2019 (see Figure 7 in Manuscript) 44
Figure 42: Meta-analysis; Computer-based cognitive interventions (DEMENTIA) vs. control immediately post intervention, Outcome: **Working memory** (see Figure 8 in Manuscript) 45
Figure 43: Composite score; Working memory_Karssemeijer 2019 (see Figure 42) 46
Figure 44: Meta-analysis; Computer-based cognitive interventions (DEMENTIA) vs. control immediately post intervention, Outcome: **Attention/concentration/processing speed** (see Figure 8 in Manuscript) 47
Figure 45: Composite score; Attention/concentration/processing speed_Karssemeijer 2019 (see Figure 44) 48
Figure 46: Meta-analysis; Computer-based cognitive interventions (DEMENTIA) vs. control immediately post intervention, Outcome: **Executive functioning** (see Figure 8 in Manuscript) 49
Figure 47: Composite score; Executive functioning_Galante 2007 (see Figure 46) 50
Figure 48: Composite score; Executive functioning_Heiss 1994 (see Figure 46) 51
Figure 49: Composite score; Executive functioning_Karssemeijer 2019 (see Figure 46) 52
Figure 50: Meta-analysis; Computer-based cognitive interventions (DEMENTIA) vs. control follow-up (3 months), Outcome: **Memory** 53
Figure 51: Composite score; Memory_Lee 2013 (see Figure 50) 54
Figure 52: Composite score; Memory_Karssemeijer 2019 (see Figure 50) 55
Figure 53: Meta-analysis; Computer-based cognitive interventions (DEMENTIA) vs. control follow-up (3 months), Outcome: **Working memory** 56
Figure 54: Composite score; Working memory_Karssemeijer 2019 (see Figure 53) 57
Figure 55: Meta-analysis; Computer-based cognitive interventions (DEMENTIA) vs. control follow-up (3 months), Outcome: **Attention/concentration/processing speed** 58
Figure 56: Composite score; Attention/concentration/processing speed_Karssemeijer 2019 (see Figure 55) 59
Figure 57: Meta-analysis; Computer-based cognitive interventions (DEMENTIA) vs. control follow-up (3 months), Outcome: **Executive functioning** 60

Figure 58: Composite score; Executive functioning_Galante 2007 (see Figure 57) 61
Figure 59: Composite score; Executive functioning_Karssemeijer 2019 (see Figure 57) 62

Abbreviations:

Cont: Control; Int: Intervention; IV: inverse variance; SD: standard deviation; SE: standard error; SMD; standardized mean difference; 95 % CI: 95 % confidence interval;

Study or Subgroup	Intervention Mean	SD	Total	Control Mean	SD	Total	Weight	Std. Mean Difference IV, Random, 95% CI
**1.1.1 PC**								
Hagovska 2017 (1)	85.63	8.22	30	80.52	7.30	30	23.8%	0.65 [0.13; 1.17]
**Subtotal (95% CI)**			**30**			**30**	**23.8%**	**0.65 [0.13; 1.17]**
Heterogeneity: not applicable								
Test for overall effect: t_{Inf} = 2.45 (P = 0.014)								
**1.1.2 Tablet**								
Savulich 2017 (2)	27.40	1.50	21	26.10	2.40	21	19.8%	0.64 [0.02; 1.26]
**Subtotal (95% CI)**			**21**			**21**	**19.8%**	**0.64 [0.02; 1.26]**
Heterogeneity: not applicable								
Test for overall effect: t_{Inf} = 2.01 (P = 0.044)								
**1.1.3 VR**								
Amjad 2019 (3)	26.25	1.47	18	23.72	3.27	20	18.0%	0.96 [0.28; 1.64]
Park 2020 (4)	25.80	2.74	10	26.82	1.94	11	13.0%	-0.42 [-1.28; 0.45]
Thapa 2020 (5)	26.90	2.00	33	26.40	2.70	33	25.4%	0.21 [-0.28; 0.69]
**Subtotal (95% CI)**			**61**			**64**	**56.4%**	**0.28 [-1.36; 1.92]**
Heterogeneity: Tau^2 = 0.2892; Chi^2 = 6.41, df = 2 (P = 0.041); I^2 = 69%								
Test for overall effect: t_2 = 0.74 (P = 0.535)								
**1.1.4 AR**								
Dimitriadis 2016 (6)	28.10	1.10	53	24.90	1.20	55	0.0%	2.76 [2.23; 3.29]
**Total (95% CI)**			**165**			**170**	**100.0%**	**0.45 [-0.13; 1.03]**
Heterogeneity: Tau^2 = 0.0810; Chi^2 = 7.88, df = 4 (P = 0.096); I^2 = 49%								
Test for overall effect: t_4 = 2.16 (P = 0.097)								
Test for subgroup differences: Chi^2 = 0.70, df = 2 (P = 0.706)								

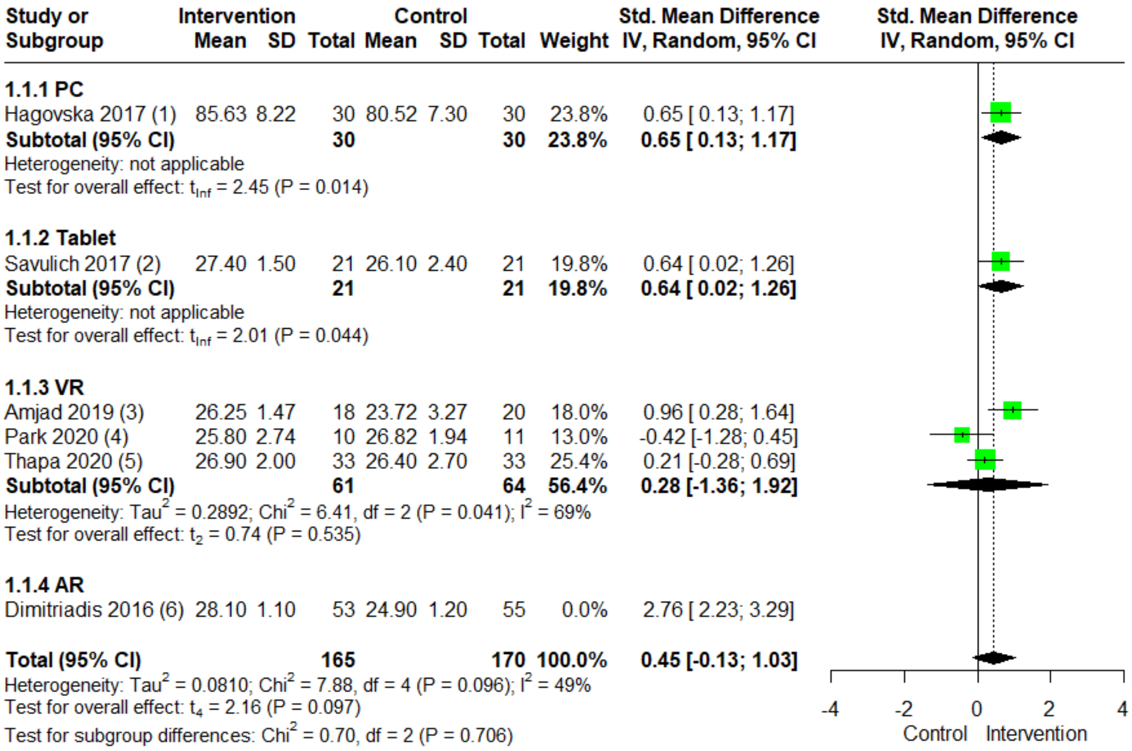

Footnote

(1) Addenbrooke's Cognitive Examination (ACE)
(2) Mini Mental State Examination (MMSE)
(3) MMSE
(4) MMSE
(5) MMSE
(6) MMSE; intervention group vs. control group 2 (CG2)

Figure 1: Sensitivity meta-analysis; Computer-based cognitive interventions without Dimitriadis 2016 (large SMD) (MCI) vs. control immediately post intervention; Outcome: **Global cognition**

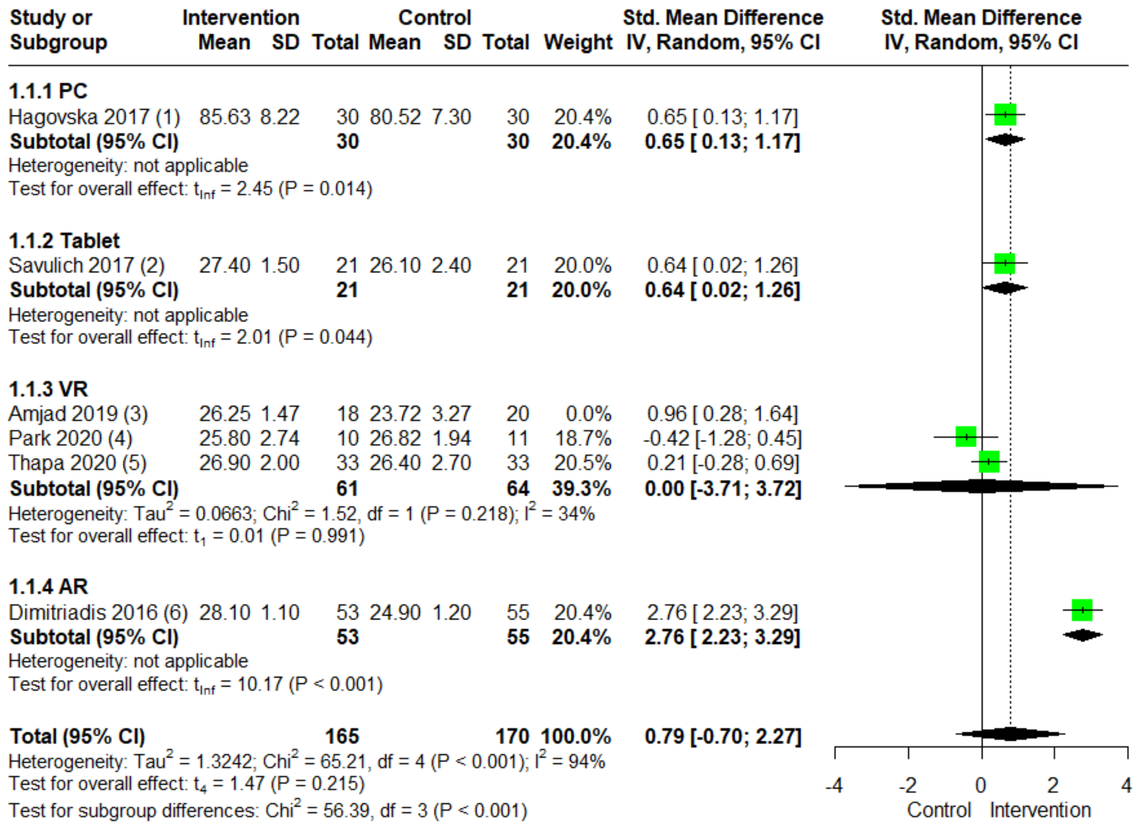

Footnote

(1) Addenbrooke's Cognitive Examination (ACE)
(2) Mini Mental State Examination (MMSE)
(3) MMSE
(4) MMSE
(5) MMSE
(6) MMSE; intervention group vs. control group 2 (CG2)

Figure 2: Sensitivity meta-analysis; Computer-based cognitive interventions without Amjad 2019 (non-immersive VR technology) (MCI) vs. control immediately post intervention; Outcome: **Global cognition**

Study	Intervention Mean	SD	Total	Control Mean	SD	Total	Weight	Std. Mean Difference IV, Random, 95% CI
Finn 2011 (1)	-34.13	17.28	8	-55.33	21.53	8	32.8%	1.03 [-0.03; 2.09]
Finn 2011 (2)	90.62	9.39	8	80.22	15.39	8	35.0%	0.77 [-0.26; 1.80]
Finn 2011 (3)	-28.63	30.28	8	-91.75	71.72	8	32.2%	1.08 [0.01; 2.15]
**Total (95% CI)**			**24**			**24**	**100.0%**	**0.96 [0.54; 1.37]**

Heterogeneity: $Tau^2 = 0$; $Chi^2 = 0.20$, df = 2 (P = 0.91); $I^2 = 0\%$
Test for overall effect: $t_2 = 9.84$ (P = 0.01)

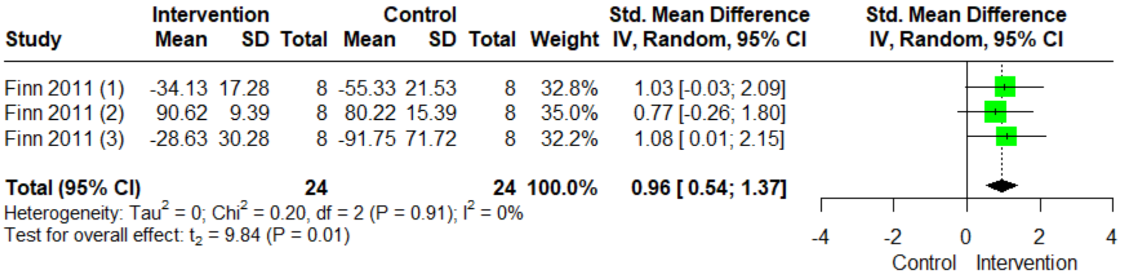

Footnote

(1) CANTAB-Spatial working memory (SWM), errors
(2) CANTAB-Pattern recognition memory (PRM), % correct
(3) CANTAB-Paired associated learning (PAL), error (adj.)

Figure 3: Composite score; Memory_Finn 2011 (see Figure 4 in Manuscript)

Study	Intervention Mean	Intervention SD	Intervention Total	Control Mean	Control SD	Control Total	Weight	Std. Mean Difference IV, Random, 95% CI
Herrera 2012 (1)	9.64	1.76	11	7.64	1.56	11	16.7%	1.16 [0.24; 2.07]
Herrera 2012 (2)	6.36	2.19	11	4.64	1.49	11	17.5%	0.88 [-0.00; 1.77]
Herrera 2012 (3)	96.91	1.92	11	90.82	5.17	11	15.5%	1.50 [0.53; 2.47]
Herrera 2012 (4)	7.28	0.86	11	6.05	0.83	11	15.9%	1.40 [0.45; 2.35]
Herrera 2012 (5)	10.45	4.51	11	10.23	2.89	11	18.7%	0.06 [-0.78; 0.89]
Herrera 2012 (6)	42.91	2.52	11	39.91	1.46	11	15.8%	1.40 [0.45; 2.35]
**Total (95% CI)**			**66**			**66**	**100.0%**	**1.03 [0.45; 1.62]**

Heterogeneity: $Tau^2 = 0.1170$; $Chi^2 = 7.46$, df = 5 ($P = 0.19$); $I^2 = 33\%$
Test for overall effect: $t_5 = 4.52$ ($P < 0.01$)

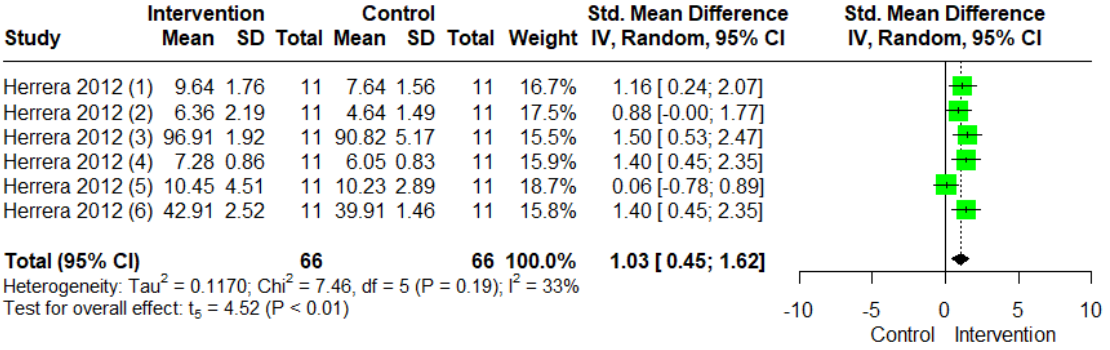

Footnote

(1) Doors recognition subtest (Set A)
(2) Doors recognition subtest (Set B)
(3) Delayed Matching Sample Task (DMS48)
(4) BEM144 (12-word-list-recall test), total score
(5) Rey-Osterreith Complex Figure, recall/36
(6) 16-item free and cued reminding test

Figure 4: Composite score; Memory_Herrera 2012 (see Figure 4 in Manuscript)

Study	Intervention Mean	Intervention SD	Intervention Total	Control Mean	Control SD	Control Total	Weight	Std. Mean Difference IV, Random, 95% CI
Nousia 2019 (1)	21.00	2.72	25	20.57	2.93	21	33.8%	0.15 [-0.43; 0.73]
Nousia 2019 (2)	3.04	1.21	25	0.67	0.58	21	32.4%	2.39 [1.62; 3.16]
Nousia 2019 (3)	19.68	0.48	25	19.48	0.81	21	33.8%	0.30 [-0.28; 0.89]
**Total (95% CI)**			**75**			**63**	**100.0%**	**0.93 [-2.16; 4.01]**

Heterogeneity: $Tau^2 = 1.4085$; $Chi^2 = 23.60$, df = 2 ($P < 0.01$); $I^2 = 92\%$
Test for overall effect: $t_2 = 1.29$ ($P = 0.33$)

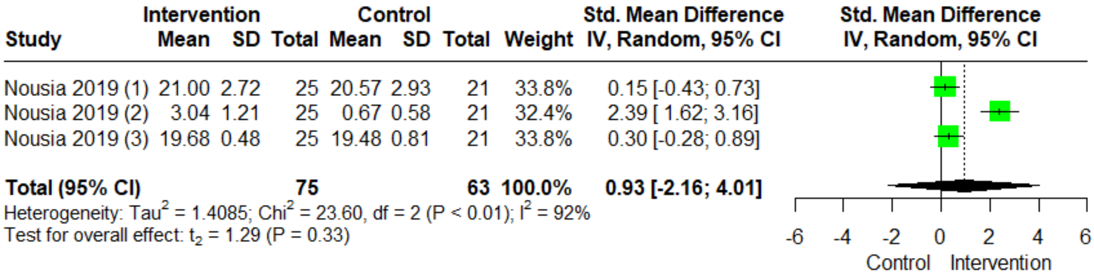

Footnote

(1) Recall, word recognition and delayed word memory test, recall
(2) Recall, word recognition and delayed word memory test, delayed memory
(3) Recall, word recognition and delayed word memory test, word recognition

Figure 5: Composite score; Memory_Nousia 2019 (see Figure 4 in Manuscript)

Study	Intervention Mean	Intervention SD	Intervention Total	Control Mean	Control SD	Control Total	Weight	Std. Mean Difference IV, Random, 95% CI
Dimitriadis 2016 (1)	10.80	1.50	53	7.80	1.80	55	20.2%	1.79 [1.35; 2.24]
Dimitriadis 2016 (2)	-1.10	1.00	53	-4.80	1.10	55	20.1%	3.49 [2.89; 4.10]
Dimitriadis 2016 (3)	-0.60	0.40	53	-3.60	0.30	55	19.4%	8.45 [7.24; 9.65]
Dimitriadis 2016 (4)	-1.10	1.00	53	-2.50	1.10	55	20.2%	1.32 [0.90; 1.74]
Dimitriadis 2016 (5)	10.50	1.80	53	6.10	0.60	55	20.1%	3.28 [2.70; 3.86]
**Total (95% CI)**			**265**			**275**	**100.0%**	**3.63 [0.15; 7.11]**

Heterogeneity: $Tau^2 = 7.5715$; $Chi^2 = 150.19$, df = 4 ($P < 0.01$); $I^2 = 97\%$
Test for overall effect: $t_4 = 2.89$ ($P = 0.04$)

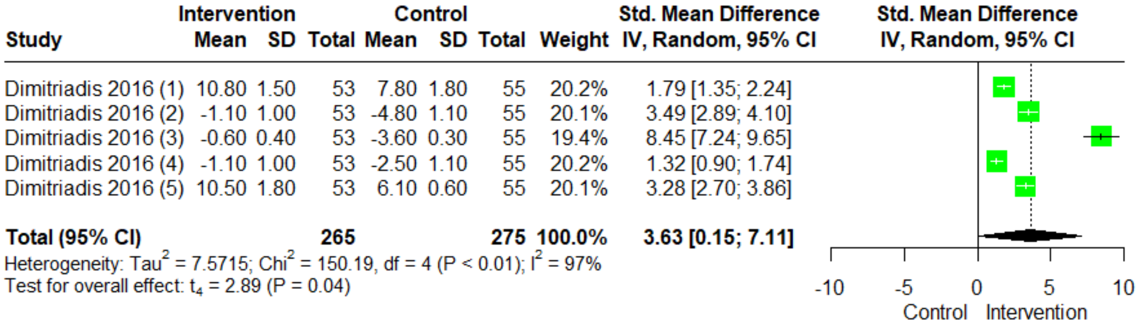

Footnote

(1) California Verbal Learning Test (CVLT) II, immediate recall
(2) CVLT II, perseverative errors
(3) CVLT II, intrusions error
(4) CVLT II, memory decay
(5) CVLT II, delayed recall

Figure 6: Composite score; Memory_Dimitriadis 2016 (see Figure 4 in Manuscript)

Study or Subgroup	SMD	SE	Int Total	Cont Total	Weight	Std. Mean Difference IV, Random, 95% CI
**1.3.1 PC**						
Finn 2011 (1)	0.96	0.5369	8	8	12.0%	0.96 [-0.09; 2.01]
Hagovska 2017 (2)	-0.19	0.2588	30	30	22.9%	-0.19 [-0.70; 0.32]
Herrera 2012 (3)	1.03	0.4596	11	11	14.4%	1.03 [0.13; 1.93]
Nousia 2019 (4)	0.93	0.3125	25	21	20.4%	0.93 [0.31; 1.54]
Rosen 2011 (5)	0.65	0.5996	6	6	10.4%	0.65 [-0.53; 1.83]
**Subtotal (95% CI)**			**80**	**76**	**80.1%**	**0.61 [-0.09; 1.30]**
Heterogeneity: $Tau^2 = 0.2260$; $Chi^2 = 11.04$, df = 4 (P = 0.026); $I^2 = 64\%$						
Test for overall effect: $t_4 = 2.42$ (P = 0.073)						
**1.3.2 Tablet**						
Savulich 2017 (6)	0.83	0.3229	21	21	19.9%	0.83 [0.20; 1.46]
**Subtotal (95% CI)**			**21**	**21**	**19.9%**	**0.83 [0.20; 1.46]**
Heterogeneity: not applicable						
Test for overall effect: $t_{Inf} = 2.57$ (P = 0.010)						
**1.3.3 AR**						
Dimitriadis 2016 (7)	3.63	0.3135	53	55	0.0%	3.63 [3.01; 4.24]
**Total (95% CI)**			**154**	**152**	**100.0%**	**0.64 [0.11; 1.18]**
Heterogeneity: $Tau^2 = 0.1760$; $Chi^2 = 12.07$, df = 5 (P = 0.034); $I^2 = 59\%$						
Test for overall effect: $t_5 = 3.08$ (P = 0.027)						
Test for subgroup differences: $Chi^2 = 0.30$, df = 1 (P = 0.585)						

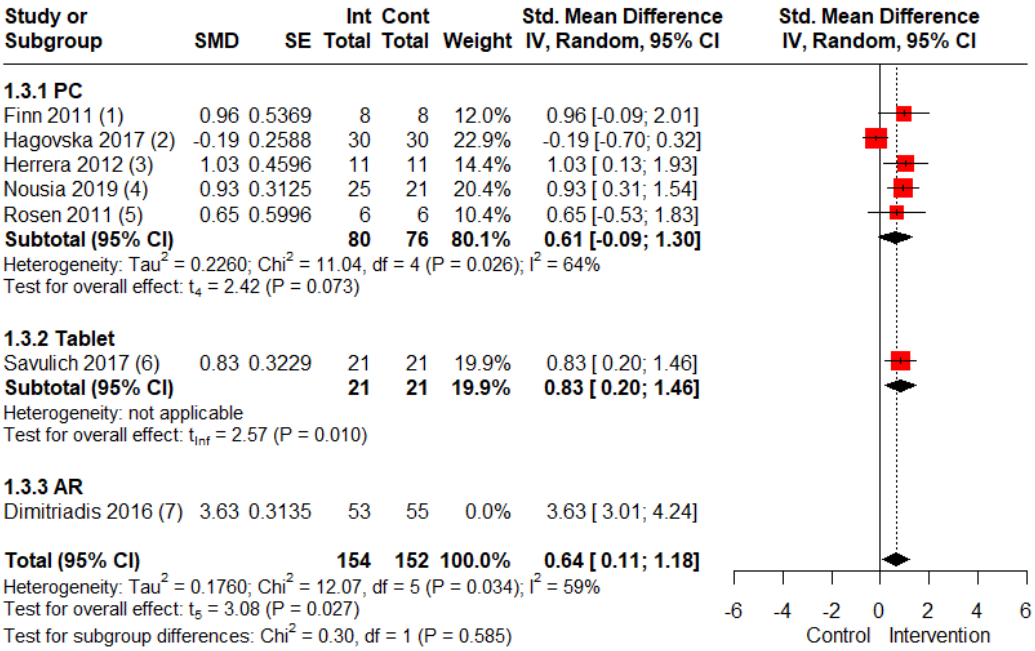

Footnote

(1) Composite score (n=3)
(2) Addenbrooke's Cognitive Examination (ACE), memory
(3) Composite score (n=6)
(4) Composite score (n=3)
(5) Repeatable Battery for Assessment of Cognitive Status (RBANS), immediate memory
(6) Brief Visuospatial Memory Test-Revised (BMVT-R)
(7) Composite score (n=5); intervention group vs. control group 2 (CG2)

Figure 7: Sensitivity meta-analysis; Computer-based cognitive interventions without Dimitriadis 2016(large SMD) (MCI) vs. control immediately post intervention; Outcome: **Memory**

Study or Subgroup	SMD	SE	Int Total	Cont Total	Weight	Std. Mean Difference IV, Random, 95% CI
**1.3.1 PC**						
Finn 2011 (1)	0.96	0.5369	8	8	18.8%	0.96 [-0.09; 2.01]
Hagovska 2017 (2)	-0.19	0.2588	30	30	0.0%	-0.19 [-0.70; 0.32]
Herrera 2012 (3)	1.03	0.4596	11	11	0.0%	1.03 [0.13; 1.93]
Nousia 2019 (4)	0.93	0.3125	25	21	21.1%	0.93 [0.31; 1.54]
Rosen 2011 (5)	0.65	0.5996	6	6	18.1%	0.65 [-0.53; 1.83]
**Subtotal (95% CI)**			**80**	**76**	**58.0%**	**0.89 [0.56; 1.21]**
Heterogeneity: $Tau^2 = 0$; $Chi^2 = 0.19$, df = 2 ($P = 0.909$); $I^2 = 0\%$						
Test for overall effect: $t_2 = 11.68$ ($P = 0.007$)						
**1.3.2 Tablet**						
Savulich 2017 (6)	0.83	0.3229	21	21	21.0%	0.83 [0.20; 1.46]
**Subtotal (95% CI)**			**21**	**21**	**21.0%**	**0.83 [0.20; 1.46]**
Heterogeneity: not applicable						
Test for overall effect: $t_{Inf} = 2.57$ ($P = 0.010$)						
**1.3.3 AR**						
Dimitriadis 2016 (7)	3.63	0.3135	53	55	21.1%	3.63 [3.01; 4.24]
**Subtotal (95% CI)**			**53**	**55**	**21.1%**	**3.63 [3.01; 4.24]**
Heterogeneity: not applicable						
Test for overall effect: $t_{Inf} = 11.57$ ($P < 0.001$)						
**Total (95% CI)**			**154**	**152**	**100.0%**	**1.43 [-0.15; 3.01]**
Heterogeneity: $Tau^2 = 1.5000$; $Chi^2 = 56.05$, df = 4 ($P < 0.001$); $I^2 = 93\%$						
Test for overall effect: $t_4 = 2.51$ ($P = 0.066$)						
Test for subgroup differences: $Chi^2 = 72.62$, df = 2 ($P < 0.001$)						

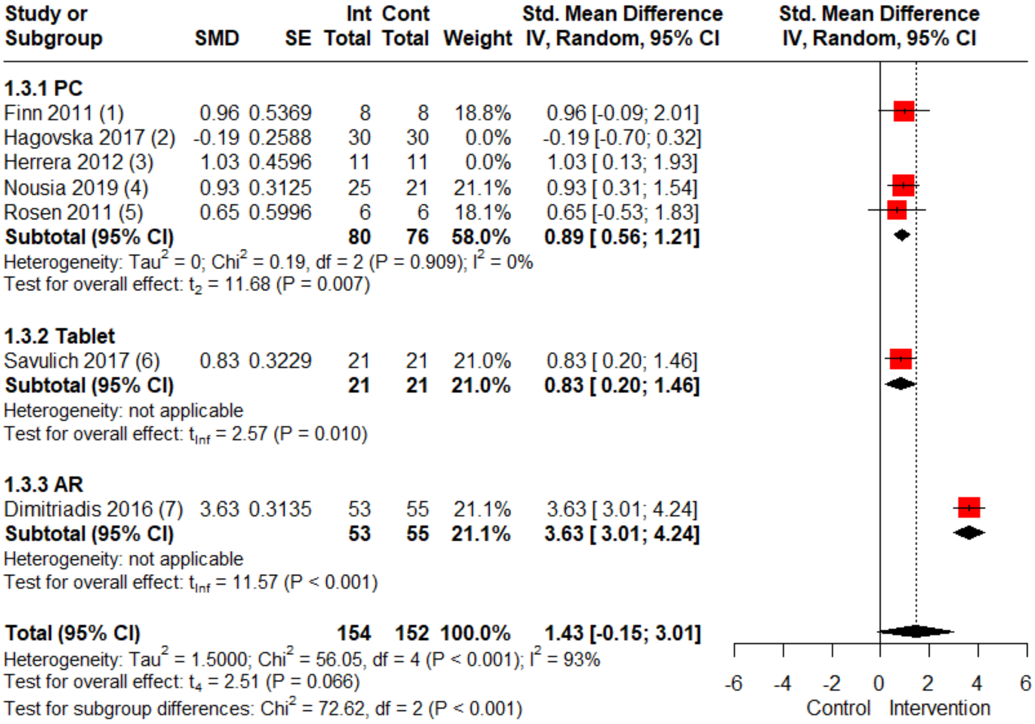

Footnote

(1) Composite score (n=3)
(2) Addenbrooke's Cognitive Examination (ACE), memory
(3) Composite score (n=6)
(4) Composite score (n=3)
(5) Repeatable Battery for Assessment of Cognitive Status (RBANS), immediate memory
(6) Brief Visuospatial Memory Test-Revised (BMVT-R)
(7) Composite score (n=5); intervention group vs. control group 2 (CG2)

Figure 8: Sensitivity meta-analysis; Computer-based cognitive interventions without Hagovska 2017 and Herrera 2012 (non-computer-based cognitive training as control group intervention) (MCI) vs. control immediately post intervention; Outcome: **Memory**

Study or Subgroup	SMD	SE	Int Total	Cont Total	Weight	Std. Mean Difference IV, Random, 95% CI
**1.8.1 PC**						
Hagovska 2017 (1)	-0.19	0.2588	30	30	54.8%	-0.19 [-0.70; 0.32]
Herrera 2012 (2)	1.03	0.4596	11	11	45.2%	1.03 [0.13; 1.93]
**Subtotal (95% CI)**			**41**	**41**	**100.0%**	**0.36 [-7.35; 8.08]**
Heterogeneity: Tau^2 = 0.6051; Chi^2 = 5.35, df = 1 (P = 0.021); I^2 = 81%						
Test for overall effect: t_1 = 0.59 (P = 0.659)						
**Total (95% CI)**			**41**	**41**	**100.0%**	**0.36 [-7.35; 8.08]**
Heterogeneity: Tau^2 = 0.6051; Chi^2 = 5.35, df = 1 (P = 0.021); I^2 = 81%						
Test for overall effect: t_1 = 0.59 (P = 0.659)						
Test for subgroup differences: Chi^2 = 0.00, df = 0 (P = NA)						

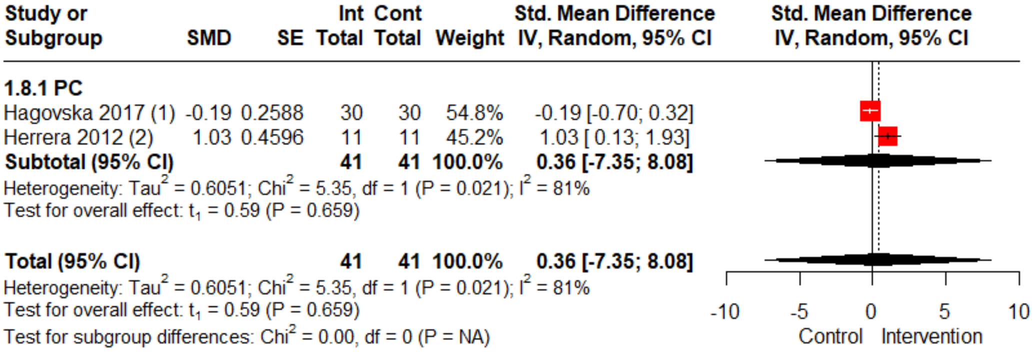

Footnote

(1) Addenbrooke's Cognitive Examination (ACE), memory

(2) Composite score (n= 6)

Figure 9: Meta-analysis; Computer-based cognitive interventions (MCI) vs. non-computer-based cognitive intervention as control immediately post intervention, Outcome: **Memory** (Composite score Herrera 2012 see Figure 3)

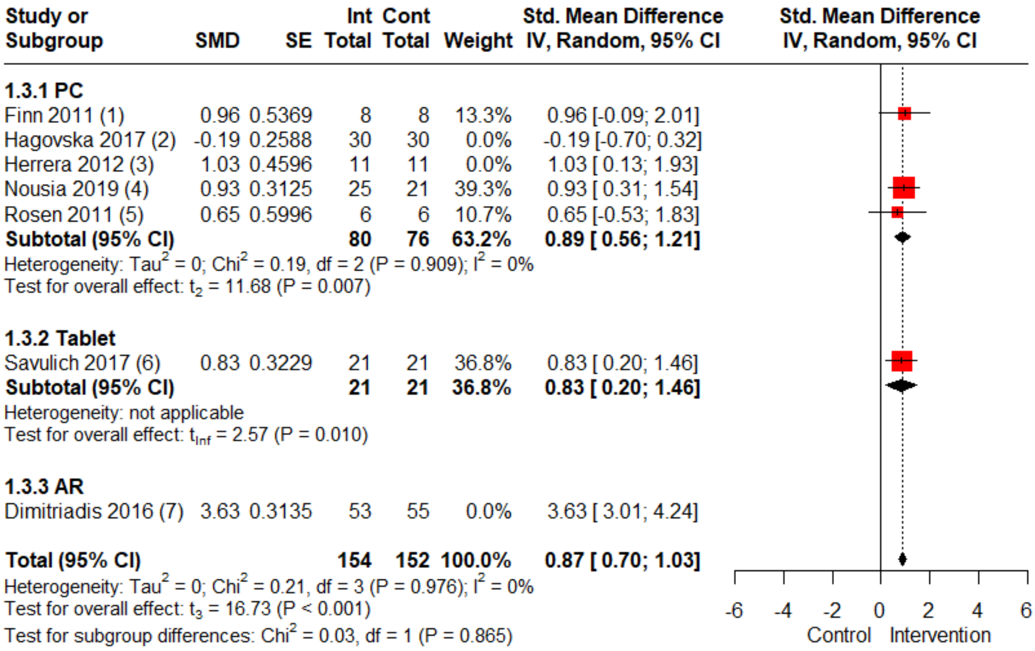

Footnote

(1) Composite score (n=3)
(2) Addenbrooke's Cognitive Examination (ACE), memory
(3) Composite score (n=6)
(4) Composite score (n=3)
(5) Repeatable Battery for Assessment of Cognitive Status (RBANS), immediate memory
(6) Brief Visuospatial Memory Test-Revised (BMVT-R)
(7) Composite score (n=5); intervention group vs. control group 2 (CG2)

Figure 10: Sensitivity meta-analysis; Computer-based cognitive interventions without Hagovska 2017, Herrera 2012 (non-computer-based cognitive training as control group intervention) and Dimitriadis 2016 (large SMD) (MCI) vs. control immediately post intervention; Outcome: **Memory**

Study or Subgroup	SMD	SE	Int Total	Cont Total	Weight	Std. Mean Difference IV, Random, 95% CI
**1.9.1 PC**						
Damirchi 2018 (1)	1.16	0.4939	11	9	13.4%	1.16 [0.19; 2.13]
Herrera 2012 (2)	1.24	0.4736	11	11	14.0%	1.24 [0.31; 2.17]
Lin 2016 (3)	0.27	0.4394	10	11	15.0%	0.27 [-0.59; 1.13]
Nousia 2019 (4)	0.42	0.2995	25	21	19.9%	0.42 [-0.17; 1.01]
**Subtotal (95% CI)**			**57**	**52**	**62.2%**	**0.69 [-0.07; 1.44]**
Heterogeneity: $Tau^2 = 0.0495$; $Chi^2 = 3.94$, df = 3 (P = 0.268); $I^2 = 24\%$						
Test for overall effect: $t_3 = 2.89$ (P = 0.063)						
**1.9.2 VR**						
Park 2020 (5)	0.33	0.4406	10	11	15.0%	0.33 [-0.53; 1.19]
**Subtotal (95% CI)**			**10**	**11**	**15.0%**	**0.33 [-0.53; 1.19]**
Heterogeneity: not applicable						
Test for overall effect: $t_{Inf} = 0.75$ (P = 0.454)						
**1.9.3 AR**						
Dimitriadis 2016 (6)	1.57	0.2211	53	55	22.8%	1.57 [1.14; 2.00]
**Subtotal (95% CI)**			**53**	**55**	**22.8%**	**1.57 [1.14; 2.00]**
Heterogeneity: not applicable						
Test for overall effect: $t_{Inf} = 7.10$ (P < 0.001)						
**Total (95% CI)**			**120**	**118**	**100.0%**	**0.86 [0.25; 1.47]**
Heterogeneity: $Tau^2 = 0.2293$; $Chi^2 = 15.82$, df = 5 (P = 0.007); $I^2 = 68\%$						
Test for overall effect: $t_5 = 3.63$ (P = 0.015)						
Test for subgroup differences: $Chi^2 = 10.55$, df = 2 (P = 0.005)						

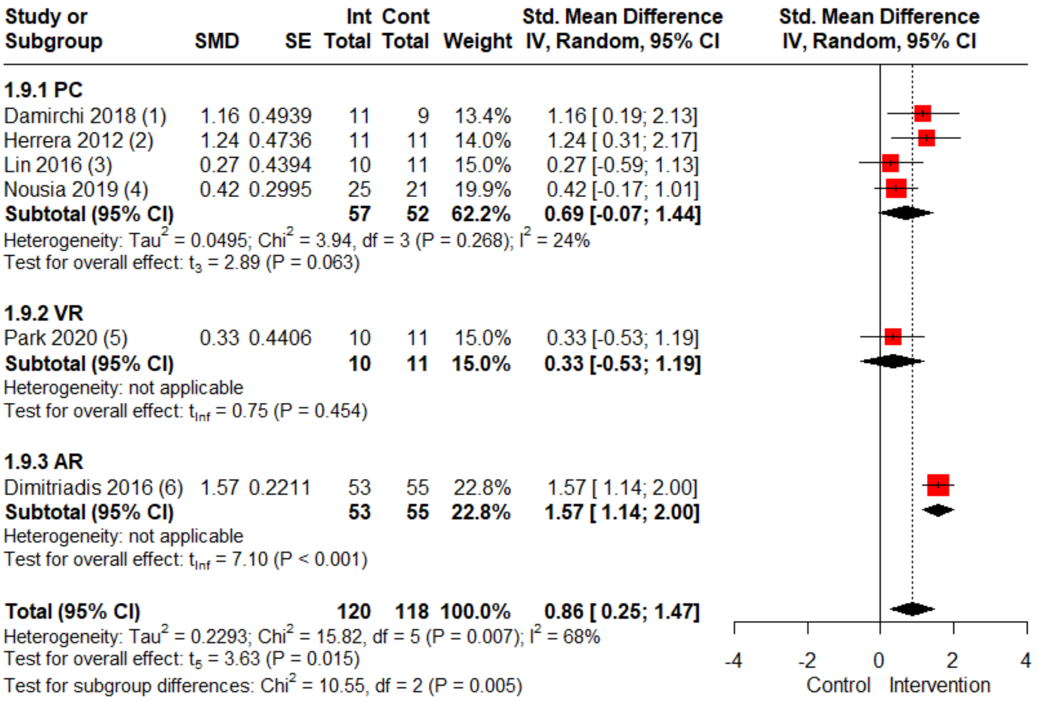

Footnote

(1) Digit span forward
(2) Digit span forward
(3) EXAMINER working memory (computerized test)
(4) Digit span forward
(5) Composite score (n=2)
(6) Composite score (n=2)

Figure 11: Meta-analysis; Computer-based cognitive interventions (MCI) vs. control immediately post intervention; Outcome: **Working memory** (see Figure 5 in Manuscript).

Study	Intervention Mean	Intervention SD	Intervention Total	Control Mean	Control SD	Control Total	Weight	Std. Mean Difference IV, Random, 95% CI
Park 2020 (1)	6.60	1.64	10	5.91	1.30	11	49.4%	0.45 [-0.42; 1.32]
Park 2020 (2)	3.60	1.07	10	3.36	1.03	11	50.6%	0.22 [-0.64; 1.08]
**Total (95% CI)**			**20**			**22**	**100.0%**	**0.33 [-1.13; 1.80]**

Heterogeneity: $Tau^2 = 0$; $Chi^2 = 0.14$, df = 1 (P = 0.71); $I^2 = 0\%$
Test for overall effect: $t_1 = 2.89$ (P = 0.21)

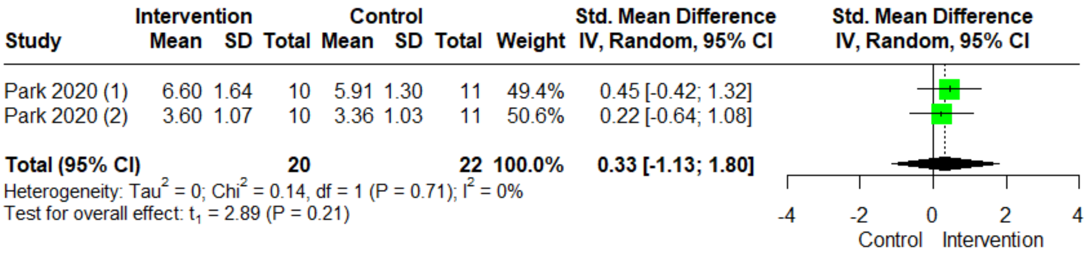

Footnote

(1) Digit span forward
(2) Digit span backward

Figure 12: Composite score; Working memory_Park 2020 (see Figure 11)

Study	Intervention			Control			Weight	Std. Mean Difference
	Mean	SD	Total	Mean	SD	Total		IV, Random, 95% CI
Dimitriadis 2016 (1)	6.70	0.60	53	5.80	0.60	55	51.3%	1.49 [1.06; 1.92]
Dimitriadis 2016 (2)	5.10	0.60	53	4.10	0.60	55	48.7%	1.65 [1.22; 2.09]
**Total (95% CI)**			**106**			**110**	**100.0%**	**1.57 [0.52; 2.62]**

Heterogeneity: $Tau^2 = 0$; $Chi^2 = 0.28$, df = 1 (P = 0.60); $I^2 = 0\%$

Test for overall effect: $t_1 = 18.98$ (P = 0.03)

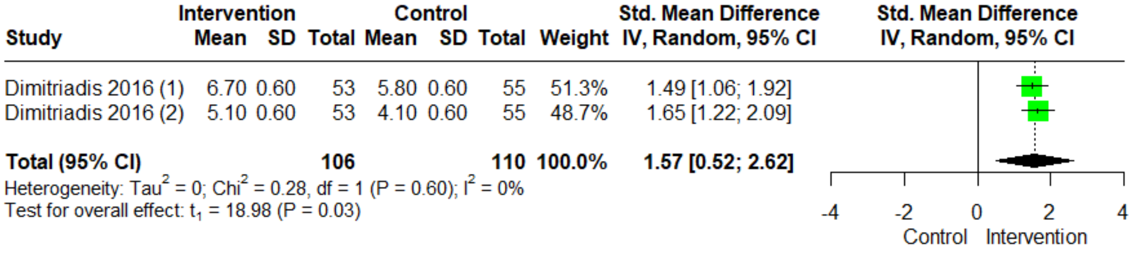

Footnote

(1) Digit span forward

(2) Digit span backward

Figure 13: Composite score; Working memory_Dimitriadis 2016 (see Figure 11)

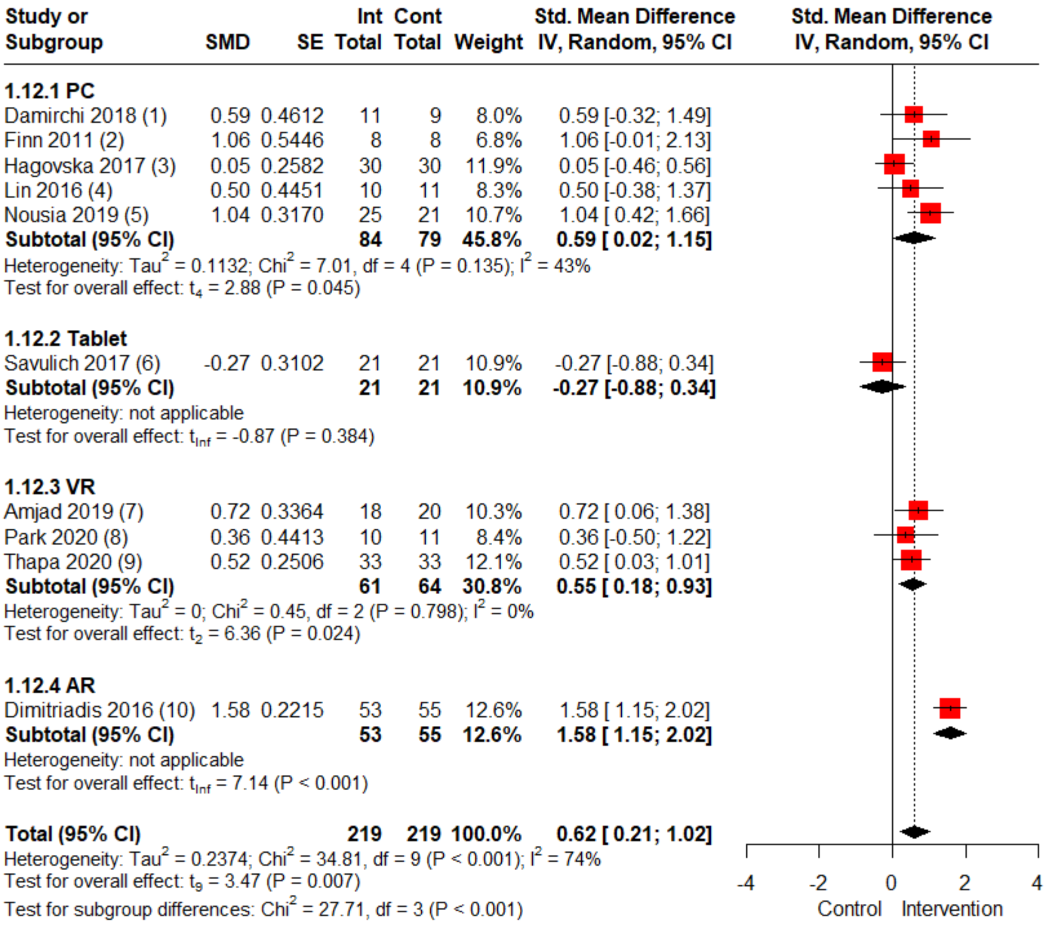

Footnote

(1) Digit symbol coding
(2) CANTAB-Rapid visual information processing (RVP)
(3) Composite score (n=3)
(4) Useful Field of View (UFOV) (computerized test)
(5) TMT A
(6) Composite score (n=2)
(7) TMT A
(8) Composite score (n=2)
(9) TMT A
(10) TMT A

Figure 14: Meta-analysis; Computer-based cognitive interventions (MCI) vs. control immediately post intervention, Outcome: **Attention/concentration/processing speed** (see Figure 5 in Manuscript)

Study	Intervention Mean	SD	Total	Control Mean	SD	Total	Weight	Std. Mean Difference IV, Random, 95% CI
Hagovska 2017 (1)	17.50	2.77	30	17.10	1.79	30	33.3%	0.17 [-0.34; 0.68]
Hagovska 2017 (2)	-63.75	23.09	30	-66.10	32.03	30	33.4%	0.08 [-0.42; 0.59]
Hagovska 2017 (3)	-67.84	20.36	30	-64.92	30.64	30	33.3%	-0.11 [-0.62; 0.40]
**Total (95% CI)**			**90**			**90**	**100.0%**	**0.05 [-0.31; 0.40]**

Heterogeneity: $Tau^2 = 0$; $Chi^2 = 0.62$, df = 2 (P = 0.73); $I^2 = 0\%$
Test for overall effect: $t_2 = 0.57$ (P = 0.63)

Std. Mean Difference
IV, Random, 95% CI

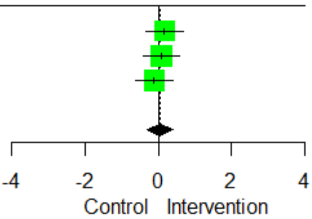

Footnote

(1) Addenbrooke's Cognitive Examination (ACE), attention/concentration
(2) Stroop Color-Word Test (SCWT), words, personal tempo, time (sec.))
(3) SCWT, colors, perception factor (sec.)

Figure 15: Composite score; Attention/concentration/processing speed_Hagovska 2017 (see Figure 14)

Study	Intervention Mean	Intervention SD	Intervention Total	Control Mean	Control SD	Control Total	Weight	Std. Mean Difference IV, Random, 95% CI
Savulich 2017 (1)	98.40	1.60	21	99.10	1.90	21	49.6%	-0.39 [-1.00; 0.22]
Savulich 2017 (2)	-541.00	187.60	21	-512.60	192.50	21	50.4%	-0.15 [-0.75; 0.46]
**Total (95% CI)**			**42**			**42**	**100.0%**	**-0.27 [-1.82; 1.28]**

Heterogeneity: $Tau^2 = 0$; $Chi^2 = 0.31$, df = 1 (P = 0.58); $I^2 = 0\%$
Test for overall effect: $t_1 = -2.19$ (P = 0.27)

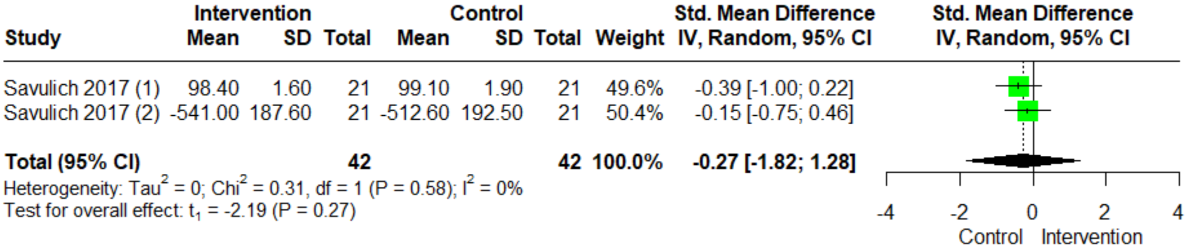

Footnote

(1) CANTAB-Choice reaction time (CRT), correct trials

(2) CANTAB-Choice reaction time (CRT), time latency

Figure 16: Composite score; Attention/concentration/processing speed_Savulich 2017 (see Figure 14)

Study	Intervention Mean	SD	Total	Control Mean	SD	Total	Weight	Std. Mean Difference IV, Random, 95% CI	Std. Mean Difference IV, Random, 95% CI
Park 2020 (1)	-100.60	20.80	10	-105.82	12.34	11	50.4%	0.30 [-0.57; 1.16]	
Park 2020 (2)	-58.10	35.71	10	-73.91	34.96	11	49.6%	0.43 [-0.44; 1.30]	
**Total (95% CI)**			**20**			**22**	**100.0%**	**0.36 [-0.48; 1.21]**	

Heterogeneity: $Tau^2 = 0$; $Chi^2 = 0.05$, df = 1 (P = 0.83); $I^2 = 0\%$
Test for overall effect: $t_1 = 5.46$ (P = 0.12)

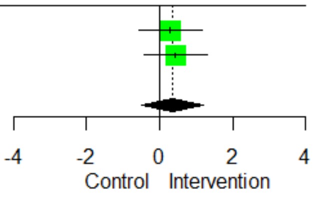

Footnote

(1) SCWT, color word interference test, naming (ie. word), score
(2) SCWT, color word interference test, reading (ie. color), score

Figure 17: Composite score; Attention/concentration/processing speed_Park 2020 (see Figure 14)

Study or Subgroup	SMD	SE	Int Total	Cont Total	Weight	Std. Mean Difference IV, Random, 95% CI
**1.16.1 PC**						
Finn 2011 (1)	1.13	0.5504	8	8	10.4%	1.13 [0.05; 2.21]
Hagovska 2017 (2)	0.19	0.2588	30	30	13.6%	0.19 [-0.32; 0.69]
Lin 2016 (3)	0.02	0.4369	10	11	11.7%	0.02 [-0.84; 0.88]
Nousia 2019 (4)	1.20	0.3236	25	21	13.0%	1.20 [0.57; 1.83]
**Subtotal (95% CI)**			**73**	**70**	**48.7%**	**0.60 [-0.37; 1.58]**
Heterogeneity: $Tau^2 = 0.2392$; $Chi^2 = 8.59$, df = 3 (P = 0.035); $I^2 = 65\%$						
Test for overall effect: $t_3 = 1.97$ (P = 0.144)						
**1.16.2 VR**						
Amjad 2019 (5)	1.57	0.3767	18	20	12.4%	1.57 [0.84; 2.31]
Park 2020 (6)	0.37	0.4415	10	11	11.7%	0.37 [-0.50; 1.24]
Thapa 2020 (7)	0.47	0.2498	33	33	13.7%	0.47 [-0.02; 0.96]
**Subtotal (95% CI)**			**61**	**64**	**37.8%**	**0.80 [-0.83; 2.43]**
Heterogeneity: $Tau^2 = 0.2983$; $Chi^2 = 6.75$, df = 2 (P = 0.034); $I^2 = 70\%$						
Test for overall effect: $t_2 = 2.10$ (P = 0.170)						
**1.16.3 AR**						
Dimitriadis 2016 (8)	2.65	0.2661	53	55	13.5%	2.65 [2.13; 3.17]
**Subtotal (95% CI)**			**53**	**55**	**13.5%**	**2.65 [2.13; 3.17]**
Heterogeneity: not applicable						
Test for overall effect: $t_{Inf} = 9.96$ (P < 0.001)						
**Total (95% CI)**			**187**	**189**	**100.0%**	**0.96 [0.21; 1.71]**
Heterogeneity: $Tau^2 = 0.7049$; $Chi^2 = 62.60$, df = 7 (P < 0.001); $I^2 = 89\%$						
Test for overall effect: $t_7 = 3.04$ (P = 0.019)						
Test for subgroup differences: $Chi^2 = 30.62$, df = 2 (P < 0.001)						

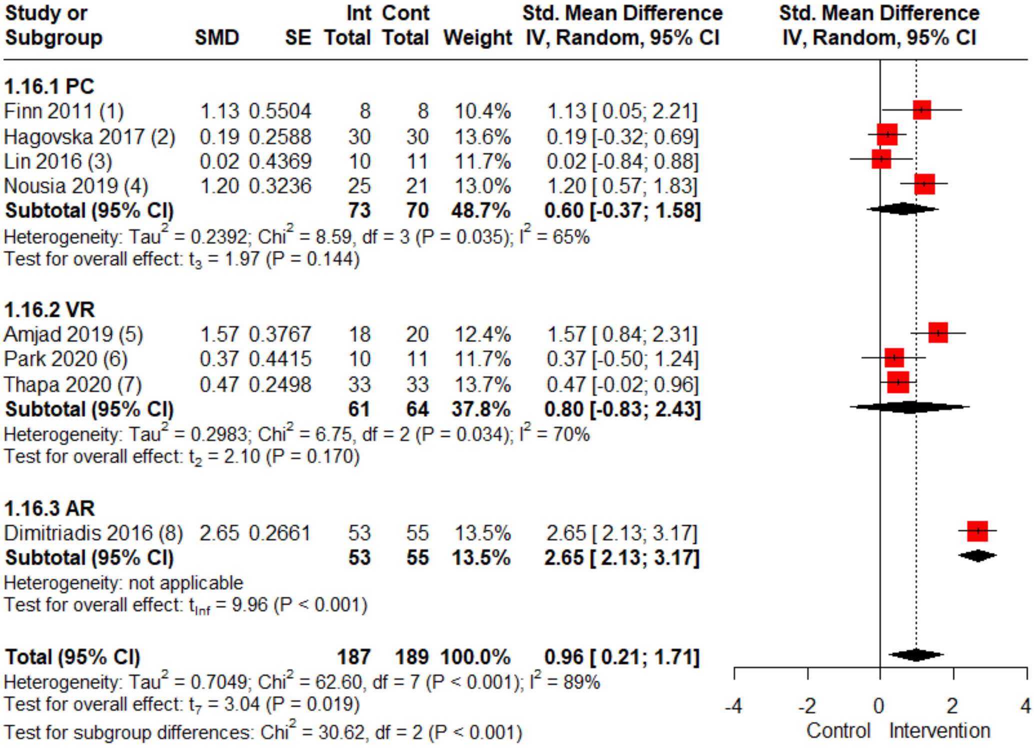

Footnote

(1) Composite score (n=2)
(2) Addenbrooke's Cognitive Examination (ACE), verbal fluency
(3) Composite score (n=2)
(4) Composite score (n=2)
(5) TMT B
(6) Composite score (n=4)
(7) TMT B
(8) TMT B

Figure 18: Meta-analysis; Computer-based cognitive interventions (MCI) vs. control immediately post intervention, Outcome: **Executive functioning** (see Figure 5 in Manuscript)

Study	Intervention Mean	SD	Total	Control Mean	SD	Total	Weight	Std. Mean Difference IV, Random, 95% CI
Finn 2011 (1)	-33.62	6.47	8	-39.13	2.10	8	50.7%	1.08 [0.01; 2.15]
Finn 2011 (2)	-22.25	22.11	8	-94.13	78.61	8	49.3%	1.18 [0.09; 2.26]
**Total (95% CI)**			**16**			**16**	**100.0%**	**1.13 [0.53; 1.73]**

Heterogeneity: $Tau^2 = 0$; $Chi^2 = 0.01$, df = 1 (P = 0.90); $I^2 = 0\%$
Test for overall effect: $t_1 = 24.06$ (P = 0.03)

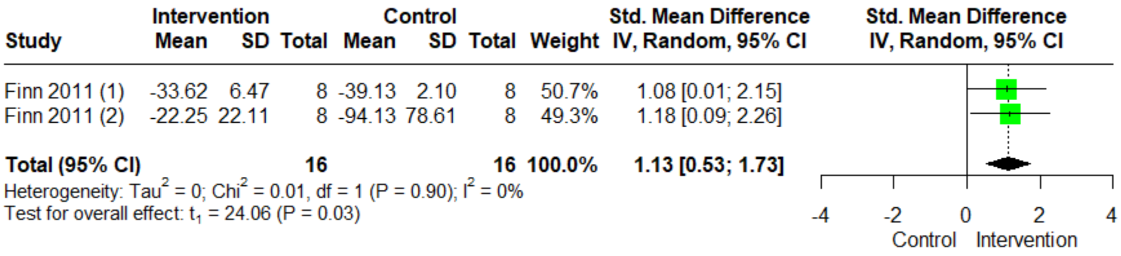

Footnote
(1) CANTAB-Spatial-working memory (SWM) strategy
(2) CANTAB- Intra-/extra-dimensional set shifting (IED), errors (adj.)

Figure 19: Composite score; Executive functioning_Finn 2011 (see Figure 18)

Study	Intervention Mean	Intervention SD	Intervention Total	Control Mean	Control SD	Control Total	Weight	Std. Mean Difference IV, Random, 95% CI
Lin 2016 (1)	0.50	0.57	10	0.21	0.70	11	49.9%	0.43 [-0.43; 1.30]
Lin 2016 (2)	0.26	0.38	10	0.49	0.68	11	50.1%	-0.40 [-1.26; 0.47]
**Total (95% CI)**			**20**			**22**	**100.0%**	**0.02 [-5.25; 5.29]**

Heterogeneity: $Tau^2 = 0.1479$; $Chi^2 = 1.75$, df = 1 (P = 0.19); $I^2 = 43\%$
Test for overall effect: $t_1 = 0.04$ (P = 0.97)

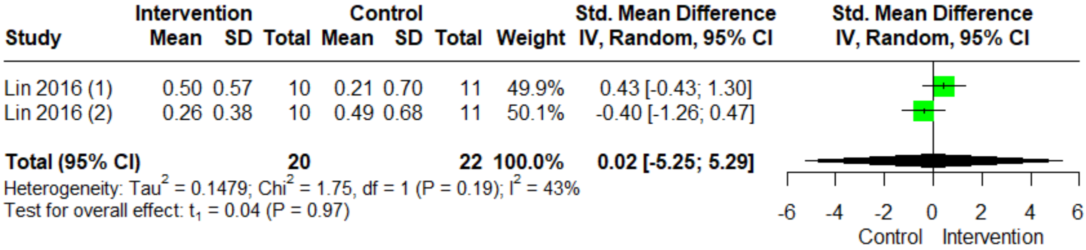

Footnote

(1) EXAMINER, verbal fluency
(2) EXAMINER, cognitive control (set shift and flanker task)

Figure 20: Composite score; Executive functioning_Lin 2016 (see Figure 18)

Study	Intervention Mean	Intervention SD	Intervention Total	Control Mean	Control SD	Control Total	Weight	Std. Mean Difference IV, Random, 95% CI
Nousia 2019 (1)	40.60	7.17	25	34.90	5.54	21	52.1%	0.86 [0.26; 1.47]
Nousia 2019 (2)	-174.16	37.11	25	-237.86	43.73	21	47.9%	1.56 [0.89; 2.22]
**Total (95% CI)**			**50**			**42**	**100.0%**	**1.20 [-3.19; 5.58]**

Heterogeneity: $Tau^2 = 0.1322$; $Chi^2 = 2.24$, df = 1 (P = 0.13); $I^2 = 55\%$
Test for overall effect: $t_1 = 3.46$ (P = 0.18)

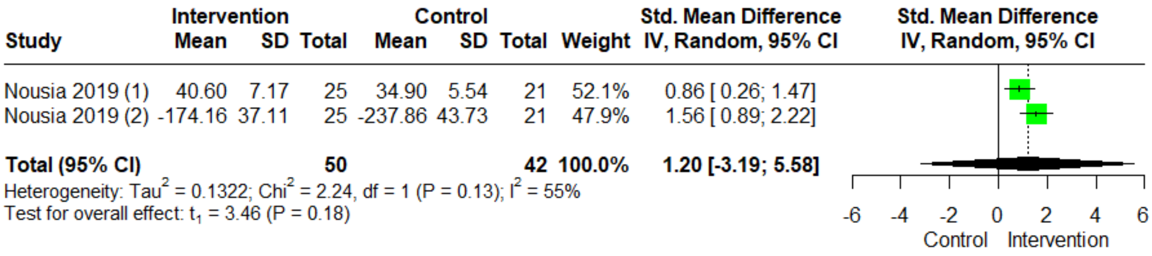

Footnote

(1) Semantic Fluency measure (SF)
(2) TMT B

Figure 21: Composite score; Executive functioning_Nousia 2019 (see Figure 18)

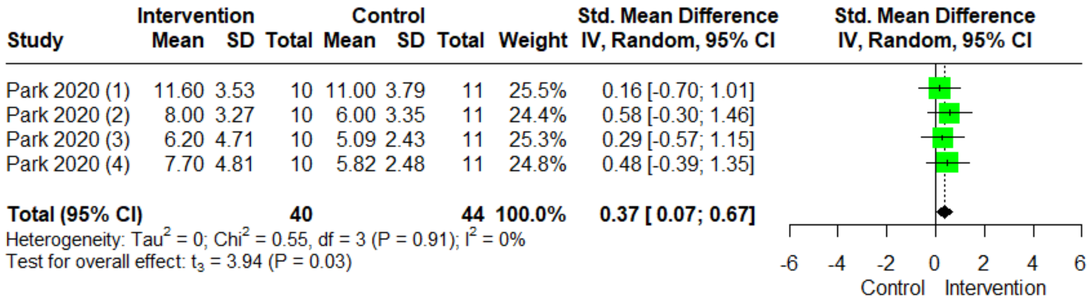

Footnote

(1) Word fluency test (animal)
(2) Word fluency test (Korean names 1)
(3) Word fluency test (Korean names 2)
(4) Word fluency test (Korean names 3)

Figure 22: Composite score; Executive functioning_Park 2020 (see Figure 18)

Study or Subgroup	Intervention Mean	Intervention SD	Intervention Total	Control Mean	Control SD	Control Total	Weight	Std. Mean Difference IV, Random, 95% CI
**1.21.1 PC**								
Hagovska 2017 (1)	25.50	1.13	30	18.64	3.88	30	49.6%	2.37 [1.70; 3.04]
Nousia 2019 (2)	14.60	0.65	25	12.90	2.63	21	50.4%	0.91 [0.30; 1.52]
**Subtotal (95% CI)**			**55**			**51**	**100.0%**	**1.63 [-7.66; 10.93]**
Heterogeneity: $Tau^2 = 0.9587$; $Chi^2 = 9.96$, df = 1 (P = 0.002); $I^2 = 90\%$								
Test for overall effect: $t_{0.9990307} = 2.24$ (P = 0.268)								
**Total (95% CI)**			**55**			**51**	**100.0%**	**1.63 [-7.66; 10.93]**
Heterogeneity: $Tau^2 = 0.9587$; $Chi^2 = 9.96$, df = 1 (P = 0.002); $I^2 = 90\%$								
Test for overall effect: $t_1 = 2.24$ (P = 0.268)								
Test for subgroup differences: $Chi^2 = 0.00$, df = 0 (P = NA)								

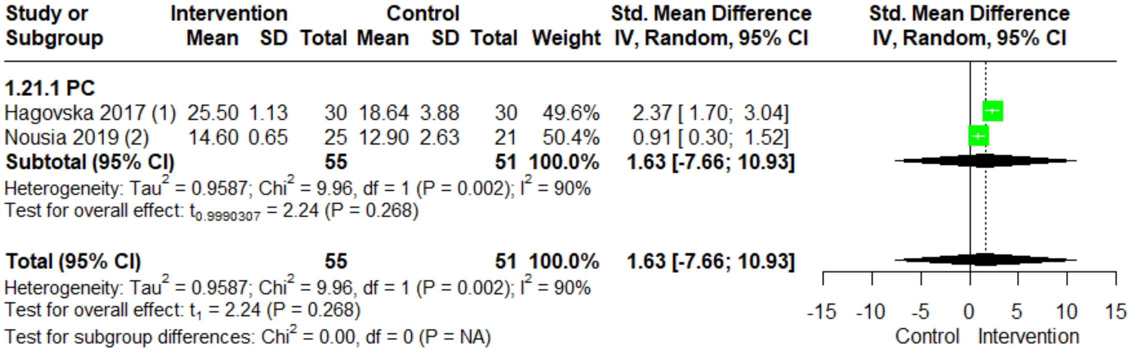

Footnote

(1) Addenbrooke's Cognitive Examination (ACE), language
(2) Boston Naming Test

Figure 23: Meta-analysis; Computer-based cognitive interventions (MCI) vs. control immediately post intervention, Outcome: **Language** (see Figure 5 in Manuscript)

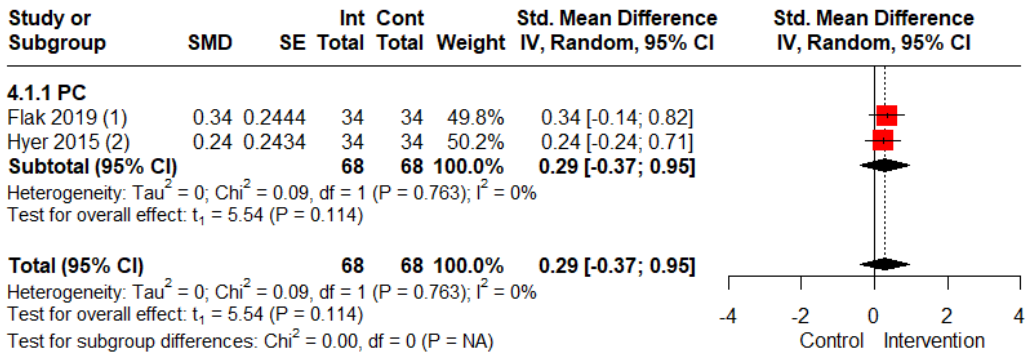

Footnote

(1) Composite score (n=14)
(2) Span Board (WMS III)

Figure 24: Meta-analysis; Computer-based cognitive interventions (MCI) vs. computer-based cognitive interventions as control immediately post intervention, Outcome: **Memory**

Study	Intervention Mean	Intervention SD	Intervention Total	Control Mean	Control SD	Control Total	Weight	Std. Mean Difference IV, Random, 95% CI
Flak 2019 (1)	15.53	8.54	34	13.19	8.01	34	7.2%	0.28 [-0.20; 0.76]
Flak 2019 (2)	34.44	7.98	34	34.47	5.84	34	7.3%	-0.00 [-0.48; 0.47]
Flak 2019 (3)	34.18	6.10	34	33.35	7.33	34	7.3%	0.12 [-0.35; 0.60]
Flak 2019 (4)	8.48	3.69	34	6.41	3.39	34	7.0%	0.58 [0.09; 1.06]
Flak 2019 (5)	37.57	12.83	34	32.00	10.18	34	7.1%	0.48 [-0.01; 0.96]
Flak 2019 (6)	13.06	5.05	34	11.73	5.38	34	7.2%	0.25 [-0.23; 0.73]
Flak 2019 (7)	7.00	3.92	34	4.97	3.61	34	7.0%	0.53 [0.05; 1.02]
Flak 2019 (8)	6.91	4.52	34	4.61	4.06	34	7.0%	0.53 [0.05; 1.01]
Flak 2019 (9)	7.56	4.60	34	4.81	4.02	34	6.9%	0.63 [0.14; 1.12]
Flak 2019 (10)	4.52	1.75	34	4.00	1.63	34	7.2%	0.30 [-0.17; 0.78]
Flak 2019 (11)	3.76	1.56	34	3.70	1.40	34	7.3%	0.04 [-0.44; 0.52]
Flak 2019 (12)	9.24	3.50	34	7.85	3.03	34	7.1%	0.42 [-0.06; 0.90]
Flak 2019 (13)	17.33	8.77	34	14.15	9.48	34	7.2%	0.34 [-0.13; 0.82]
Flak 2019 (14)	14.88	7.90	34	12.84	7.64	34	7.2%	0.26 [-0.22; 0.74]
**Total (95% CI)**			**476**			**476**	**100.0%**	**0.34 [0.22; 0.45]**

Heterogeneity: $Tau^2 = 0$; $Chi^2 = 8.55$, df = 13 ($P = 0.81$); $I^2 = 0\%$
Test for overall effect: $t_{13} = 6.35$ ($P < 0.01$)

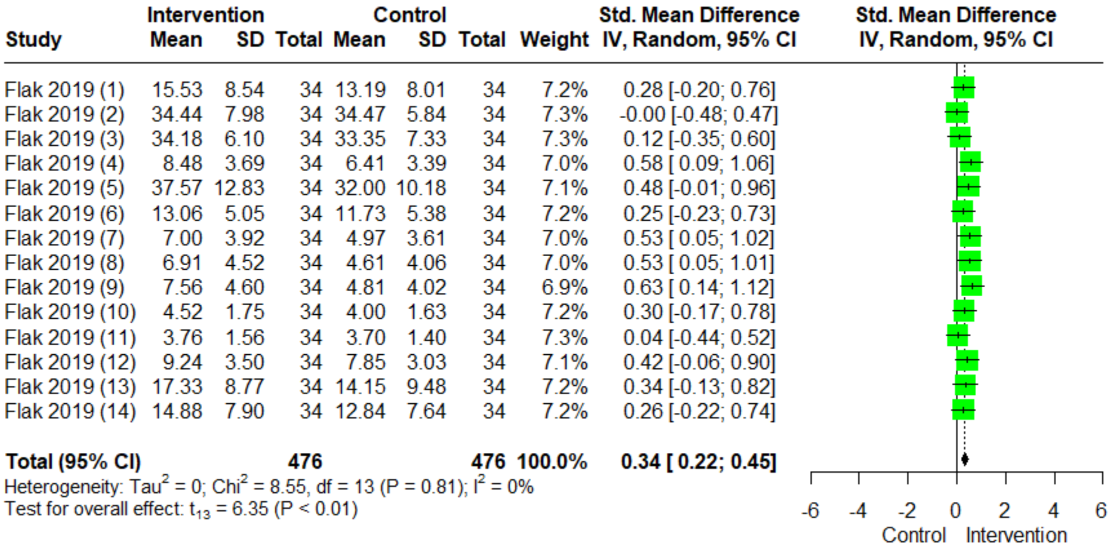

Footnote

(1) Rey-Osterreith Complex Figure, long delay recall
(2) Wechsler Memory Scale (WMS) III Faces, short delay recall
(3) Wechsler Memory Scale (WMS) III Faces, long delay recall
(4) California Verbal Learning Test (CVLT) II Short delay cued recall
(5) CVLT II, totals learned
(6) CVLT II, total hits recognition trial
(7) CVLT II, short delay free recall
(8) CVLT II, long delay free recall
(9) CVLT II, long delay cued recall
(10) CVLT II, Trial 1
(11) CVLT II, Trial B
(12) CVLT II, Trial 5
(13) WMS III, logical memory, long delay recall
(14) Rey-Osterreith Complex Figure, short delay recall

Figure 25: Composite score; Memory_Flak 2019 (see Figure 24)

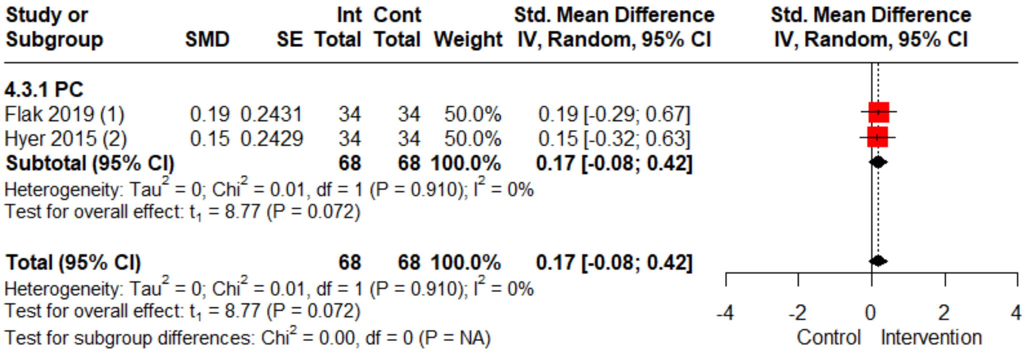

Footnote

(1) Composite score (n=5)
(2) Letter Number Sequencing (LNS)

Figure 26: Meta-analysis; Computer-based cognitive interventions (MCI) vs. computer-based cognitive interventions as control immediately post intervention, Outcome: **Working memory**

Study	Intervention Mean	SD	Total	Control Mean	SD	Total	Weight	Std. Mean Difference IV, Random, 95% CI
Flak 2019 (1)	5.50	1.05	34	5.41	1.04	34	20.1%	0.09 [-0.39; 0.56]
Flak 2019 (2)	4.24	1.25	34	4.00	0.98	34	20.0%	0.21 [-0.27; 0.69]
Flak 2019 (3)	8.38	2.69	34	7.74	2.71	34	20.0%	0.23 [-0.24; 0.71]
Flak 2019 (4)	6.82	2.24	34	6.44	1.77	34	20.0%	0.19 [-0.29; 0.66]
Flak 2019 (5)	6.85	2.21	34	6.35	1.85	34	20.0%	0.24 [-0.23; 0.72]
**Total (95% CI)**			**170**			**170**	**100.0%**	**0.19 [0.11; 0.27]**

Heterogeneity: $Tau^2 = 0$; $Chi^2 = 0.27$, df = 4 (P = 0.99); $I^2 = 0\%$

Test for overall effect: $t_4 = 6.73$ (P < 0.01)

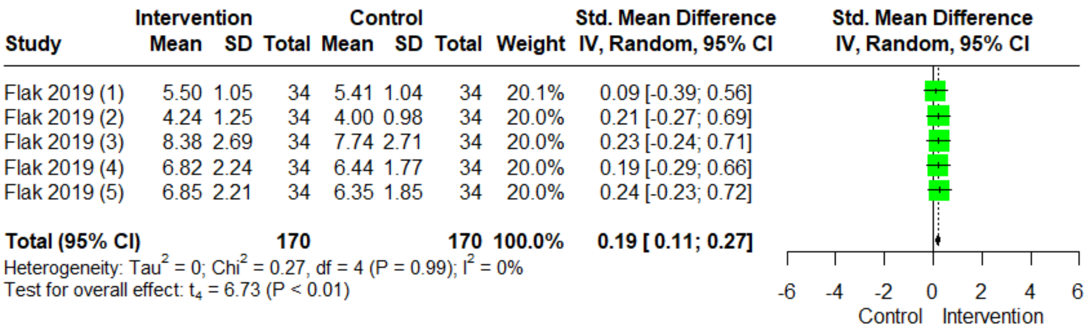

Footnote

(1) Digit Span forward
(2) Digit Span backward
(3) WMS III, letter number sequenceing
(4) WMS III, spatial span backward
(5) WMS III, spatial span forward

Figure 27: Composite score; Working memory_Flak 2019 (see Figure 26)

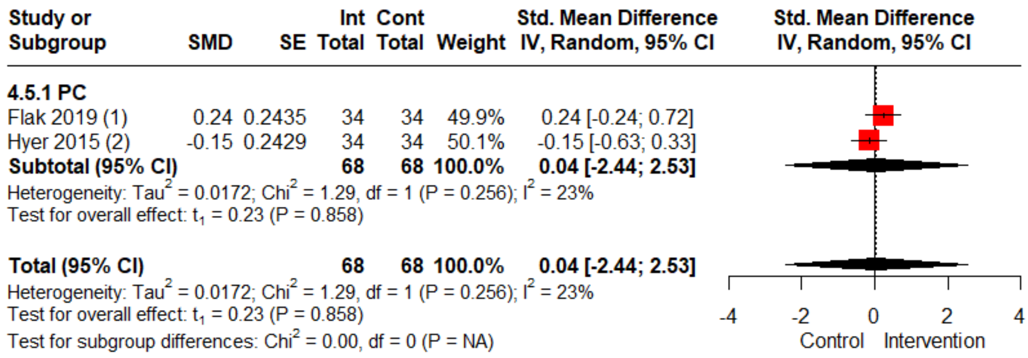

Footnote

(1) Composite score (n=5)

(2) TMT B

Figure 28: Meta-analysis; Computer-based cognitive interventions (MCI) vs. computer-based cognitive interventions as control immediately post intervention, Outcome: **Executive functioning**

Study	Intervention Mean	SD	Total	Control Mean	SD	Total	Weight	Std. Mean Difference IV, Random, 95% CI
Flak 2019 (1)	43.58	11.91	34	40.00	17.79	34	20.0%	0.23 [-0.24; 0.71]
Flak 2019 (2)	36.18	10.45	34	33.12	10.56	34	19.9%	0.29 [-0.19; 0.77]
Flak 2019 (3)	11.24	4.92	34	10.38	4.28	34	20.1%	0.18 [-0.29; 0.66]
Flak 2019 (4)	-79.67	33.12	34	-89.50	43.60	34	20.0%	0.25 [-0.23; 0.73]
Flak 2019 (5)	-96.55	39.41	34	-106.62	43.98	34	20.0%	0.24 [-0.24; 0.72]
**Total (95% CI)**			**170**			**170**	**100.0%**	**0.24 [0.19; 0.29]**

Heterogeneity: $Tau^2 = 0$; $Chi^2 = 0.09$, df = 4 (P = 1.00); $I^2 = 0\%$

Test for overall effect: $t_4 = 14.34$ (P < 0.01)

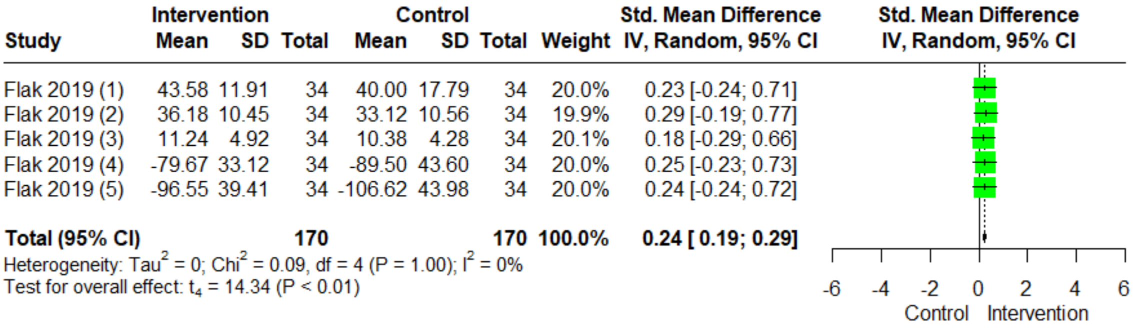

Footnote

(1) Verbal Fluency Test Letter fluency
(2) Verbal Fluency Test Category fluency
(3) Verbal Fluency Test Category Switching
(4) Delis Kaplan, Color Word Interference Test, inhibition (interference)
(5) Delis Kaplan, Color Word Interference Test, inhibition switching

Figure 29: Composite score; Executive functioning_Flak 2019 (see Figure 28)

Study or Subgroup	SMD	SE	Int Total	Cont Total	Weight	Std. Mean Difference IV, Random, 95% CI
**5.1.1 PC**						
Flak 2019 (1)	0.22	0.2433	34	34	50.5%	0.22 [-0.26; 0.70]
Hyer 2015 (2)	0.45	0.2458	34	34	49.5%	0.45 [-0.03; 0.93]
**Subtotal (95% CI)**			**68**	**68**	**100.0%**	**0.33 [-1.13; 1.80]**
Heterogeneity: $Tau^2 = 0$; $Chi^2 = 0.44$, df = 1 (P = 0.506); $I^2 = 0\%$						
Test for overall effect: $t_1 = 2.90$ (P = 0.211)						
**Total (95% CI)**			**68**	**68**	**100.0%**	**0.33 [-1.13; 1.80]**
Heterogeneity: $Tau^2 = 0$; $Chi^2 = 0.44$, df = 1 (P = 0.506); $I^2 = 0\%$						
Test for overall effect: $t_1 = 2.90$ (P = 0.211)						
Test for subgroup differences: $Chi^2 = 0.00$, df = 0 (P = NA)						

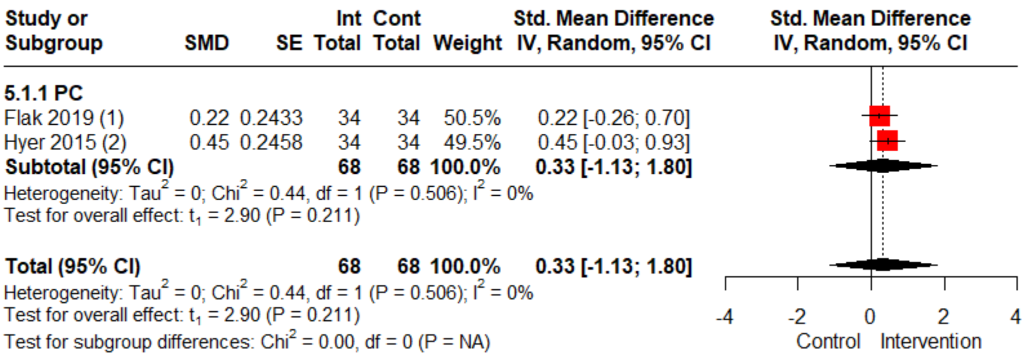

Footnote

(1) Composite score (n=14)

(2) Wechsler Memory Scale (WMS) III, span board

Figure 30: Meta-analysis; Computer-based cognitive interventions (MCI) vs. computer-based cognitive interventions follow-up, Outcome: **Memory**

Study	Intervention Mean	Intervention SD	Intervention Total	Control Mean	Control SD	Control Total	Weight	Std. Mean Difference IV, Random, 95% CI
Flak 2019 (1)	7.14	4.00	34	5.81	4.36	34	7.1%	0.31 [-0.16; 0.79]
Flak 2019 (2)	4.75	1.62	34	4.68	2.13	34	7.2%	0.04 [-0.44; 0.51]
Flak 2019 (3)	3.97	1.56	34	3.97	1.47	34	7.2%	0.00 [-0.48; 0.48]
Flak 2019 (4)	9.09	3.49	34	8.26	2.84	34	7.1%	0.26 [-0.22; 0.74]
Flak 2019 (5)	19.29	10.86	34	14.50	10.98	34	7.0%	0.43 [-0.05; 0.91]
Flak 2019 (6)	17.12	8.45	34	14.15	9.69	34	7.1%	0.32 [-0.16; 0.80]
Flak 2019 (7)	16.00	8.91	34	14.29	9.55	34	7.2%	0.18 [-0.29; 0.66]
Flak 2019 (8)	35.05	7.10	34	32.82	8.36	34	7.1%	0.28 [-0.19; 0.76]
Flak 2019 (9)	35.88	6.64	34	34.44	8.82	34	7.2%	0.18 [-0.29; 0.66]
Flak 2019 (10)	8.19	3.31	34	7.03	3.56	34	7.1%	0.33 [-0.15; 0.81]
Flak 2019 (11)	36.84	14.08	34	34.13	12.37	34	7.2%	0.20 [-0.27; 0.68]
Flak 2019 (12)	12.69	4.21	34	13.03	4.10	34	7.2%	-0.08 [-0.56; 0.39]
Flak 2019 (13)	6.91	3.88	34	5.10	3.86	34	7.0%	0.46 [-0.02; 0.94]
Flak 2019 (14)	6.34	4.72	34	5.52	4.38	34	7.2%	0.18 [-0.30; 0.65]
**Total (95% CI)**			**476**			**476**	**100.0%**	**0.22 [0.13; 0.31]**

Heterogeneity: $Tau^2 = 0$; $Chi^2 = 5.38$, df = 13 (P = 0.97); $I^2 = 0\%$
Test for overall effect: $t_{13} = 5.28$ (P < 0.01)

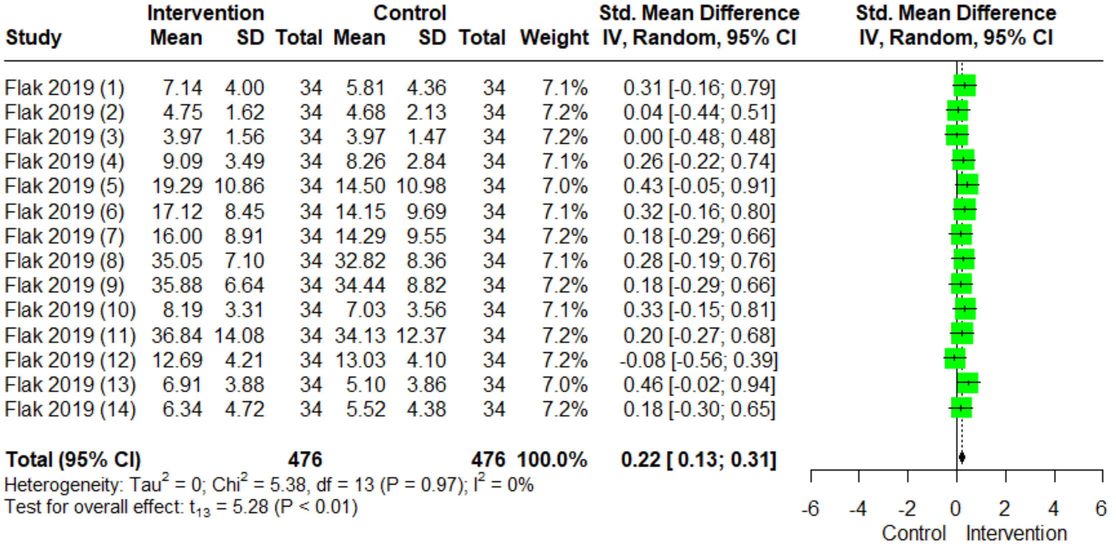

Footnote

(1) California Verbal Learning Test (CVLT) II, long delay cued recall
(2) CVLT II, Trial 1
(3) CVLT II, Trial B
(4) CVLT II, Trial 5
(5) Wechsler Memory Scale (WMS) III, logical memory, long delay recall
(6) Rey-Osterreith Complex Figure, short delay recall
(7) Rey-Osterreith Complex Figure, long delay recall
(8) WMS III, faces, long delay recall
(9) WMS III, faces, short delay recall
(10) CVLT II, short delay cued recall
(11) CVLT II, totals learned
(12) CVLT II, total hits recognition trial
(13) CVLT II, short delay free recall
(14) CVLT II, long delay free recall

Figure 31: Composite score; Memory_Flak 2019 (see Figure 30)

Study or Subgroup	SMD	SE	Int Total	Cont Total	Weight	Std. Mean Difference IV, Random, 95% CI
**5.3.1 PC**						
Flak 2019 (1)	0.30	0.2440	34	34	49.8%	0.30 [-0.18; 0.78]
Hyer 2015 (2)	0.16	0.2430	34	34	50.2%	0.16 [-0.31; 0.64]
**Subtotal (95% CI)**			**68**	**68**	**100.0%**	**0.23 [-0.63; 1.10]**
Heterogeneity: $Tau^2 = 0$; $Chi^2 = 0.16$, df = 1 (P = 0.693); $I^2 = 0\%$						
Test for overall effect: $t_1 = 3.41$ (P = 0.182)						
**Total (95% CI)**			**68**	**68**	**100.0%**	**0.23 [-0.63; 1.10]**
Heterogeneity: $Tau^2 = 0$; $Chi^2 = 0.16$, df = 1 (P = 0.693); $I^2 = 0\%$						
Test for overall effect: $t_1 = 3.41$ (P = 0.182)						
Test for subgroup differences: $Chi^2 = 0.00$, df = 0 (P = NA)						

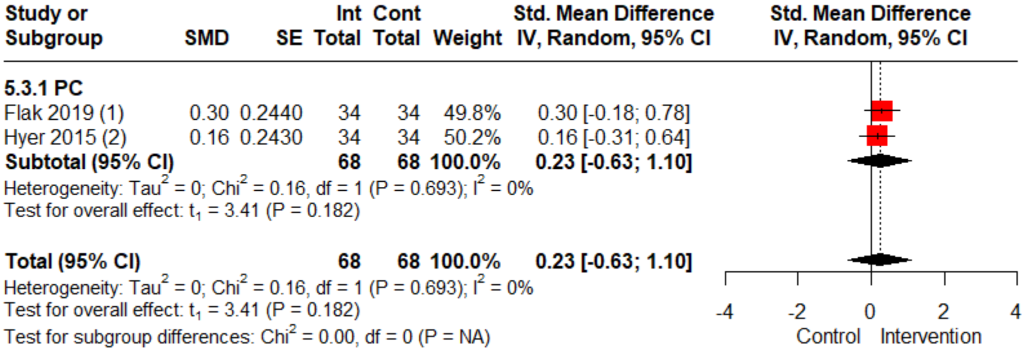

Footnote

(1) Composite score (n=5)

(2) Letter Number Sequencing (LNS)

Figure 32: Meta-analysis; Computer-based cognitive interventions (MCI) vs. computer-based cognitive interventions follow-up, Outcome: **Working memory**

Study	Intervention Mean	Intervention SD	Intervention Total	Control Mean	Control SD	Control Total	Weight	Std. Mean Difference IV, Random, 95% CI
Flak 2019 (1)	5.35	1.07	34	5.32	1.09	34	20.3%	0.03 [-0.45; 0.50]
Flak 2019 (2)	4.24	1.23	34	3.79	1.20	34	20.0%	0.37 [-0.11; 0.85]
Flak 2019 (3)	8.18	2.88	34	7.26	3.32	34	20.1%	0.29 [-0.19; 0.77]
Flak 2019 (4)	6.29	1.81	34	5.82	1.89	34	20.1%	0.25 [-0.23; 0.73]
Flak 2019 (5)	7.38	1.90	34	6.38	1.68	34	19.5%	0.55 [0.07; 1.04]
**Total (95% CI)**			**170**			**170**	**100.0%**	**0.30 [0.06; 0.53]**

Heterogeneity: $Tau^2 = 0$; $Chi^2 = 2.41$, df = 4 (P = 0.66); $I^2 = 0\%$
Test for overall effect: $t_4 = 3.49$ (P = 0.03)

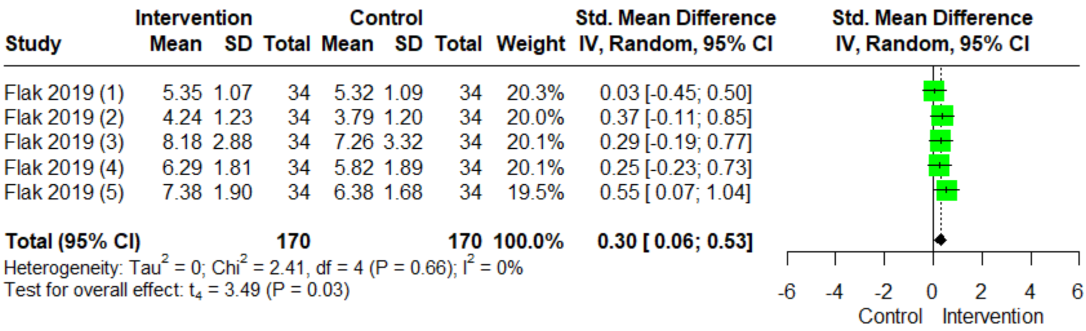

Footnote

(1) Digit Span forward
(2) Digit Span backward
(3) Wechsler Memory Scale (WMS) III, Letter-number sequencing
(4) WMS III, Spatial Span Backward
(5) Spatial span forward

Figure 33: Composite score; Working memory_Flak 2019 (see Figure 32)

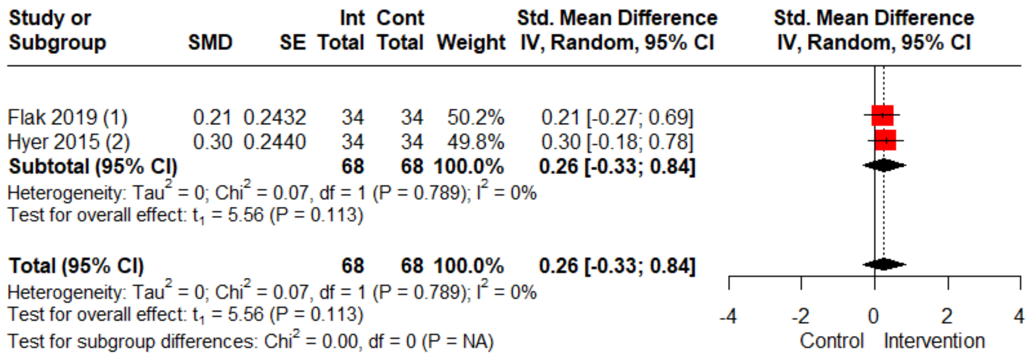

Footnote

(1) Composite score (n=5)
(2) TMT B

Figure 34: Meta-analysis; Computer-based cognitive interventions (MCI) vs. computer-based cognitive interventions follow-up, Outcome: **Executive functioning**

Study	Intervention Mean	Intervention SD	Intervention Total	Control Mean	Control SD	Control Total	Weight	Std. Mean Difference IV, Random, 95% CI
Flak 2019 (1)	42.61	11.39	34	39.06	16.59	34	20.0%	0.25 [-0.23; 0.72]
Flak 2019 (2)	36.21	12.04	34	32.50	10.55	34	19.9%	0.32 [-0.15; 0.80]
Flak 2019 (3)	11.13	4.30	34	10.74	4.64	34	20.1%	0.09 [-0.39; 0.56]
Flak 2019 (4)	-80.09	31.38	34	-82.85	40.47	34	20.1%	0.08 [-0.40; 0.55]
Flak 2019 (5)	-93.48	38.22	34	-107.74	43.41	34	19.8%	0.34 [-0.13; 0.82]
**Total (95% CI)**			**170**			**170**	**100.0%**	**0.21 [0.06; 0.37]**

Heterogeneity: $Tau^2 = 0$; $Chi^2 = 1.11$, df = 4 (P = 0.89); $I^2 = 0\%$
Test for overall effect: $t_4 = 3.74$ (P = 0.02)

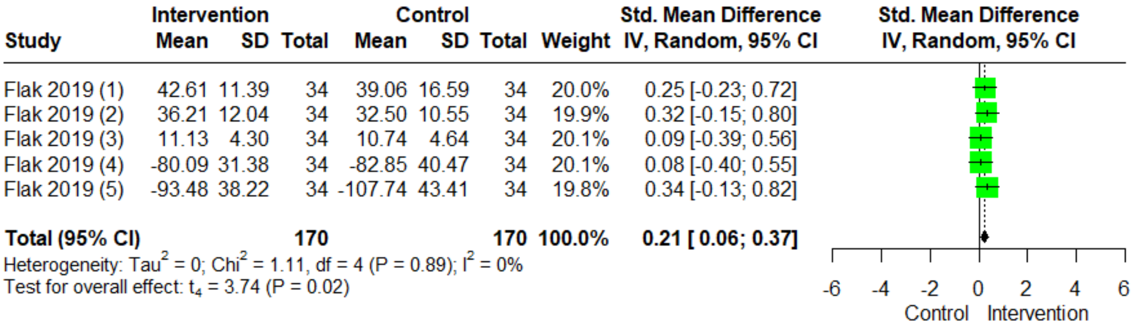

Footnote

(1) Verbal fluency test, letter fluency
(2) Delis Kaplan, Verbal fluency test, category fluency
(3) Delis Kaplan, Verbal fluency test, category switching
(4) Delis Kaplan, Color Word Interference Test, inhibition (interference)
(5) Delis Kaplan, Color Word Interference Test, inhibition switching

Figure 35: Composite score; Executive functioning_Flak 2019 (see Figure 34)

Study or Subgroup	Intervention Mean	Intervention SD	Intervention Total	Control Mean	Control SD	Control Total	Weight	Std. Mean Difference IV, Random, 95% CI
**2.1.1 PC**								
Cinar 2020 (1)	23.23	3.68	30	16.17	3.94	30	0.0%	1.83 [1.22; 2.44]
Galante 2007 (2)	23.00	3.90	7	24.90	2.80	4	17.1%	-0.49 [-1.74; 0.77]
Heiss 1994 (3)	19.33	6.82	18	19.29	5.89	17	61.2%	0.01 [-0.66; 0.67]
**Subtotal (95% CI)**			**55**			**51**	**78.3%**	**-0.10 [-2.69; 2.48]**
Heterogeneity: $Tau^2 = 0$; $Chi^2 = 0.46$, df = 1 (P = 0.496); $I^2 = 0\%$								
Test for overall effect: $t_1 = -0.50$ (P = 0.706)								
**2.1.2 Tablet**								
Lee 2013 (4)	19.67	5.20	7	17.29	3.15	6	21.7%	0.50 [-0.61; 1.62]
**Subtotal (95% CI)**			**7**			**6**	**21.7%**	**0.50 [-0.61; 1.62]**
Heterogeneity: not applicable								
Test for overall effect: $t_{Inf} = 0.89$ (P = 0.375)								
**Total (95% CI)**			**62**			**57**	**100.0%**	**0.03 [-0.91; 0.97]**
Heterogeneity: $Tau^2 = 0$; $Chi^2 = 1.35$, df = 2 (P = 0.509); $I^2 = 0\%$								
Test for overall effect: $t_2 = 0.14$ (P = 0.904)								
Test for subgroup differences: $Chi^2 = 1.01$, df = 1 (P = 0.316)								

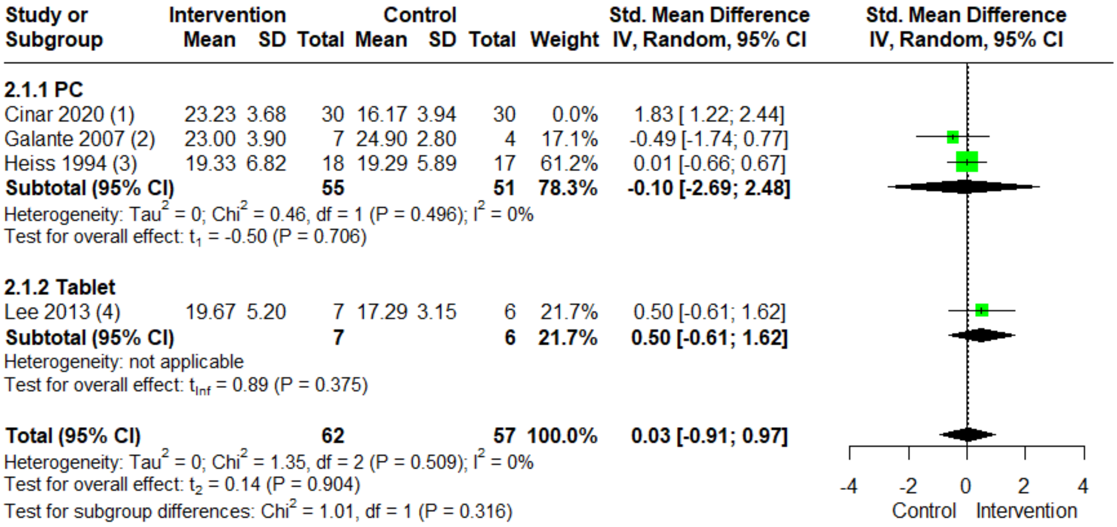

Footnote

(1) Montreal Cognitive Assessment (MoCA)
(2) Mini Mental State Examination (MMSE)
(3) MMSE; intervention group 1 (IG1) vs. control group
(4) MMSE

Figure 36: Sensitivity meta-analysis; Computer-based cognitive interventions without Cinar 2020 (large SMD) (DEMENTIA) vs. control immediately post intervention, Outcome: **Global cognition**

Study or Subgroup	SMD	SE	Int Total	Cont Total	Weight	Std. Mean Difference IV, Random, 95% CI
**3.4.1 PC**						
Galante 2007 (1)	-0.45	0.6381	7	4	43.4%	-0.45 [-1.70; 0.80]
**Subtotal (95% CI)**			**7**	**4**	**43.4%**	**-0.45 [-1.70; 0.80]**
Heterogeneity: not applicable						
Test for overall effect: t_{Inf} = -0.71 (P = 0.481)						
**3.4.2 Tablet**						
Lee 2013 (2)	0.24	0.5592	7	6	56.6%	0.24 [-0.86; 1.33]
**Subtotal (95% CI)**			**7**	**6**	**56.6%**	**0.24 [-0.86; 1.33]**
Heterogeneity: not applicable						
Test for overall effect: t_{Inf} = 0.43 (P = 0.670)						
**Total (95% CI)**			**14**	**10**	**100.0%**	**-0.06 [-4.40; 4.28]**
Heterogeneity: Tau^2 = 0; Chi^2 = 0.66, df = 1 (P = 0.417); I^2 = 0%						
Test for overall effect: t_1 = -0.18 (P = 0.888)						
Test for subgroup differences: Chi^2 = 0.66, df = 1 (P = 0.417)						

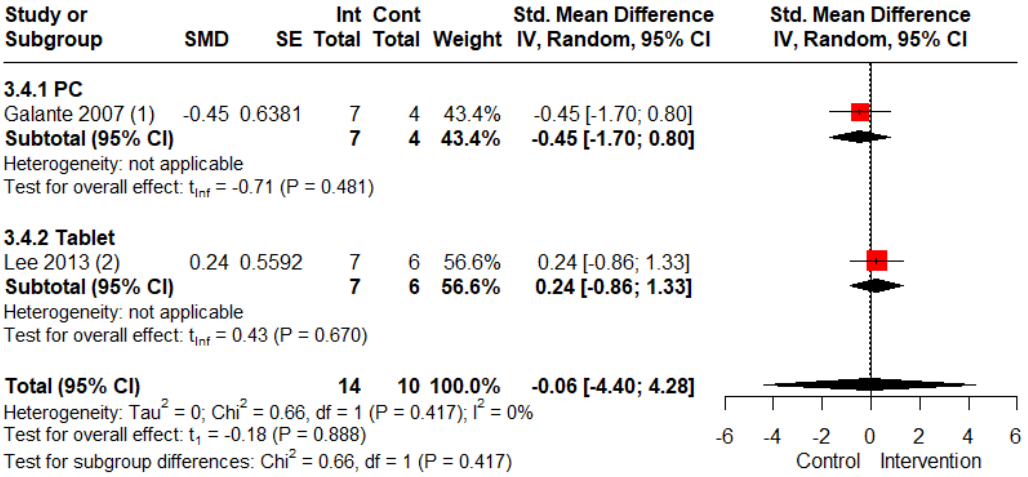

Footnote

(1) Composite score (n=2)
(2) Mini Mental State Examination (MMSE)

Figure 37: Meta-analysis; Computer-based cognitive interventions (DEMENTIA) vs. control follow-up (3 months), Outcome: **Global cognition**

Study	Intervention Mean	SD	Total	Control Mean	SD	Total	Weight	Std. Mean Difference IV, Random, 95% CI
Galante 2007 (1)	21.60	4.60	7	24.40	2.30	4	48.6%	-0.64 [-1.91; 0.63]
Galante 2007 (2)	83.10	10.00	7	85.70	4.80	4	51.4%	-0.28 [-1.51; 0.96]
**Total (95% CI)**			**14**			**8**	**100.0%**	**-0.45 [-2.78; 1.88]**

Heterogeneity: $Tau^2 = 0$; $Chi^2 = 0.16$, df = 1 (P = 0.69); $I^2 = 0\%$
Test for overall effect: $t_1 = -2.48$ (P = 0.24)

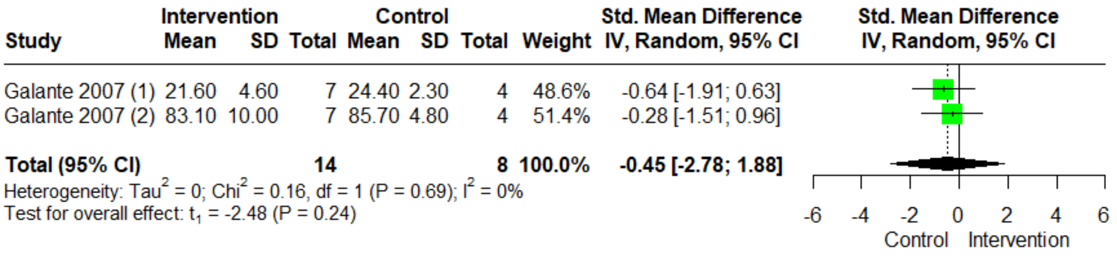

Footnote

(1) Mini Mental State Examination (MMSE)
(2) Milan Overall Dementia Assessment (MODA)

Figure 38: Composite score; Global cognition_Galante 2007 (see Figure 37)

Study	Intervention Mean	Intervention SD	Intervention Total	Control Mean	Control SD	Control Total	Weight	Std. Mean Difference IV, Random, 95% CI
Heiss 1994 (1)	7.25	1.00	18	7.05	1.39	17	50.0%	0.16 [-0.50; 0.83]
Heiss 1994 (2)	-4.06	5.63	18	-3.29	4.35	17	50.0%	-0.15 [-0.81; 0.51]
**Total (95% CI)**			**36**			**34**	**100.0%**	**0.01 [-1.97; 1.98]**

Heterogeneity: $Tau^2 = 0$; $Chi^2 = 0.42$, df = 1 (P = 0.52); $I^2 = 0\%$
Test for overall effect: $t_1 = 0.04$ (P = 0.97)

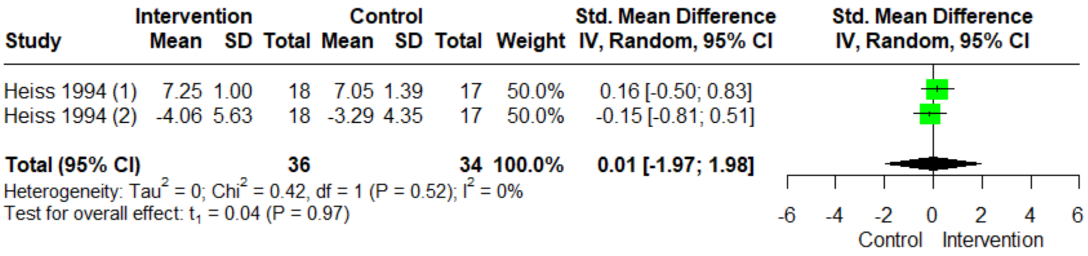

Footnote

(1) Selective reminding test/task - Recognition (hits)
(2) Selective reminding test/task - Recognition (false-positive)

Figure 39: Composite score; Memory_Heiss 1994 (see Figure 7 in Manuscript)

Study	Intervention Mean	Intervention SD	Intervention Total	Control Mean	Control SD	Control Total	Weight	Std. Mean Difference IV, Random, 95% CI
Lee 2013 (1)	-1.07	0.13	7	-1.18	0.16	6	48.2%	0.71 [-0.43; 1.85]
Lee 2013 (2)	10.67	4.97	7	9.43	2.94	6	51.8%	0.28 [-0.82; 1.37]
**Total (95% CI)**			**14**			**12**	**100.0%**	**0.48 [-2.26; 3.23]**

Heterogeneity: $Tau^2 = 0$; $Chi^2 = 0.29$, df = 1 (P = 0.59); $I^2 = 0\%$
Test for overall effect: $t_1 = 2.25$ (P = 0.27)

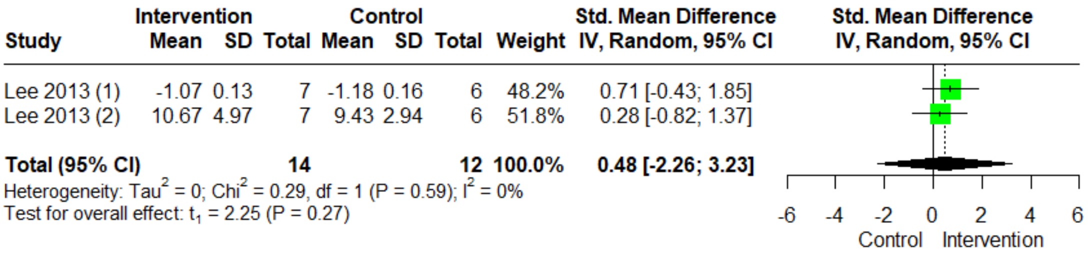

Footnote

(1) Brief Assessment of Prospective Memory-Short Form (BAPM)
(2) Hong Kong List Learning Test (HKLLT)

Figure 40: Composite score; Memory_Lee 2013 (see Figure 7 in Manuscript)

Study	Intervention Mean	Intervention SD	Intervention Total	Control Mean	Control SD	Control Total	Weight	Std. Mean Difference IV, Random, 95% CI
Karssemeijer 2019 (1)	-16.90	11.20	38	-20.00	10.90	39	50.6%	0.28 [-0.17; 0.73]
Karssemeijer 2019 (2)	-85.50	41.70	38	-107.10	41.70	39	49.4%	0.51 [0.06; 0.97]
**Total (95% CI)**			**76**			**78**	**100.0%**	**0.39 [-1.10; 1.89]**

Heterogeneity: $Tau^2 = 0$; $Chi^2 = 0.52$, df = 1 (P = 0.47); $I^2 = 0\%$
Test for overall effect: $t_1 = 3.35$ (P = 0.18)

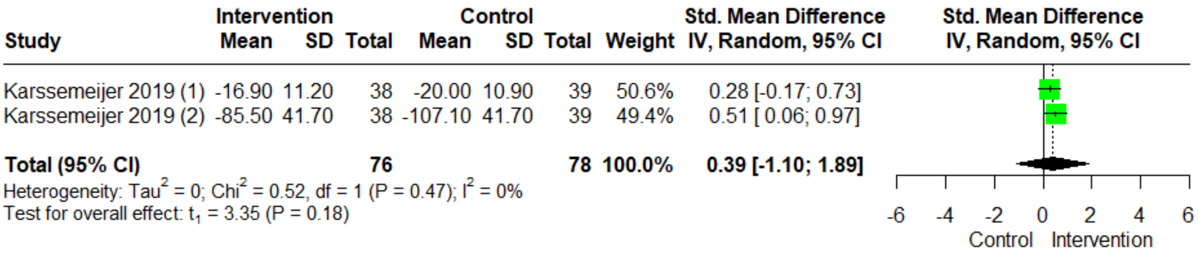

Footnote

(1) Location learning test (revised) – displacement score delayed recall
(2) Location learning test (revised) – displacement score trial 1-5

Figure 41: Composite score; Memory_Karssemeijer 2019 (see Figure 7 in Manuscript)

Study or Subgroup	SMD	SE	Int Total	Cont Total	Weight	Std. Mean Difference IV, Random, 95% CI
**2.6.1 PC**						
Galante 2007 (1)	0.18	0.6286	7	4	8.3%	0.18 [-1.05; 1.41]
Heiss 1994 (2)	-0.30	0.3404	18	17	28.4%	-0.30 [-0.97; 0.36]
**Subtotal (95% CI)**			**25**	**21**	**36.8%**	**-0.19 [-2.77; 2.38]**
Heterogeneity: $Tau^2 = 0$; $Chi^2 = 0.46$, df = 1 (P = 0.499); $I^2 = 0\%$						
Test for overall effect: $t_1 = -0.95$ (P = 0.515)						
**2.6.2 VR**						
Karssemeijer 2019 (3)	-0.15	0.2283	38	39	63.2%	-0.15 [-0.60; 0.30]
**Subtotal (95% CI)**			**38**	**39**	**63.2%**	**-0.15 [-0.60; 0.30]**
Heterogeneity: not applicable						
Test for overall effect: $t_{Inf} = -0.66$ (P = 0.511)						
**Total (95% CI)**			**63**	**60**	**100.0%**	**-0.17 [-0.54; 0.21]**
Heterogeneity: $Tau^2 = 0$; $Chi^2 = 0.47$, df = 2 (P = 0.790); $I^2 = 0\%$						
Test for overall effect: $t_2 = -1.88$ (P = 0.201)						
Test for subgroup differences: $Chi^2 = 0.02$, df = 1 (P = 0.888)						

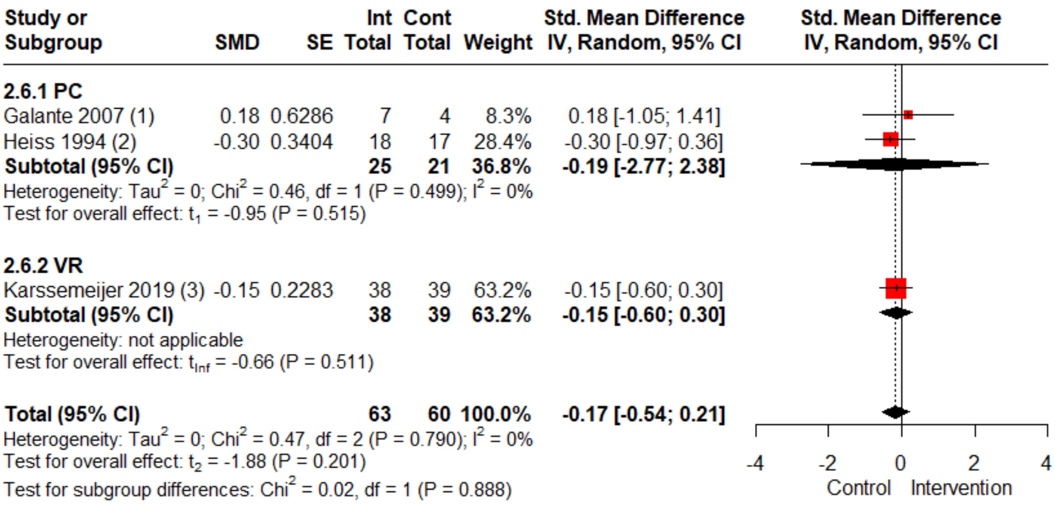

Footnote

(1) Corsi's block tapping test
(2) Corsi's block tapping test
(3) Composite score (n=2)

Figure 42: Meta-analysis; Computer-based cognitive interventions (DEMENTIA) vs. control immediately post intervention, Outcome: **Working memory** (see Figure 8 in Manuscript)

Study	Intervention Mean	Intervention SD	Intervention Total	Control Mean	Control SD	Control Total	Weight	Std. Mean Difference IV, Random, 95% CI
Karssemeijer 2019 (1)	8.30	3.60	38	8.70	3.60	39	50.1%	-0.11 [-0.56; 0.34]
Karssemeijer 2019 (2)	10.10	2.90	38	10.80	4.00	39	49.9%	-0.20 [-0.65; 0.25]
**Total (95% CI)**			**76**			**78**	**100.0%**	**-0.15 [-0.71; 0.40]**

Heterogeneity: $Tau^2 = 0$; $Chi^2 = 0.07$, df = 1 (P = 0.79); $I^2 = 0\%$
Test for overall effect: $t_1 = -3.50$ (P = 0.18)

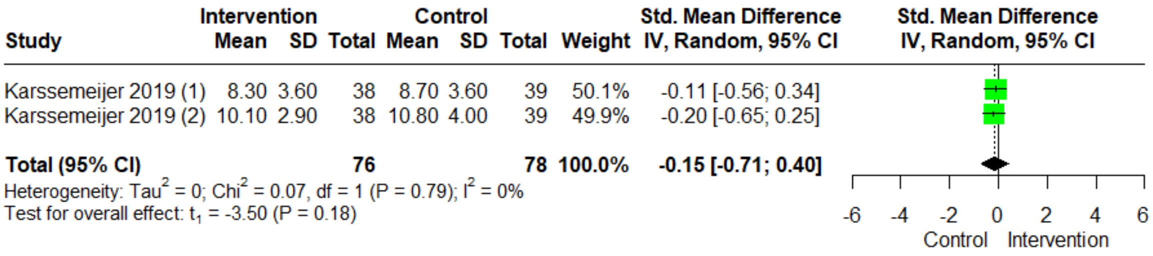

Footnote
(1) Spatial Span
(2) Digit span (WAIS-III)

Figure 43: Composite score; Working memory_Karssemeijer 2019 (see Figure 42)

Study or Subgroup	SMD	SE	Int Total	Cont Total	Weight	Std. Mean Difference IV, Random, 95% CI
**2.8.1 PC**						
Galante 2007 (1)	-0.33	0.6328	7	4	8.3%	-0.33 [-1.57; 0.91]
Heiss 1994 (2)	0.21	0.3393	18	17	28.7%	0.21 [-0.45; 0.88]
**Subtotal (95% CI)**			**25**	**21**	**37.0%**	**0.09 [-2.76; 2.94]**
Heterogeneity: $Tau^2 = 0$; $Chi^2 = 0.56$, df = 1 (P = 0.453); $I^2 = 0\%$						
Test for overall effect: $t_1 = 0.41$ (P = 0.752)						
**2.8.2 VR**						
Karssemeijer 2019 (3)	0.29	0.2292	38	39	63.0%	0.29 [-0.16; 0.74]
**Subtotal (95% CI)**			**38**	**39**	**63.0%**	**0.29 [-0.16; 0.74]**
Heterogeneity: not applicable						
Test for overall effect: $t_{Inf} = 1.27$ (P = 0.206)						
**Total (95% CI)**			**63**	**60**	**100.0%**	**0.22 [-0.29; 0.72]**
Heterogeneity: $Tau^2 = 0$; $Chi^2 = 0.84$, df = 2 (P = 0.657); $I^2 = 0\%$						
Test for overall effect: $t_2 = 1.84$ (P = 0.207)						
Test for subgroup differences: $Chi^2 = 0.38$, df = 1 (P = 0.537)						

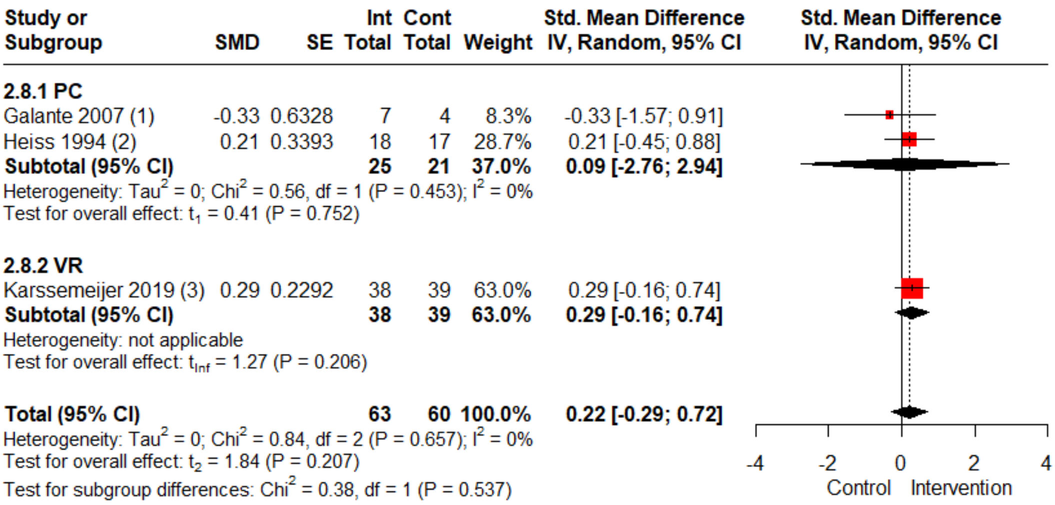

Footnote

(1) Digit cancellation test
(2) Alters-Konzentrationstest (AKT) (t)
(3) Composite score (n=2)

Figure 44: Meta-analysis; Computer-based cognitive interventions (DEMENTIA) vs. control immediately post intervention, Outcome: **Attention/concentration/processing speed** (see Figure 8 in Manuscript)

Study	Intervention Mean	Intervention SD	Intervention Total	Control Mean	Control SD	Control Total	Weight	Std. Mean Difference IV, Random, 95% CI
Karssemeijer 2019 (1)	-50.30	42.00	38	-63.60	61.00	39	50.1%	0.25 [-0.20; 0.70]
Karssemeijer 2019 (2)	-49.40	17.90	38	-56.70	25.90	39	49.9%	0.32 [-0.13; 0.77]
**Total (95% CI)**			**76**			**78**	**100.0%**	**0.29 [-0.18; 0.75]**

Heterogeneity: $Tau^2 = 0$; $Chi^2 = 0.05$, df = 1 (P = 0.82); $I^2 = 0\%$
Test for overall effect: $t_1 = 7.87$ (P = 0.08)

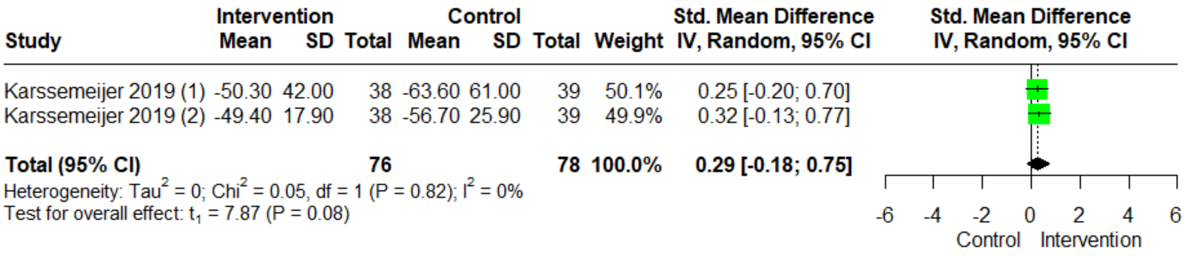

Footnote
(1) TMT A
(2) Stroop Color-Word Test (SCWT) – word reading (sec)

Figure 45: Composite score; Attention/concentration/processing speed_Karssemeijer 2019 (see Figure 44)

Study or Subgroup	SMD	SE	Int Total	Cont Total	Weight	Std. Mean Difference IV, Random, 95% CI	Std. Mean Difference IV, Random, 95% CI
**2.10.1 PC**							
Galante 2007 (1)	0.06	0.6270	7	4	8.4%	0.06 [-1.17; 1.29]	
Heiss 1994 (2)	0.30	0.3403	18	17	28.4%	0.30 [-0.37; 0.97]	
**Subtotal (95% CI)**			**25**	**21**	**36.8%**	**0.25 [-1.03; 1.52]**	
Heterogeneity: $Tau^2 = 0$; $Chi^2 = 0.11$, df = 1 (P = 0.737); $I^2 = 0\%$							
Test for overall effect: $t_1 = 2.44$ (P = 0.248)							
**2.10.2 VR**							
Karssemeijer 2019 (3)	0.08	0.2280	38	39	63.2%	0.08 [-0.37; 0.53]	
**Subtotal (95% CI)**			**38**	**39**	**63.2%**	**0.08 [-0.37; 0.53]**	
Heterogeneity: not applicable							
Test for overall effect: $t_{Inf} = 0.35$ (P = 0.726)							
**Total (95% CI)**			**63**	**60**	**100.0%**	**0.14 [-0.16; 0.45]**	
Heterogeneity: $Tau^2 = 0$; $Chi^2 = 0.31$, df = 2 (P = 0.858); $I^2 = 0\%$							
Test for overall effect: $t_2 = 1.98$ (P = 0.186)							
Test for subgroup differences: $Chi^2 = 0.44$, df = 1 (P = 0.507)							

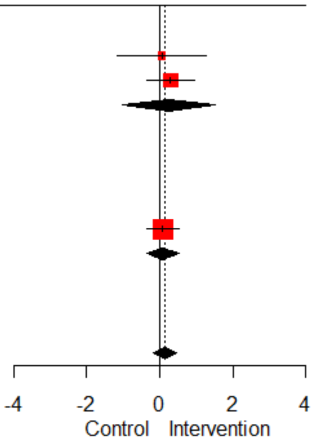

Footnote

(1) Composite score (n=2)
(2) Composite score (n=2)
(3) Composite score (n=3)

Figure 46: Meta-analysis; Computer-based cognitive interventions (DEMENTIA) vs. control immediately post intervention, Outcome: **Executive functioning** (see Figure 8 in Manuscript)

Study	Intervention Mean	SD	Total	Control Mean	SD	Total	Weight	Std. Mean Difference IV, Random, 95% CI
Galante 2007 (1)	11.90	4.70	7	12.40	5.10	4	50.2%	-0.09 [-1.32; 1.13]
Galante 2007 (2)	33.10	9.20	7	30.80	9.70	4	49.8%	0.22 [-1.01; 1.46]
**Total (95% CI)**			**14**			**8**	**100.0%**	**0.06 [-1.96; 2.09]**

Heterogeneity: $Tau^2 = 0$; $Chi^2 = 0.13$, df = 1 (P = 0.72); $I^2 = 0\%$
Test for overall effect: $t_1 = 0.40$ (P = 0.76)

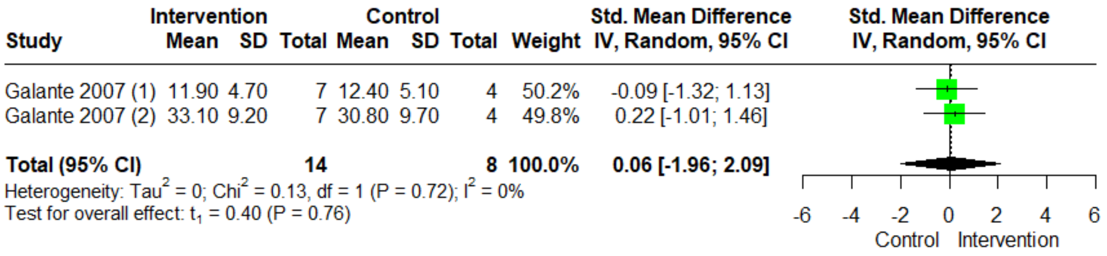

Footnote

(1) Semantic verbal fluency
(2) Phonemic verbal fluency

Figure 47: Composite score; Executive functioning_Galante 2007 (see Figure 46)

Study	Intervention Mean	Intervention SD	Intervention Total	Control Mean	Control SD	Control Total	Weight	Std. Mean Difference IV, Random, 95% CI
Heiss 1994 (1)	-0.67	0.12	18	-0.69	0.16	17	50.7%	0.14 [-0.52; 0.80]
Heiss 1994 (2)	12.43	5.84	18	9.81	5.21	17	49.3%	0.46 [-0.21; 1.13]
**Total (95% CI)**			**36**			**34**	**100.0%**	**0.30 [-1.75; 2.35]**

Heterogeneity: $Tau^2 = 0$; $Chi^2 = 0.45$, df = 1 (P = 0.50); $I^2 = 0\%$
Test for overall effect: $t_1 = 1.85$ (P = 0.32)

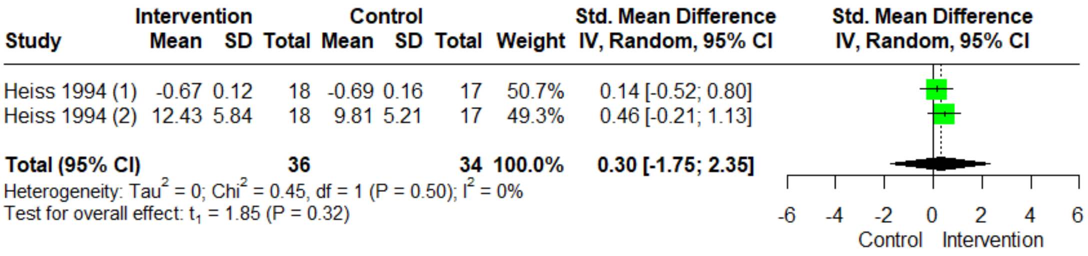

Footnote

(1) Go/no go reaction time
(2) Verbal fluency (Supermarket)

Figure 48: Composite score; Executive functioning_Heiss 1994 (see Figure 46)

Study	Intervention Mean	Intervention SD	Intervention Total	Control Mean	Control SD	Control Total	Weight	Std. Mean Difference IV, Random, 95% CI
Karssemeijer 2019 (1)	-8.30	6.40	38	-8.80	6.20	39	33.3%	0.08 [-0.37; 0.53]
Karssemeijer 2019 (2)	-160.70	106.20	38	-170.10	106.80	39	33.3%	0.09 [-0.36; 0.53]
Karssemeijer 2019 (3)	20.90	10.60	38	20.20	12.00	39	33.3%	0.06 [-0.39; 0.51]
**Total (95% CI)**			**114**			**117**	**100.0%**	**0.08 [0.04; 0.11]**

Heterogeneity: $Tau^2 = 0$; $Chi^2 = 0.01$, df = 2 (P = 1.00); $I^2 = 0\%$
Test for overall effect: $t_2 = 9.83$ (P = 0.01)

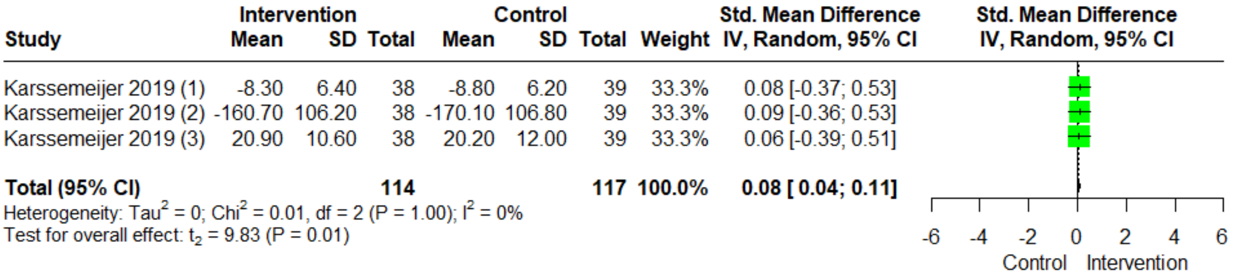

Footnote

(1) Rule Shift Cards Test, errors
(2) TMT B
(3) Verbal fluency test, letter fluency

Figure 49: Composite score; Executive functioning_Karssemeijer 2019 (see Figure 46)

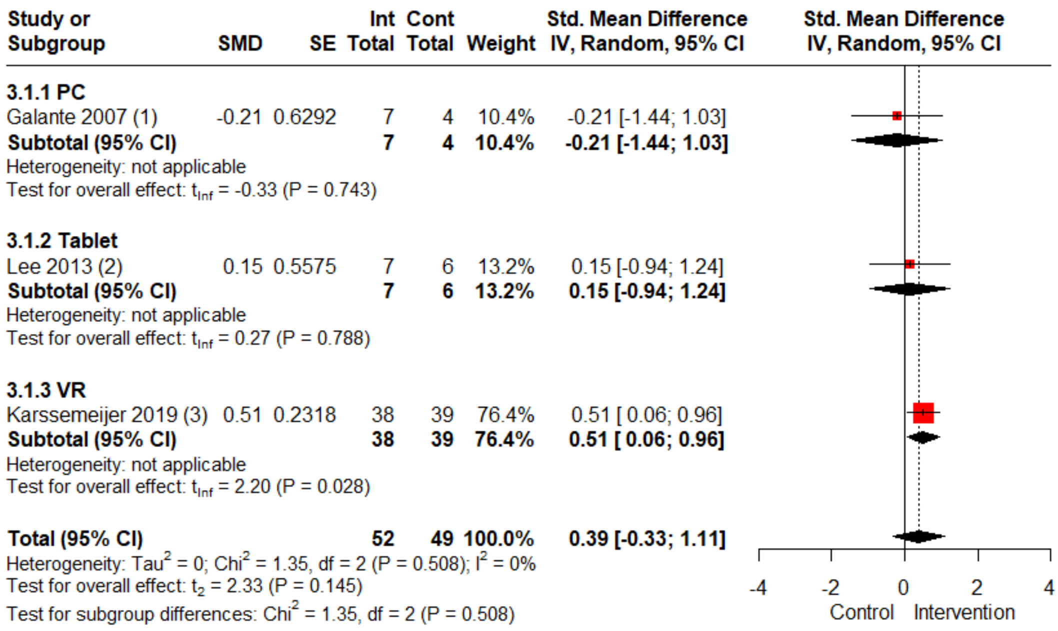

Footnote

(1) Prose memory
(2) Composite score (n=2)
(3) Composite score (n=2)

Figure 50: Meta-analysis; Computer-based cognitive interventions (DEMENTIA) vs. control follow-up (3 months), Outcome: **Memory**

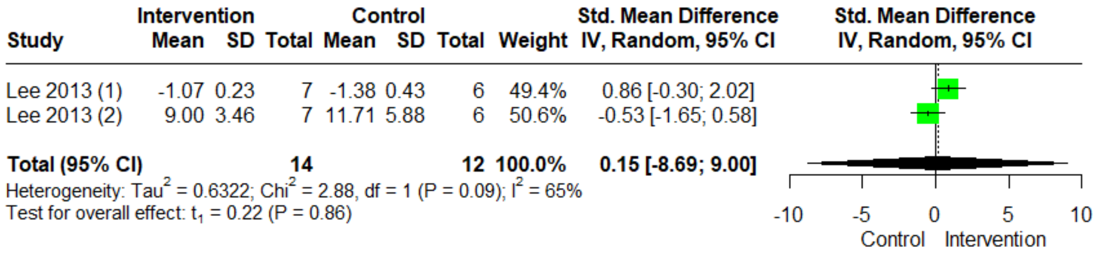

Footnote

(1) Brief Assessment of Prospective Memory-Short Form (BAPM)

(2) Hong Kong List Learning Test (HKLLT)

Figure 51: Composite score; Memory_Lee 2013 (see Figure 50)

Study	Intervention Mean	Intervention SD	Intervention Total	Control Mean	Control SD	Control Total	Weight	Std. Mean Difference IV, Random, 95% CI
Karssemeijer 2019 (1)	-18.50	8.10	38	-23.30	10.80	39	50.1%	0.50 [0.04; 0.95]
Karssemeijer 2019 (2)	-96.10	34.10	38	-118.80	51.20	39	49.9%	0.52 [0.06; 0.97]
**Total (95% CI)**			**76**			**78**	**100.0%**	**0.51 [0.39; 0.62]**

Heterogeneity: $Tau^2 = 0$; $Chi^2 = 0.00$, df = 1 (P = 0.96); $I^2 = 0\%$
Test for overall effect: $t_1 = 54.89$ (P = 0.01)

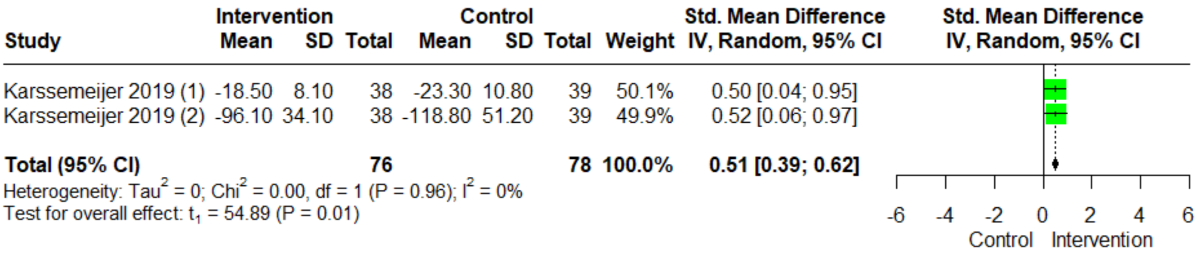

Footnote

(1) Location learning test (revised) – displacement score delayed recall
(2) Location learning test (revised) – displacement score trial 1-5

Figure 52: Composite score; Memory_Karssemeijer 2019 (see Figure 50)

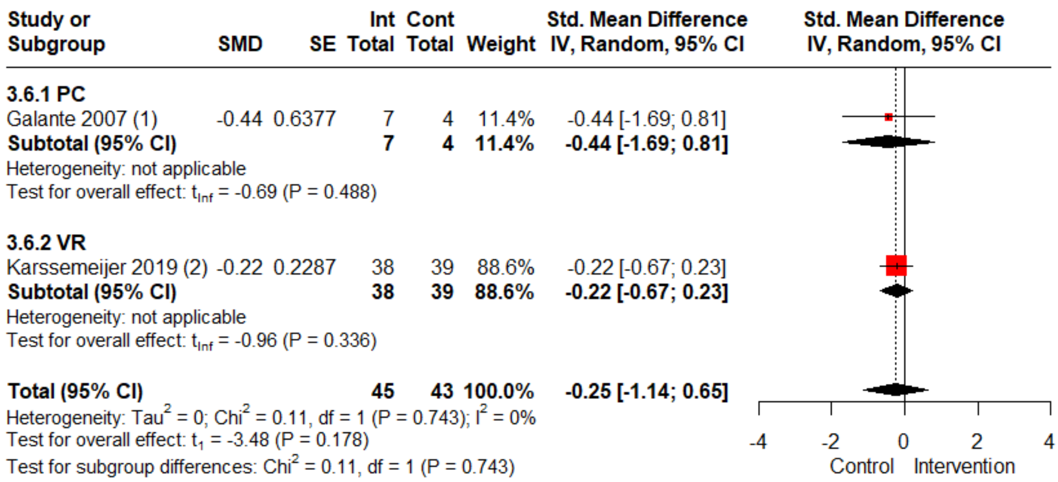

Footnote

(1) Corsi tapping test

(2) Composite score (n=2)

Figure 53: Meta-analysis; Computer-based cognitive interventions (DEMENTIA) vs. control follow-up (3 months), Outcome: **Working memory**

Study	Intervention Mean	Intervention SD	Intervention Total	Control Mean	Control SD	Control Total	Weight	Std. Mean Difference IV, Random, 95% CI
Karssemeijer 2019 (1)	7.70	3.80	38	9.30	4.20	39	49.5%	-0.40 [-0.85; 0.06]
Karssemeijer 2019 (2)	9.60	3.30	38	9.80	3.60	39	50.5%	-0.06 [-0.50; 0.39]
**Total (95% CI)**			**76**			**78**	**100.0%**	**-0.22 [-2.37; 1.92]**

Heterogeneity: $Tau^2 = 0.0046$; $Chi^2 = 1.09$, df = 1 (P = 0.30); $I^2 = 8\%$
Test for overall effect: $t_1 = -1.33$ (P = 0.41)

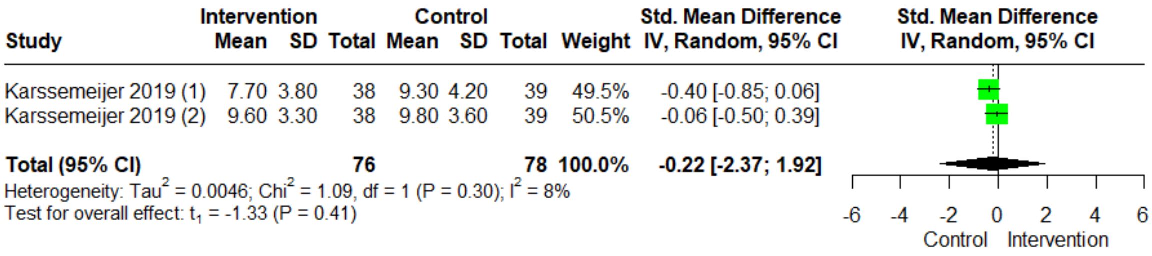

Footnote

(1) Spatial Span

(2) Digit span (WAIS-III)

Figure 54: Composite score; Working memory_Karssemeijer 2019 (see Figure 53)

Study or Subgroup	SMD	SE	Int Total	Cont Total	Weight	Std. Mean Difference IV, Random, 95% CI
**3.8.1 PC**						
Galante 2007 (1)	-0.22	0.6296	7	4	11.6%	-0.22 [-1.46; 1.01]
**Subtotal (95% CI)**			**7**	**4**	**11.6%**	**-0.22 [-1.46; 1.01]**
Heterogeneity: not applicable						
Test for overall effect: t_{Inf} = -0.35 (P = 0.723)						
**3.8.2 VR**						
Karssemeijer 2019 (2)	0.20	0.2285	38	39	88.4%	0.20 [-0.25; 0.65]
**Subtotal (95% CI)**			**38**	**39**	**88.4%**	**0.20 [-0.25; 0.65]**
Heterogeneity: not applicable						
Test for overall effect: t_{Inf} = 0.88 (P = 0.381)						
**Total (95% CI)**			**45**	**43**	**100.0%**	**0.15 [-1.57; 1.87]**
Heterogeneity: Tau^2 = 0; Chi^2 = 0.40, df = 1 (P = 0.528); I^2 = 0%						
Test for overall effect: t_1 = 1.11 (P = 0.466)						
Test for subgroup differences: Chi^2 = 0.40, df = 1 (P = 0.528)						

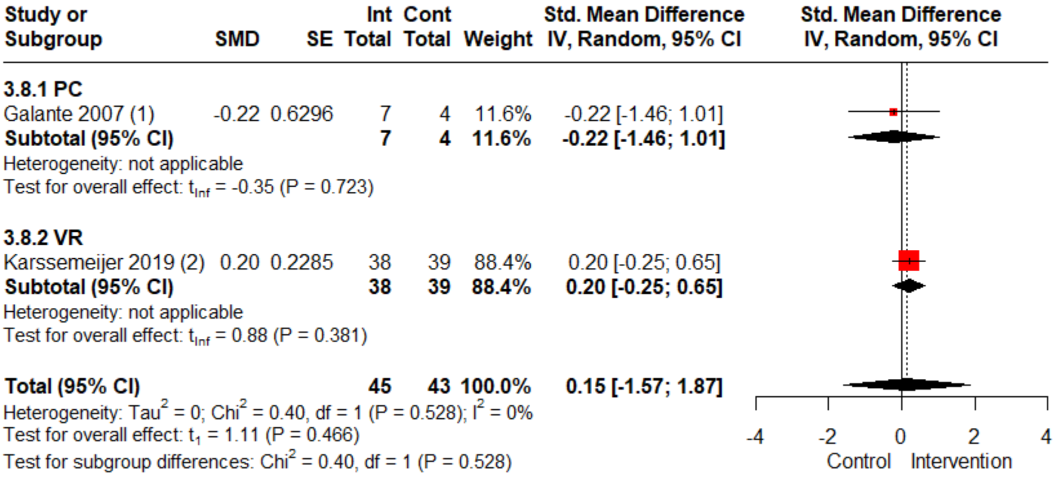

Footnote

(1) Digit cancellation test

(2) Composite score (n=2)

Figure 55: Meta-analysis; Computer-based cognitive interventions (DEMENTIA) vs. control follow-up (3 months), Outcome: **Attention/concentration/processing speed**

Study	Intervention Mean	SD	Total	Control Mean	SD	Total	Weight	Std. Mean Difference IV, Random, 95% CI
Karssemeijer 2019 (1)	-55.80	54.00	38	-68.50	73.40	39	50.0%	0.19 [-0.25; 0.64]
Karssemeijer 2019 (2)	-55.00	26.10	38	-61.40	36.10	39	50.0%	0.20 [-0.25; 0.65]
**Total (95% CI)**			**76**			**78**	**100.0%**	**0.20 [0.16; 0.24]**

Heterogeneity: $Tau^2 = 0$; $Chi^2 = 0.00$, df = 1 (P = 0.99); $I^2 = 0\%$
Test for overall effect: $t_1 = 66.08$ ($P < 0.01$)

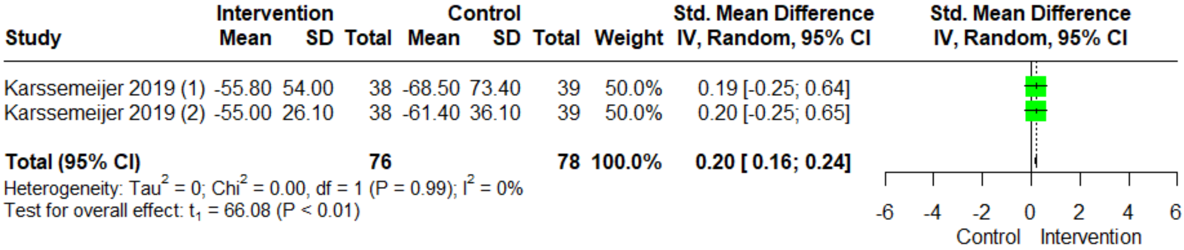

Footnote

(1) TMT A
(2) Stroop Color-Word Test (SCWT) – word reading (sec)

Figure 56: Composite score; Attention/concentration/processing speed_Karssemeijer 2019 (see Figure 55)

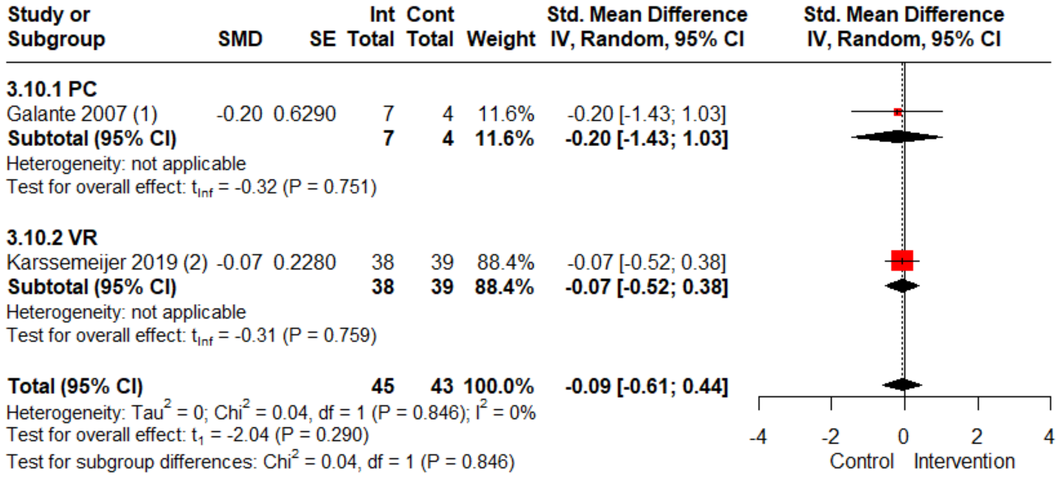

Footnote

(1) Composite score (n= 2)
(2) Composite score (n= 2)

Figure 57: Meta-analysis; Computer-based cognitive interventions (DEMENTIA) vs. control follow-up (3 months), Outcome: **Executive functioning**

Study	Intervention Mean	Intervention SD	Intervention Total	Control Mean	Control SD	Control Total	Weight	Std. Mean Difference IV, Random, 95% CI	Std. Mean Difference IV, Random, 95% CI
Galante 2007 (1)	11.40	3.30	7	12.10	4.00	4	50.1%	-0.18 [-1.41; 1.05]	
Galante 2007 (2)	30.60	6.40	7	32.80	12.30	4	49.9%	-0.23 [-1.46; 1.01]	
**Total (95% CI)**			**14**			**8**	**100.0%**	**-0.20 [-0.51; 0.10]**	

Heterogeneity: $Tau^2 = 0$; $Chi^2 = 0.00$, df = 1 (P = 0.96); $I^2 = 0\%$
Test for overall effect: $t_1 = -8.55$ (P = 0.07)

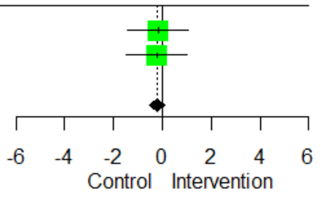

Footnote

(1) Semantic verbal fluency
(2) Phonemic verbal fluency

Figure 58: Composite score; Executive functioning_Galante 2007 (see Figure 57)

Study	Intervention Mean	Intervention SD	Intervention Total	Control Mean	Control SD	Control Total	Weight	Std. Mean Difference IV, Random, 95% CI
Karssemeijer 2019 (1)	-178.10	105.40	38	-166.10	105.10	39	33.3%	-0.11 [-0.56; 0.33]
Karssemeijer 2019 (2)	20.90	11.20	38	21.70	15.10	39	33.3%	-0.06 [-0.51; 0.39]
Karssemeijer 2019 (3)	-9.20	5.50	38	-8.90	6.70	39	33.4%	-0.05 [-0.50; 0.40]
**Total (95% CI)**			**114**			**117**	**100.0%**	**-0.07 [-0.16; 0.01]**

Heterogeneity: $Tau^2 = 0$; $Chi^2 = 0.05$, df = 2 (P = 0.98); $I^2 = 0\%$
Test for overall effect: $t_2 = -3.70$ (P = 0.07)

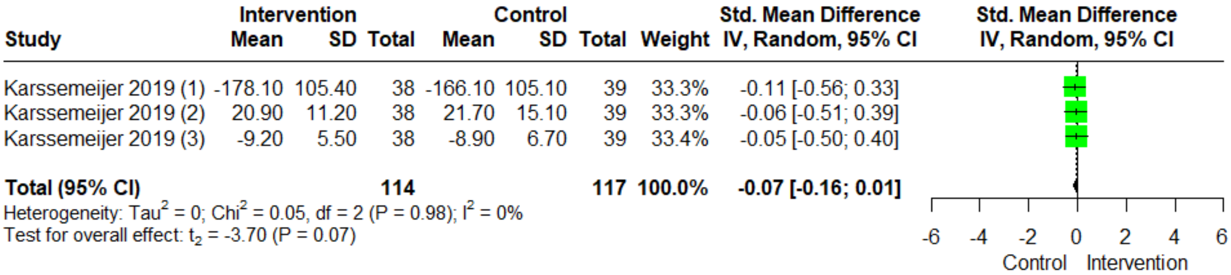

Footnote

(1) TMT B
(2) Verbal fluency test, letter fluency
(3) Rule Shift Cards Test, errors

Figure 59: Composite score; Executive functioning_Karssemeijer 2019 (see Figure 57)
